# The estimated distribution of autochthonous leishmaniasis by *Leishmania infantum* in Europe in 2005–2020

**DOI:** 10.1371/journal.pntd.0011497

**Published:** 2023-07-19

**Authors:** Carla Maia, Cláudia Conceição, André Pereira, Rafael Rocha, Maria Ortuño, Clara Muñoz, Zarima Jumakanova, Pedro Pérez-Cutillas, Yusuf Özbel, Seray Töz, Gad Baneth, Begoña Monge-Maillo, Elkhan Gasimov, Yves Van der Stede, Gregorio Torres, Céline M. Gossner, Eduardo Berriatua

**Affiliations:** 1 Global Health and Tropical Medicine, Instituto de Higiene e Medicina Tropical, Universidade NOVA de Lisboa, Lisbon, Portugal; 2 Departamento de Sanidad Animal, Facultad de Veterinaria, Regional Campus of International Excellence “Campus Mare Nostrum”, Universidad de Murcia, Murcia, Spain; 3 SaBio, Institute for Game and Wildlife Research, IREC (CSIC-UCLM-JCCM), Ciudad Real, Spain; 4 Departamento de Geografía, Universidad de Murcia, Murcia, Spain; 5 Department of Parasitology, Faculty of Medicine, Ege University, Izmir, Turkey; 6 Koret School of Veterinary Medicine, The Hebrew University of Jerusalem, Rehovot, Israel; 7 Unidad de Referencia Nacional para Enfermedades Tropicales, Servicio de Enfermedades Infecciosas, Hospital Universitario Ramón y Cajal, IRYCIS, Madrid, Spain; 8 Division of Country Health Programmes, World Health Organization Regional Office for Europe, Copenhagen, Denmark; 9 Biological Hazards, Animal Health and Welfare (BIOHAW) Unit, European Food Safety Authority, Parma, Italy; 10 Science Department, World Organisation for Animal Health (WOAH), Paris, France; 11 Disease Programme Unit, European Centre for Disease Prevention and Control (ECDC), Stockholm, Sweden; Universidade Federal de Minas Gerais, BRAZIL

## Abstract

**Background:**

This study describes the spatial and temporal distribution between 2005 and 2020 of human and animal leishmaniasis by *Leishmania infantum* in European countries reporting autochthonous cases, and highlights potential activities to improve disease control.

**Methodology/Principal findings:**

It was based on a review of the scientific literature and data reported by the World Health Organization (WHO), the World Organization for Animal Health (WOAH) and the Ministries of Health, including hospital discharges in some countries. Autochthonous infections were reported in the scientific literature from 22 countries, including 13 and 21 countries reporting human and animal infections, respectively. In contrast, only 17 countries reported autochthonous human leishmaniasis cases to the WHO and 8 countries animal infections to the WOAH. The number of WOAH reported cases were 4,203, comprising 4,183 canine cases and 20 cases in wildlife. Of 8,367 WHO reported human cases, 69% were visceral leishmaniasis cases—of which 94% were autochthonous—and 31% cutaneous leishmaniasis cases—of which 53% were imported and mostly in France. The resulting cumulative incidence per 100,000 population of visceral leishmaniasis between 2005–2020, was highest in Albania (2.15 cases), followed by Montenegro, Malta, Greece, Spain and North Macedonia (0.53–0.42), Italy (0.16), Portugal (0.09) and lower in other endemic countries (0.07–0.002). However, according to hospital discharges, the estimated human leishmaniasis incidence was 0.70 in Italy and visceral leishmaniasis incidences were 0.67 in Spain and 0.41 in Portugal.

**Conclusions/Significance:**

Overall, there was no evidence of widespread increased incidence of autochthonous human leishmaniasis by *L*. *infantum* in European countries. Visceral leishmaniasis incidence followed a decreasing trend in Albania, Italy and Portugal, and peaked in Greece in 2013, 2014 and 2017, and in Spain in 2006–2007 and 2011–2013. Animal and human cutaneous leishmaniasis remain highly underreported. In humans, hospital discharge databases provide the most accurate information on visceral leishmaniasis and may be a valuable indirect source of information to identify hotspots of animal leishmaniasis. Integrated leishmaniasis surveillance and reporting following the One Health approach, needs to be enhanced in order to improve disease control.

## Author summary

Leishmaniasis caused by the protozoan parasite *Leishmania infantum*, is a zoonotic sand fly-transmitted disease endemic in southern Europe, of great medical and veterinary importance. Environmental and human-related changes affecting the risk of exposure to vectors, such as travelling of infected animals, urbanization of endemic rural areas and climate change are some examples of drivers of the epidemiology of leishmaniasis. In this paper, we review the distribution and assess the evidence of emergence of human and animal leishmaniasis between 2005 and 2020 in endemic European countries. We found autochthonous infections in every Mediterranean and Balkan countries with a highly variable incidence. However, we detected no evidence of a widespread increased incidence across Europe. Notwithstanding this, underreporting is very high for animal and cutaneous human leishmaniasis. Moreover, human hospital discharges are the most accurate source of data for estimating the incidence of severe human visceral leishmaniasis, and this could be used to identify hotspots of leishmaniasis in animal reservoirs of infection. Consequently, we encourage integrated surveillance and accurate notification of cases following the One Health approach to adopt effective strategies resulting in a better control of leishmaniasis.

## Introduction

The leishmaniases are a group of zoonotic diseases caused by *Leishmania* spp. which are protozoan parasites, transmitted by phlebotomine sand flies. They are endemic in tropical and subtropical climates including southern Europe. Among the 31 *Leishmania* spp. that infect humans and animals worldwide [1], *Leishmania infantum* is the only autochthonous species in Europe, except for sporadic cases of local transmission of *Leishmania tropica* in Greece [2] and *Leishmania donovani sensu stricto* in Cyprus [3]. Dogs are the main domestic infection reservoir of *L*. *infantum* and the most susceptible host species, whilst humans are considered an accidental host. Canine leishmaniasis (CanL) is typically a multi-organ and fatal disease unless promptly treated. Human leishmaniasis (HumL) by *L*. *infantum* takes distinct clinical forms of variable severity: visceral leishmaniasis (VL) which is a life-threatening condition, cutaneous leishmaniasis (CL) which is most commonly localized and self-healing and, mucosal leishmaniasis (ML) which is a rare and serious condition affecting the nasal and pharyngo-laryngeal mucosa, distinct from mucocutaneous leishmaniasis (MCL) caused by American *Leishmania* spp. [4].

The distribution of leishmaniasis is dynamic spatially and temporarily, subjected to societal and environmental-related factors, including movement of infected animals and people, changes in the incidence of comorbidities such as human immunodeficiency virus infections, urbanization of vector endemic areas and climate change [5–9]. In 2020, the European Centre for Disease Prevention and Control (ECDC) commissioned a review of the recent epidemiology and control of human and animal leishmaniasis in the European Union and neighboring countries. In a questionnaire survey, the majority of public health and veterinary authorities in these countries considered leishmaniasis a neglected, emergent disease with insufficient resources dedicated to its prevention and control [10]. Emergence was defined in the questionnaire as the establishment or increased incidence of infection/disease in an endemic area, which may or may not be the result of improved and wider diagnosis [10]. To assess the emergence of HumL and CanL in Europe, it is necessary to analyze the spatial and temporal incidence trends. Therefore, and as part of the ECDC commissioned review, we collected and analyzed published data on the spatial distribution of autochthonous leishmaniasis, and on the frequency of leishmaniasis cases reported by national and international human and animal health competent authorities. This article summarizes the main findings of this study for European countries including: (a) the spatial distribution of autochthonous infections by *L*. *infantum* in animals and humans based on scientific information published between 2009 and 2020, (b) the incidence and evidence of emergence of human visceral and cutaneous leishmaniasis according to cases reported to the World Health Organization (WHO) and the Ministries of Health (MoH) of some countries, between 2005 and 2020, (c) the frequency of animal leishmaniasis outbreaks and cases reported to the World Organization for Animal Health (OIE-WAHIS) between 2005 and 2020, and (d) the potential future activities to prevent and control the disease in the region.

## Materials and methods

### Literature review on autochthonous *L*. *infantum* infections in humans, animals and vectors in Europe

Autochthonous infections were those reported in the literature as acquired in the patients’ country of residence when relating to humans and domestic animals, or in the country where animals were captured and analyzed when referring to wildlife vertebrates and arthropods. Imported cases were those deemed to have become infected outside the country. European countries considered included every country in the European continent with the exception of Russia and Turkey. Documents to characterize the spatial distribution of autochthonous leishmaniasis in humans and animals in Europe were selected among those retrieved as part of a wider, comprehensive review of leishmaniasis epidemiology in Europe and neighboring countries, based on scientific literature published between January 2009 and July 2020. The search protocol, included scientific articles in the SCOPUS database, PhD and MSc thesis and information on national and international human and animal health institution websites, as previously described [11]. The SCOPUS search included two Boolean search strings on: (1) leishmaniasis epidemiology, diagnosis, treatment and control, and (2) *Leishmania* infection rates in sand flies. The PRISMA figure depicting the flow of documents retrieved and those used to map autochthonous leishmaniasis infections is provided as Supporting Information.

### World Health Organization and Ministry of Health reports of autochthonous and imported cutaneous and visceral human leishmaniasis in Europe

The incidence of HumL was analyzed for 17 European countries reporting autochthonous VL and/or CL cases to the World Health Organization Global Health Observatory data repository (WHO-GHO) (https://apps.who.int/gho/data/node.main.NTDLEISH?lang=en) between 2005 and 2020. Two sources of data were used: (i) the WHO-GHO and (ii) databases of the MoH of Bulgaria, France, Greece, Italy, Malta, Portugal and Spain for variable periods between 2005 and 2020. The latter were provided by the ECDC national focal points (Public Health institutes in the EU member states), except for Italy and Spain which were obtained from Tilli et al. [12] and the Spanish Institute of Health Information (http://www.mscbs.gob.es/estadEstudios/estadisticas/estadisticas/estMinisterio/SolicitudCMBDdocs/2018_ANEXO_solicitud_RAE_CMBD.pdf), respectively. Cases from Spain, Italy, Bulgaria, Malta and Portugal were obtained from their hospital discharge databases, and those of France and Greece from their national epidemiological networks. It was not possible to obtain data from the Ministries of Health of other endemic countries.

The frequency of cases reported by the WHO between 2004 and 2008 has already been published before [13], and cases reported between 2005 and 2008 were included in the present study to analyze the complete temporal trend available in the WHO-GHO. In this data set, autochthonous and imported cases were reported together between 2005 and 2012 and separately between 2013 and 2020, and CL cases include patients with cutaneous or mucosal disease.

The detail of available ministerial HumL data varied between countries. Bulgaria, Greece, Malta and Spain provided individual VL and CL (Greece and Spain only) cases for the periods 2009–2020, 2009–2018, 2009–2020 and 2005–2020, respectively, and included patient gender, age (in different categories depending on the country) and residence at the level 3 of the Nomenclature of Territorial Units for Statistics (NUTS-3). The NUTS classification is a hierarchical system for dividing up countries of the EU for administrative and statistical purposes (https://ec.europa.eu/eurostat/web/nuts/background). Italy reported the frequency of leishmaniasis cases (VL and CL together) at the NUTS-2 level of the hospital where the patient was diagnosed. France and Portugal reported the countries’ annual frequency of VL and CL (France only). Bulgaria also provided the patient´s anti-*Leishmania* treatment used, and Italy, Malta and Spain provided the patient comorbidities, including the HIV infection status, the only morbidity considered in the present study.

### World Organization for Animal Health reports of animal leishmaniasis outbreaks and cases in domestic and wildlife in Europe

The frequency of leishmaniasis outbreaks and cases in domestic animals and wildlife in endemic countries between 2005 and 2020 reported by the World Organization for Animal Health (OIE-WAHIS) was obtained from https://wahis.woah.org/#/dashboards/country-or-disease-dashboard. According to WOAH glossary, cases are individual animals infected by a pathogenic agent, with or without clinical signs, and outbreaks refer to the occurrence of one or more cases in an epidemiological unit (https://www.woah.org/en/what-we-do/standards/codes-and-manuals/terrestrial-code-online-access/?id=169&L=1&htmfile=glossaire.htm#terme_cas).

### Statistical analysis and mappings

The geographical origin of autochthonous *L*. *infantum* infections and leishmaniasis cases in humans and animals reported in the literature were coded and mapped at NUTS-3 level for EU countries and at the levels 1 or 2 of the Global Administrative Unit Layers (GAUL) (https://data.review.fao.org/map/catalog/srv/api/records/7e6357e6-0893-4b61-a26d-eb09a04eed72) for Bosnia and Herzegovina (GAUL-2), Kosovo (GAUL-1), Montenegro (GAUL-1) and Ukraine (GAUL-1), using the geographical information system (GIS) ArcGIS v.10 software (ESRI, Redlands, USA). Mapping codes were obtained from the ECDC Vector XY Location digital application (https://gis.ecdc.europa.eu/portal/apps/webappviewer/index.html?id=e41fb4bd32fb4a57be8dded357e88115). Designation of Kosovo is without prejudice to positions on status and is in line with UNSCR 1244/1999 and the ICJ Opinion on the Kosovo Declaration of Independence.

The annual cumulative incidences per 100,000 population (incidence, hereafter) of HumL cases reported by WHO and Health Ministries were calculated by multiplying the annual number of cases by 100,000 and dividing by the countries’ or region’ annual population census. Censuses were obtained from Eurostat (https://appsso.eurostat.ec.europa.eu/nui/show.do?dataset=demo_r_gind3&lang=en) and from the World Bank (https://data.worldbank.org/indicator/SP.POP.TOTL) when not available from Eurostat. The numerical data used in all figures are included in S1 Data. The non-parametric Kruskal-Wallis test was used to compare median incidences between time periods. Differences were deemed significant for p<0.05 and marginally significant for p<0.10 for a two-tailed test. The analysis was carried out using the statistical program R [14].

## Results

### Spatial distribution of autochthonous *L*. *infantum* infections in humans and animals in Europe

The number of documents reviewed that provided information on autochthonous *L*. *infantum* infections (with or without clinical signs and species identification) were 695 documents (S1 Appendix). They included mostly scientific articles and PhD and MSc thesis as well as information available in the WHO and WOAH web pages and that provided by the MoH of the above-mentioned countries. National governmental organizations webpages were not found to be, in general, very useful in finding data from leishmaniasis, and very scarce evidence was found on the existence of specific programs on the disease or on assessments of the national situation.

**Fig 1:**
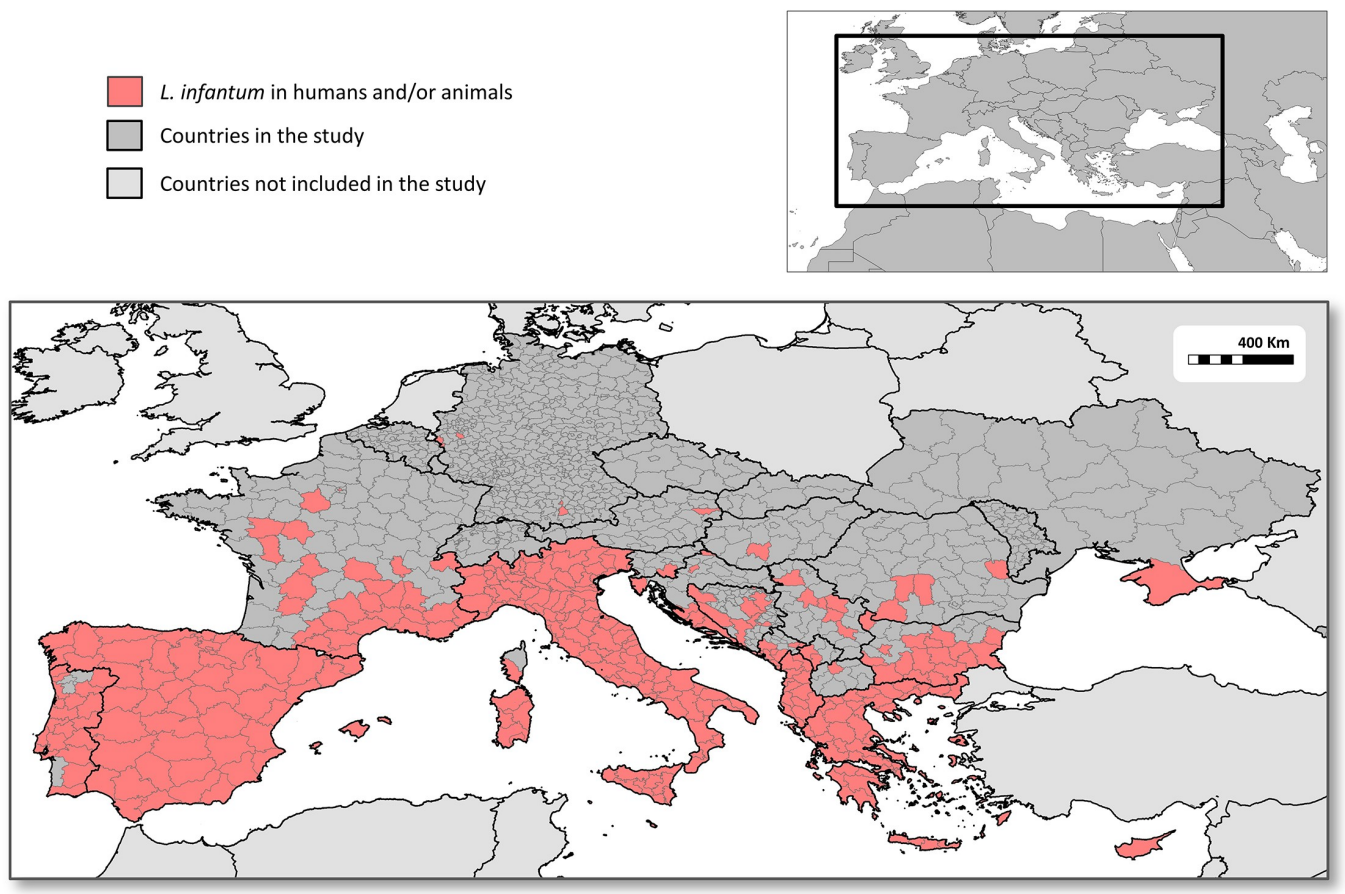
Regional distribution of autochthonous *Leishmania infantum* infections in humans and animals in European countries reported in the scientific literature and by the ministries of health of some countries, between 2009 and 2020.

Infections in animals and/or humans were reported from 22 countries including Albania, Austria, Bosnia and Herzegovina, Bulgaria, Croatia, Cyprus, France, Germany, Greece, Hungary, Italy, Kosovo, Malta, Montenegro, North Macedonia, Portugal, Romania, San Marino, Serbia, Slovenia, Spain, and Ukraine (Figs 1 and S1). Animal infections were described in all the 22 countries mentioned above, except in Ukraine. Human infections were described in 13 countries: Albania, Bulgaria, Croatia, France, Germany, Greece, Italy, Malta, Montenegro, Portugal, Serbia, Spain, and Ukraine (S1 Fig).

The number of NUTS-3, GAUL-1 and GAUL-2 territorial subdivisions in which infections in animals, humans or either were reported were 286, 214 and 341 subdivisions, respectively, and humans and animals only shared 47% (159/341) of them (S2 Table). There were large differences between countries in the percentage of territorial subdivisions where infections were reported from (Fig 1 and Table 1). Leishmaniasis was reported in animals and/or humans from all or the majority of territorial subdivisions in Portugal, Spain, Southeast France and Corsica, Italy, Malta, Greece, Cyprus, Albania, the Adriatic coast of Croatia and Montenegro and southern Bulgaria. In contrast, reports of infections were found from only one subdivision in Austria, Hungary, Slovenia, North Macedonia and Ukraine (in Crimea), two in Kosovo, four in Germany and Romania, six in Serbia and eight in Bosnia and Herzegovina (Fig 1 and Table 1).

**Table 1:** Absolute and relative frequencies of territorial subdivisions in Europe where autochthonous *Leishmania infantum* infections in humans and/or animals were reported in the scientific literature between 2009 and 2020.

	Territorial subdivisions				
Country	Class	No. of units	No. of units with cases	% of units with cases	References
Albania	NUTS-3	12	12	100	[15–20]
Austria	NUTS-3	35	1	3	[21]
Bosnia and Herzegovina	GAUL-2	81	8	10	[22,23]
Bulgaria	NUTS-3	28	17	61	[24], Bulgarian MoH^b^
Croatia	NUTS-3	21	7	33	[25–30]
Cyprus	NUTS-3	1	1	100	[31]
France	NUTS-3	101	29	29	[32–42]
Germany	NUTS-3	401	4	1	[43]
Greece	NUTS-3	52	52	100	[44–55], Hellenic MoH^c^
Hungary	NUTS-3	20	1	5	[56]
Italy	NUTS-3	110	110	100	[12,57–104]
Kosovo^a^	GAUL-1	5	2	40	[105]
Malta	NUTS-3	2	2	100	[106–108]
Montenegro	GAUL-1	21	10	48	[109]
North Macedonia	NUTS-3	8	1	13	[110]
Portugal	NUTS-3	25	19	76	[111–120]
Romania	NUTS-3	42	4	10	[121–124]
San Marino	NUTS-3	1	1	100	[125]
Serbia	NUTS-3	25	6	24	[126–128]
Slovenia	NUTS-3	12	1	8	[129]
Spain	NUTS-3	59	52	88	[130–153], CMBD^d^
Ukraine	GAUL-1	25	1	4	[154]

^a^Kosovo: this designation is without prejudice to positions on status and is in line with UNSCR 1244/1999 and the ICJ Opinion on the Kosovo Declaration of Independence.

^b^Ministry of Health of Bulgaria. Data provided by the National Centre for Infectious and Parasitic Diseases.

^c^Ministry of Health of Greece. Data provided by the Hellenic National Public Health Organization in the context of a technical report commissioned and coordinated by ECDC.

^d^National Information System for Hospital Data (Conjunto Mínimo Básico de Datos-CMBD). Data from 2005 to 2020 requested to the Unit of Health Care Information and Statistics, Institute of Health Information, Spanish Ministry of Health, by e-mail (icmbd@sanidad.gob.es) in February 2022.

### Incidence of autochthonous and imported human leishmaniasis in Europe between 2005 and 2020 reported by the WHO

**Fig 2:**
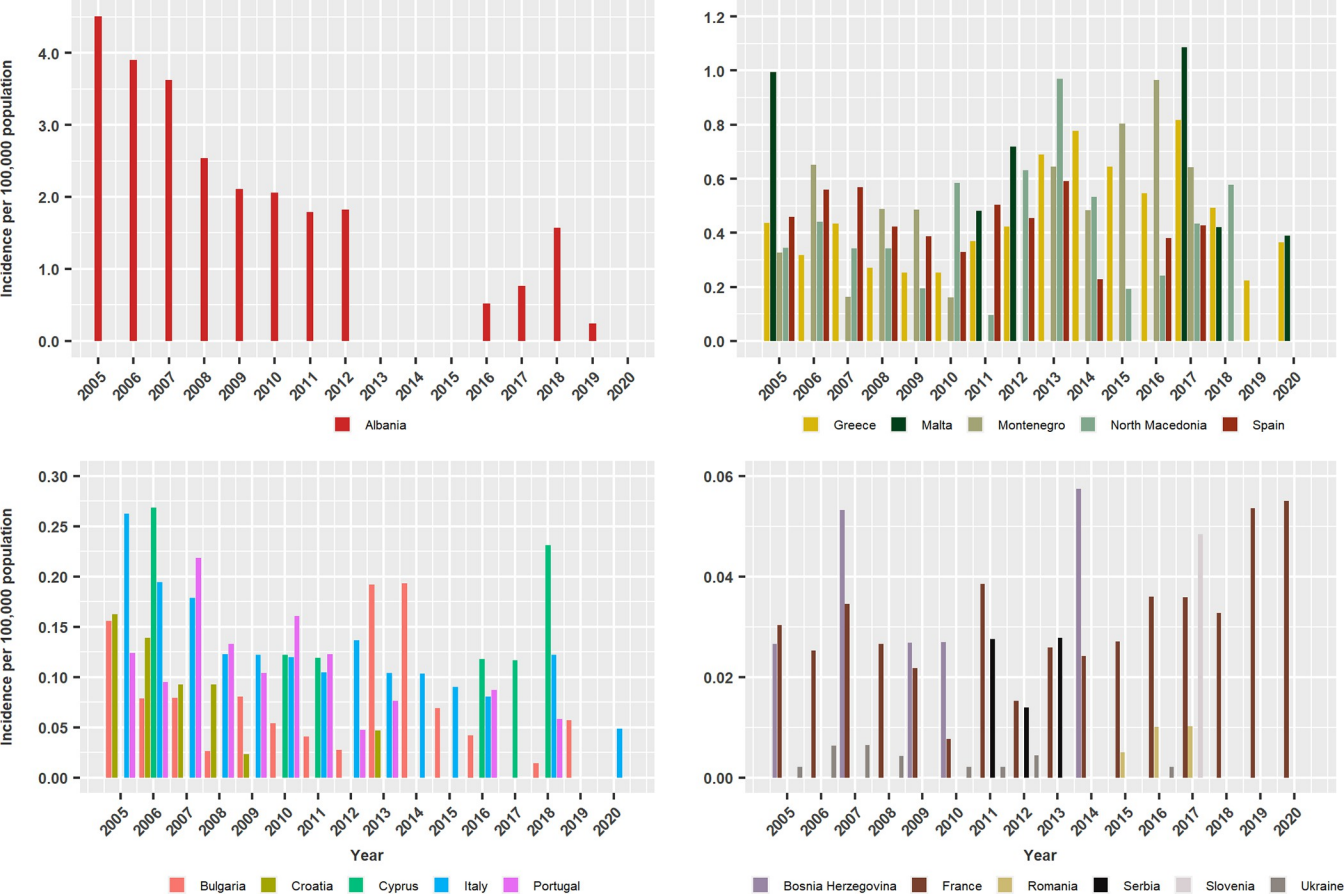
Annual cumulative incidence per 100,000 population of human autochthonous visceral leishmaniasis in European countries between 2005 and 2020. Data based on cases reported to the WHO Global Health Observatory Data Repository.

A total of 8,367 HumL cases including 5,813 VL (69%) and 2,554 CL (31%) cases, were reported to the WHO between 2005 and 2020 in the 17 European countries with autochthonous cases including Albania, Bosnia and Herzegovina, Bulgaria, Croatia, Cyprus, France, Greece, Italy, Malta, Montenegro, North Macedonia, Portugal, Romania, Serbia, Slovenia, Spain, and Ukraine (Fig 2 and S1 Table). The total number of cases reported between 2013 and 2020 was 4,399 and they included 3,191 (73%) autochthonous infections (1,496 VL and 1,695 CL cases) and 1,208 (27%) imported infections (121 VL cases and 1,087 CL cases). Most imported cases (79 VL and 985 CL cases) were reported by France. The annual number of imported CL cases in France ranged between 74 cases in 2016 and 176 in 2018, and the median of the annual number of CL cases was significantly higher in 2017–2020 compared to 2013–2016, and so was the ratio of the incidence of imported versus (vs.) autochthonous cases between these two periods (p<0.05). Similarly, the annual number of imported VL cases in France ranged between 5 cases in 2014 and 2018 and 14 cases in 2019, and the median of the annual number of VL cases and ratio of the incidence of imported vs. autochthonous cases, was similar in 2013–2016 compared and 2017–20 (p>0.05). Other imported cases in Europe, in decreasing order, were from Greece, Spain, Bulgaria, Italy, Romania, Portugal and Ukraine and differences in the median annual number and the ratio of imported vs. autochthonous CL and VL cases were not significant (p>0.05).

The overall reported cumulative VL incidence between 2005 and 2020 was highest, 2.15 cases per 100,000 population in Albania, it ranged between 0.53 and 0.42 in Montenegro, Malta, Greece, Spain and Northern Macedonia, it was 0.16 in Italy, 0.09 in Portugal and ranged between 0.07 and 0.002 in the remaining countries. However, the number of years when cases were reported varied substantially, ranging between 16 years for Greece and Bulgaria and 4 years for Romania (S1 Table). Fig 2 shows the annual incidences in years when cases were reported, having grouped countries into four charts of decreasing reported incidence. Incidence followed a decreasing trend in Albania from 4.5 cases in 2005 to 1.8 cases in 2012, it ranged between 0.5 and 1.6 in 2016 and 2019, and no cases were reported between 2013 and 2015 and in 2020. Similarly decreasing trends were also observed in Croatia, Italy and Portugal (Fig 2 and S1 Table). Temporal trends in other countries fluctuated more. For example, they peaked in Spain in 2007, 2013 and 2017, and in Greece in 2013 and 2017. No sustained trends of increasing incidence were observed in any country (Fig 2 and S1 Table).

**Fig 3:**
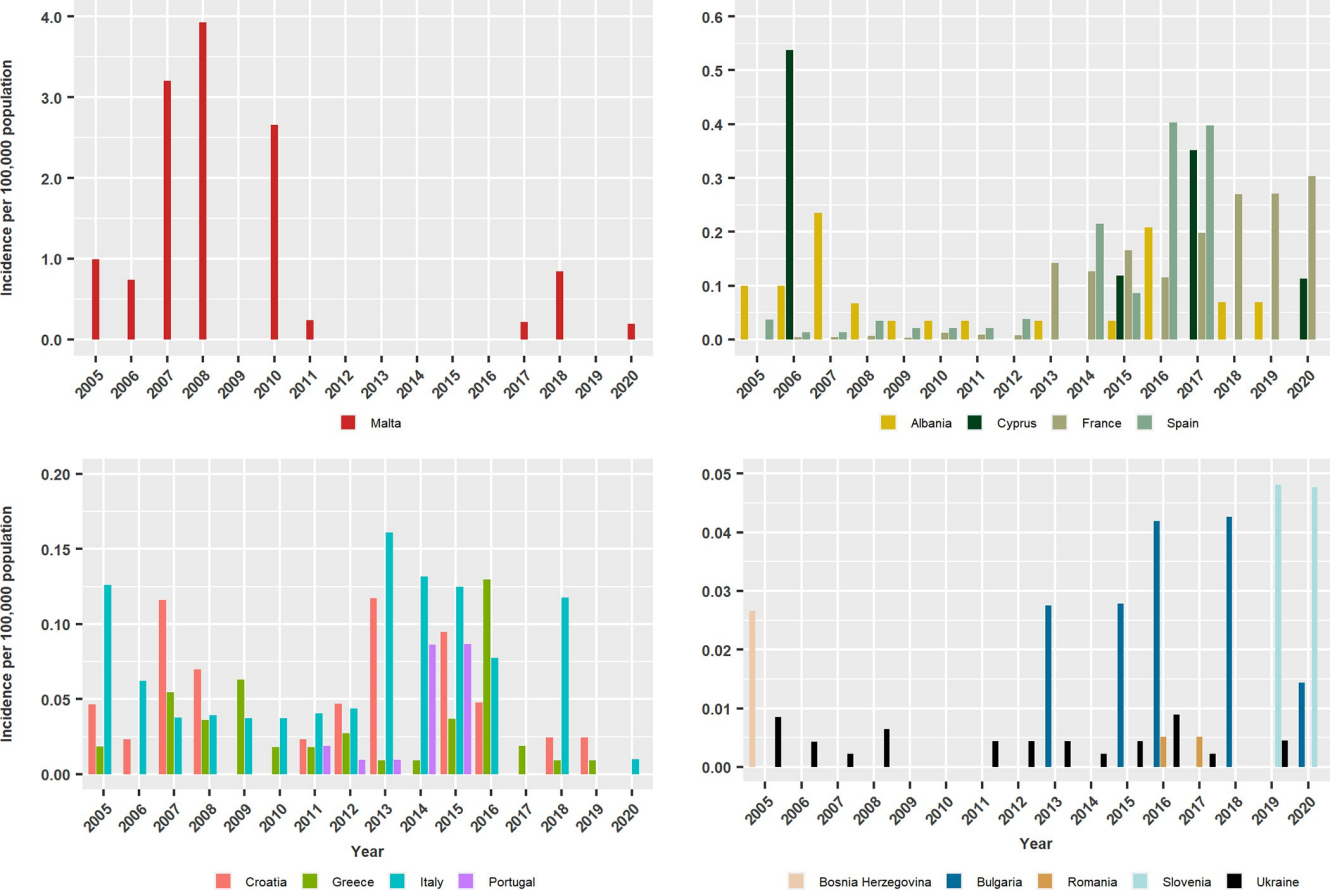
Annual cumulative incidence per 100,000 population of autochthonous human cutaneous leishmaniasis in European countries between 2005 and 2020. Data based on cases reported to the WHO Global Health Observatory Data Repository.

Reported cumulative CL incidence in the 2005 to 2020 period was 0.95 in Malta, 0.11–0.10 in Spain, Cyprus and France, 0.07–0.06 in Italy, Albania and Croatia, 0.04–0.02 in Portugal, Greece, Bulgaria and Slovenia, 0.004–0.003 in Ukraine, Bosnia and Herzegovina and Romania and it was not reported in Montenegro, North Macedonia and Serbia (Fig 3 and S1 Table). However, the number of years when CL cases were reported varied greatly between countries, ranging from 1 to 16 years (S1 Table). Except in Malta and Cyprus where CL incidences were highest in 2008 (3.92 cases) and 2006 (0.54 cases), respectively, peaks in other countries were most frequent between 2013 and 2020, and this was particularly evident in Spain, France and Italy (Fig 3).

### Incidence and demographic characteristics of human leishmaniasis reported by the Ministries of Health of Bulgaria, France, Greece, Italy, Malta, Portugal and Spain

#### Bulgaria

Between 2009 and 2020 the Bulgarian MoH reported 58 VL cases in 55 patients (3 patients had relapses), compared to 56 VL cases reported by WHO-GHO in this period. Fifty patients had autochthonous infections and five patients had imported infections. The cumulative VL incidence in this period, excluding relapses and imported cases, was 0.06 cases per 100,000 population, it was highest in 2013 (0.18), followed by 2014 (0.15), and ranged between 0.01 and 0.08 in other years, except in 2017 and 2018 when no autochthonous cases were reported. There was no evidence of significant temporal changes in VL incidence. Autochthonous cases resided in 10 of 28 NUTS-3 subdivisions in Bulgaria, where incidence was 0.73 in Blagoevgrad, 0.17 in Silven, 0.11 in Kardzhali, 0.10 in Plovdiv, 0.09 in Vidin, 0.07 in Gabrovo and Smolyan, 0.03 in Veliko-Tarnovo and 0.02 in Burgas and Sofia.

Males represented 62% of VL patients, and age-specific case percentages were 22%, 4%, 69% and 5% among 0–4, 5–14, 15–64 and >64 years old, respectively. Patients were treated with meglumine antimoniate except one case in 2010 that was treated with miltefosine and one case in 2020 with liposomal amphotericin B.

#### France

Data from the MoH was available for autochthonous infections only and the number of cases reported between 2009 and 2020 was 200 cases, compared to 149 cases by WHO-GHO. Cases were 133 VL, 51 CL and 16 ML cases. The corresponding country`s cumulative incidences during this period were 0.017 VL cases, 0.006 CL cases and 0.002 ML cases per 100,000 population. Annual incidences were equal or above these figures in 2009 (0.022), 2011 (0.038), 2014 (0.017) and 2019 (0.019) and 2020 (0.022) for VL, and between 2010 and 2015 (0.006–0.012) and in 2020 (0.013) for CL.

#### Greece

The number of HumL cases reported by the MoH between 2009 and 2018 were 633 cases, similar to those reported in WHO-GHO in the same period (S1 Table). They included 598 VL (94%) and 35 CL (6%) cases, out of which 534 VL (89%) and 10 CL (29%) were autochthonous infections. The country´s cumulative incidence of autochthonous VL in 2009–2018 was 0.49 cases per 100,000 population, and years with incidence above these figures included 2013 (0.74), 2014 (0.78), 2015 (0.52) and 2017 (0.72). The median annual incidence of autochthonous VL was significantly higher in 2013–2018 (0.63) compared to 2009–2012 (0.31) (p<0.05).

Autochthonous VL in 2009–2018 was reported from 46 of 52 NUTS-3 subdivisions in Greece, and those with the highest incidence were Larisa (2.58), Karditsa (1.55) and Dytiki Attiki (1.45). Median incidence of autochthonous VL was higher in 2013–2018 compared to 2009–2012 in the NUTS-2 subdivisions of the Peloponnese and in Thessaly (p<0.05) and did not change significantly elsewhere (p>0.05).

The proportion of males among VL and CL patients were 63% and 60%, respectively. The age distribution of VL patients was 14% for 0–4 years old, 9% for 5–14 years old, 51% for 15–65 years old and 26% for older patients. Similarly, for CL, percentages were 12%, 27%, 50% and 19%, respectively.

#### Italy

**Fig 4:**
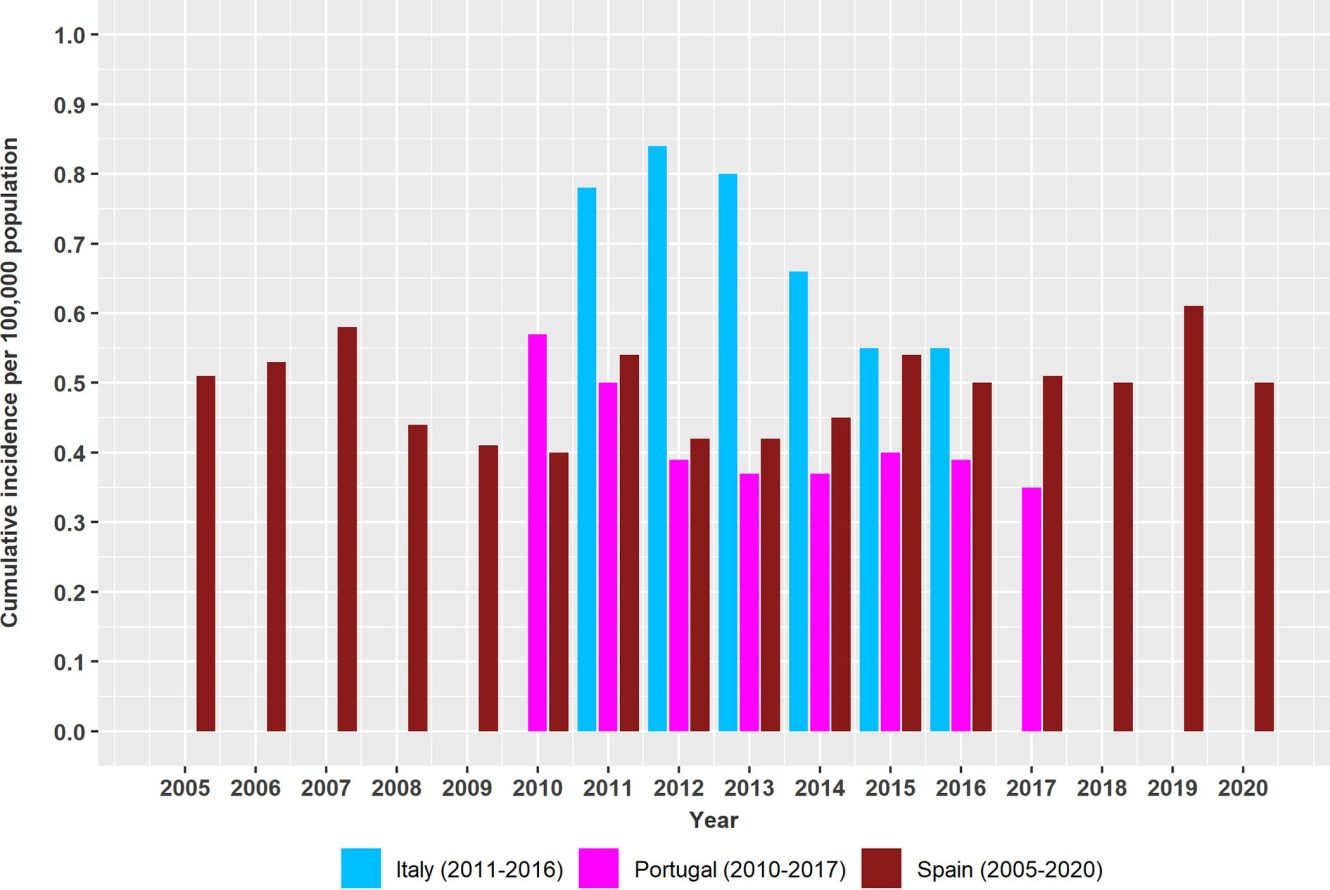
Annual cumulative incidence per 100,000 population of human leishmaniasis between 2005 and 2020 reported by the Ministries of Health of Italy, Portugal, and Spain.

Between 2011 and 2016 hospitals reported 2,509 HumL patients, 2,203 (88%) were Italian citizens and 306 (12%) were from other countries [12]. This number contrasts with the 721 cases reported in the WHO-GHO data set in the same period (S1 Table), and differences between sources were not constant across years (ranging between 204 and 390 cases per year). However, both sources reveal a decreasing incidence trend (Figs 2 and 4). The clinical form of leishmaniasis was specified for 84% of the hospital cases and included VL (82%), CL (14%) and MCL (2%). The corresponding countries’ cumulative incidence between 2011 and 2016 was 0.70 (0.61 considering Italian citizens only) cases per 100,000 population. The annual incidence was highest in 2012 with 0.84 cases and decreased thereafter to 0.55 in 2015 (Fig 4). Median incidence in 2014–2016 was significantly lower compared to 2011–2013 (p<0.05).

Regional incidence of HumL cases in 2011–2016 was above the national average in Sicily (1.93), Liguria (1.59), Sardinia (1.09), Emilia-Romagna (1.04), Lazio (0.84), Campania (0.80), Calabria (0.78) and Tuscany (0.74). Median incidence was significantly lower in 2014–2016 compared to 2011–2013 in Sicily and Lazio (p<0.05) and marginally lower in Tuscany (p<0.10) and did not change significantly elsewhere.

Males accounted for 72% of patients and age-specific prevalence was 10% for 0–4 years old, 5% for the 5–14 years old, 4% for 15–24 years old, 61% for 25–64 years old and 19% for older people. Fifty three percent of HumL patients presented other pathological conditions including HIV infections in 27% of patients [12].

#### Malta

The MoH of Malta reported 20 VL cases between 2009 and 2020, compared to 14 cases in the WHO-GHO data set (S1 Table), and none were reported in 2013, 2016 and 2020. The number of autochthonous cases was not specified, and patients included 15 Maltese and two foreign citizens, and the citizenship of the remaining three cases was not provided. The country’s cumulative incidence over the study period was 0.37 cases per 100,000 population. Excluding years with no cases, incidence ranged between 0.23 in 2015 and 0.72 in 2012. Median VL incidence in 2009–2014 and 2015–2020 were 0.49 and 0.32 (p>0.05).

Males represented 65% of patients, 11% were 0–4 years old, 6% were 5–14 years old, 60% were 15–64 years old and 23% were 65–96 years old. Five patients were reported to be HIV positive.

#### Portugal

Between 2010 and 2017, Portugal reported 689 hospital discharge cases from 346 patients and males accounted for 77% of patients. The number of cases reported was much higher than the 74 cases reported in the WHO-GHO for the same years (S1 Table). Annual differences were fairly constant (ranged between 31 and 42 cases), and both sources reveal a similar decreasing incidence trend (Figs 2 and 4).

The country´s HumL cumulative incidence during this period was 0.41 cases per 100,000 population. Annual incidence decreased from 0.57 in 2010 to 0.35 in 2017 (Fig 4). Median incidence was significantly lower in 2012–2017 compared to 2010–2011 (p<0.05).

#### Spain

The number of HumL hospital discharges between 2005 and 2020, including autochthonous and imported cases, was 8,260 for 4,946 patients (40% of patients had more than one discharge). The number of patients was greater than those reported by WHO-GHO (3,035 patients), and differences between sources varied considerably between years and so did the corresponding incidence trends (Figs 2 and 4). The clinical form was specified for 81% of patients and they included VL (91%), CL (6%) and ML (4%).

The cumulative incidence of reported hospital discharges in 2005–2020 was 0.67 cases per 100,000 population including 0.49 VL cases, 0.03 CL cases, 0.02 ML cases and 0.13 cases for which the clinical form was not specified. Annual VL incidence was greater than the mean annual VL incidence between 2005 and 2007, in 2011 and between 2015 and 2019 (Fig 4). Similarly, annual CL incidence was equal or above the mean CL incidence in 2005 and between 2016 and 2020 with incidence peaks of 0.09 in 2018 and 2019 and dropped to 0.05 in 2020. Highest ML incidence was above the mean in 2009, 2016 and between 2018 and 2020. The median VL incidence was higher in 2005–2007 compared to 2008–2010, and in 2016–2020 compared to 2012–2015, and the median CL and ML were higher in 2016–2020 compared to 2005–2015 (p<0.05).

Leishmaniasis was reported from most NUTS-3 subdivisions in Spain, and incidence of VL and CL rose significantly between 2005 and 2020 in the three NUTS-3 units (Alicante, Castellón and Valencia) in the NUTS-2 region of Comunidad Valenciana. The median HumL in this region was 0.63 in 2005–2008, 0.62 in 2009–2012, 0.96 in 2013–2016 and 1.48 in 2017–2020 (p<0.05). In contrast, incidence in the Catalonian region gradually decreased from 0.55 in 2005–2008 to 0.25 in 2013–2016 and rose to 0.34 in 2017–2020 (p<0.05).

Males accounted for 65% and 72% of CL and VL patients, respectively. The percentage of cases among 0–4 years old, 5–14 years old, 15–64 years old and 65–96 years old were 18%, 3%, 65% and 14% for VL and 10%, 5%, 56% and 29% for CL, respectively. The percentage of patients that were HIV positive was 31% and they accounted for 44% of all hospital discharges.

### Frequency of animal leishmaniasis between 2005 and 2020 reported to WOAH

Eight countries reported outbreaks and cases of animal leishmaniasis to the WOAH between 2005 and 2020, including Croatia, Greece, Italy, Montenegro, North Macedonia, Portugal, San Marino and Spain. They included 3,190 outbreaks involving 4,183 canine cases and 20 leishmaniasis cases in wildlife (Table 2). There was wide variation between these countries in the number of different years when cases were reported, ranging from twelve years in Croatia and Greece and only in one year in North Macedonia and San Marino, as well as in the number of outbreaks and cases reported, which was highest in Greece, with 1,363 outbreaks involving 1,535 canine cases, and lowest in North Macedonia, with only one outbreak and one canine case (Table 2).

**Table 2:** Number of outbreaks and cases of *Leishmania* infection in dogs and wildlife in Europe, reported to the World Organization for Animal Health (OIE-WAHIS) between 2005 and 2020.

	Outbreaks		Dogs		Wildlife	
Country	No. Years^a^	No.	No. Years^a^	No. cases	No. Years^a^	No. cases
Croatia	12	598	12	604	0	0
Greece	12	1,363	12	1,535	0	0
Italy	10	157	2	8	5	19
Montenegro	9	446	9	468	0	0
North Macedonia	1	1	1	1	0	0
Portugal	0	0	3	391	0	0
San Marino	1	11	1	21	0	0
Spain	9	614	12	1,155	1	1
All		3,190		4,183		20

^a^Number of years between 2005 and 2020 during which cases were reported.

## Discussion

In the period between 2005 and 2020, autochthonous cases of HumL were reported to the WHO-GHO in all European countries on the shores of the Mediterranean Sea, as well as in Bulgaria, North Macedonia, Romania, Serbia and Ukraine. The number of reported cases varied widely both spatially and temporarily, but there was no evidence of widespread increased incidence of HumL in the endemic European region. Visceral leishmaniasis accounted for almost 70% of HumL cases and the majority were considered autochthonous infections reported in decreasing order from Spain, Italy, Greece, France and Albania. Instead, incidence was highest in Albania followed by Montenegro, Malta, Greece, Spain and North Macedonia, highlighting the importance of assessing disease impact using both absolute and relative frequency measurements. In contrast to VL, most CL cases reported to the WHO were imported and notified in France. This may seem paradoxical given that leishmaniasis is not of mandatory notification in France [10], and also questions the efficacy of case notification systems in other Mediterranean European countries where HumL is a notifiable disease. Moreover, case notification may vary within countries, judging by the marked differences in HumL incidence in some close by regions. There was evidence for increasing incidence of CL in later years, which is in line with rising rates of migration from and travelling of Europeans to endemic countries [155,156]. This typically benign clinical form is often treated at primary health centers and private clinics and may indeed be extensively underreported [157]. Cutaneous leishmaniasis was more common than VL in a Community outbreak in Spain [5], and the estimated national incidence of both clinical forms in Italy and Croatia were considered to be similar by Alvar et al. [13]. There is a need to raise awareness among clinicians to improve CL diagnosis and notification. Visceral leishmaniasis was also underreported in the WHO data set, since the number of VL cases in Italy, Portugal and Spain was substantially lower than those in the hospital discharge databases. Moreover, the difference in the number of cases reported in these two data sources was not constant across years, thus limiting the accuracy of the temporal trend analysis of the WHO data carried out. The hospital discharge database is also likely to have some reporting bias; according to the Spanish Ministry of Health, the number of hospital discharges reported in 2020 are still provisional, and they caution of possible future updates in the number of cases in the 2017–2020 period. Bearing in mind these limitations, hospital discharge databases are potentially a great source of information for the analysis of the long-term temporal series of VL cases. Since areas with a high incidence of HumL are likely to also have a high incidence of leishmaniasis in dogs and other animals, human hospital discharge databases could also be very useful for the veterinary services to identify such areas. Animal leishmaniasis was clearly underreported to WOAH and case notification needs to be improved for a better understanding of the epidemiology and control of leishmaniasis. Some veterinary practitioners in high endemicity areas consider that the time required and the lack of financial compensation are discouraging factors for reporting cases (personal communication). There is evidence that sand fly vectors and CanL in Europe are slowly spreading to more northernly and higher altitude areas as a result of climate change making wider areas suitable for sand flies, and relocation of infected animal between regions [7,158–162]. The risk of CanL establishing local transmission of *L*. *infantum* in a non-endemic region with competent vectors, following the introduction of an infected dog was considered very high [163].

According to the Greek MoH data, incidence of VL in Greece increased significantly between 2009 and 2018, and incidence of VL and CL in Spain was significantly higher in 2016–2020 compared to 2012–2015. In contrast, VL incidence in Italy, Portugal and France was lower (marginally in France) in the latest time period compared to earlier ones. Also, WHO data reflects a significant decrease in VL incidence in Albania and an increase in imported CL cases in France during the study period. These results may seem contradictory to HumL being perceived as an emergent disease by health officials [10]. However, most Public Health authorities considered emergence a regional rather than a national problem, and this is supported by the observed differences in incidence between regions in Greece, Italy and Spain. Strengthening surveillance and case notification on a regional scale throughout Europe would be required for a more accurate assessment of the risk of leishmaniasis emergence.

There is no obvious explanation for the temporal variations in reported HumL incidence. Variability in disease risk can be attributed to changes in infection pressure and susceptibility of the resident population. Humans are considered accidental hosts of *L*. *infantum* and the risk of infection depends in most instances, on infection prevalence in synanthropic animal reservoir hosts, primarily dogs [164], and ultimately on the density and infection rates of local sand fly vector populations [165]. This information is not available in most countries and deserves better attention. *Leishmania infantum* control focuses on dogs, relying on preventing infections using mostly insect repellents and identifying and treating sick animals [166]. Croatia, Greece, Italy, Malta and Spain have national or regional leishmaniasis control programs with regular serological testing of dogs (mostly stray and shelter animals) and insecticide administration to dogs (Greece, Italy and Spain), and Albania performs indoor residual insecticide spraying [10]. Insecticides are also commonly used in household dogs in Western Europe, although manufacturer’s recommendations are seldom strictly followed in some countries, greatly reducing their efficacy to prevent infection [167]. Moreover, two vaccines against CanL have been commercialized in Europe (one has now been discontinued) but they do not prevent infection and their efficacy to prevent disease is partial [166]. These limitations and insufficient case reporting represent major obstacles for *L*. *infantum* control. Exceptionally, human infection has not been associated to CanL, as in the large community outbreak in Fuenlabrada in Madrid, where infection was associated to lagomorphs which behaved as an unusual primary reservoir of *L*. *infantum* [168,169]. The peak of this outbreak in 2011 [5], is reflected in the comparatively high incidence reported this year in Spain.

Hospital discharge databases provided useful information on patient’s gender and age and comorbidities and treatments in some cases. The percentage of males affected by HumL was higher compared to females in every country reporting this information. This has been noted before, the reasons are insufficiently understood, and it was attributed to gender-related behavioural and physiological differences affecting exposure and the immune response to infection [170]. Adults represented the majority of VL patients, and as previously reported in Spain, an increasing number of them were not HIV-coinfected but patients suffering from lymph/hematopoietic neoplasms and other immunodeficiencies as well as those having organ transplantation [6]. The switch in the age pattern of VL observed in most European countries in the last decades is less pronounced in Albania where children under 5 years old represented 47% of the annual VL cases in 2016 [171]. Paediatric leishmaniasis is traditionally associated with malnutrition and resource-poor communities, and incidence in children in Western European countries such as Spain, decreased during the second half of the twentieth century with the rise in living standards [172].

The review of the scientific literature revealed very few publications from countries with a relatively high incidence of HumL based on data reported to the WHO, such as North Macedonia, Montenegro and Malta. More studies need to be carried out in these and other Balkan countries to estimate the burden of leishmaniasis in animals and humans. On the other hand, countries where the literature reported sporadic infections only in animals and/or vectors, were those where HumL was either not reported or in low incidence in the WHO database. They include Germany, Austria, Hungary, Slovenia and Romania, which are situated on the perimeter of suitable vector habitats, and are priority areas to monitor for future northward spread of leishmaniasis [173]. The absence of HumL in these countries would partly explain the moderate spatial correlation between animal and human leishmaniasis. The other main reasons for this are case underreporting, and the geographical scale used for this analysis (NUTS-3 and GAUL-1 and 2 territorial subdivisions) which are not necessarily the level at which cases were reported, as was the case for HumL in Italy [12]. It would be highly desirable to elaborate fine-scale, ecologically-based maps, which combined with the information on human and animal case locations from this study and that on sand fly distributions available from the VectorNet project [174], should permit valuable risk assessments and the development of early warning systems to facilitate leishmaniasis control from a One Health perspective.

## Conclusions

Human and animal leishmaniasis caused by *L*. *infantum* is autochthonous in every country in the Mediterranean Biogeographical Region and the Balkan countries with highly variable incidence. Available information does not support widespread increased incidence of leishmaniasis across Europe, although underreporting is extensive, particularly of animal leishmaniasis and cutaneous human leishmaniasis. There is a clear need to encourage integrated surveillance and reporting. More emphasis is needed from an early start in medical and veterinary schools, on the importance of accurate reporting of leishmaniasis, as fundamental to defining effective control interventions. Human hospital discharge databases are now the most accurate source of data to estimate the incidence of human visceral leishmaniasis and should also be employed as an indirect measure to identify areas with a high incidence of animal leishmaniasis where control efforts should be upscaled.

## Supporting information

S1 Appendix PRISMA figure depicting the flow of documents obtained for each of the steps in the literature review in the database SCOPUS. (DOCX)

S1 Data Excel spreadsheet including, in separate sheets, all numerical values used for Figs 1, 2, 3, 4 and S1. (XLSX)

S1 Fig Regional distribution of autochthonous *Leishmania infantum* infections in humans and/or animals in European countries reported in the scientific literature and by the ministries of health of some countries, between 2009 and 2020. (TIF)

S1 Table Number and cumulative incidence per 100,000 population of cutaneous and visceral leishmaniasis in European countries between 2005 and 2020 as reported in the WHO-GHOD. (DOCX)

S2 Table Geographical references of reported autochthonous symptomatic and asymptomatic infections by *Leishmania infantum* in animals and/or humans in European countries, between 2009 and 2020. (DOCX)
