# Supplementary material for: The estimated distribution of autochthonous leishmaniasis by Leishmania infantum in Europe in 2005–2020
Source: PLoS Negl Trop Dis. 2023 Jul 19;17(7):e0011497. doi: 10.1371/journal.pntd.0011497 (PMC10389729; doi:10.1371/journal.pntd.0011497)
Supplement: S1 Appendix — (DOCX) [file pntd.0011497.s001.docx]

**S1 Appendix. PRISMA figure depicting the flow of documents obtained for each of the steps in the literature review in the database SCOPUS.**

Records identified through Scopus database searching N°2
(n = 650)

Records identified through Scopus database searching N°1
(n = 15,945)

Additional records:

- Thesis (n= 46)
- Articles (n= 33)
- Ministerial data (n= 5)
- WHO data (n= 29)
- Web pages (n=5)
- Computer programs (n=2)
- Reports (n=1)

## Identification

## Eligibility

## Included

## Screening

Records after duplicates removed
(n = 16,393)

Records excluded

(n = 14,531)

- Exclusion criteria^1^

- Out of scope^2^

- Other languages

- Other study areas

Title/abstract screened
(n= 1,862)

Full-text articles excluded
(n = 836)

- Non-endemic and outside the study zone
- Review articles with no relevant information

Full-text articles assessed for eligibility
(n = 1,026)

Documents included in the literature review
(n = 1,147)

Documents from Europe included in the literature review

(n = 695)

^1^Exclusion criteria: Studies of molecular or biochemical characterization of *Leishmania* spp., *in vitro* drug susceptibility, and *in vivo* laboratorial studies.

^2^Scope: Epidemiology, country surveillance activities, control, diagnostic methods, treatment, opinion on drivers for emergence and impact of leishmaniasis.
