# Supplementary figures and images for: The estimated distribution of autochthonous leishmaniasis by Leishmania infantum in Europe in 2005–2020
Source: PLoS Negl Trop Dis. 2023 Jul 19;17(7):e0011497. doi: 10.1371/journal.pntd.0011497 (PMC10389729; doi:10.1371/journal.pntd.0011497)

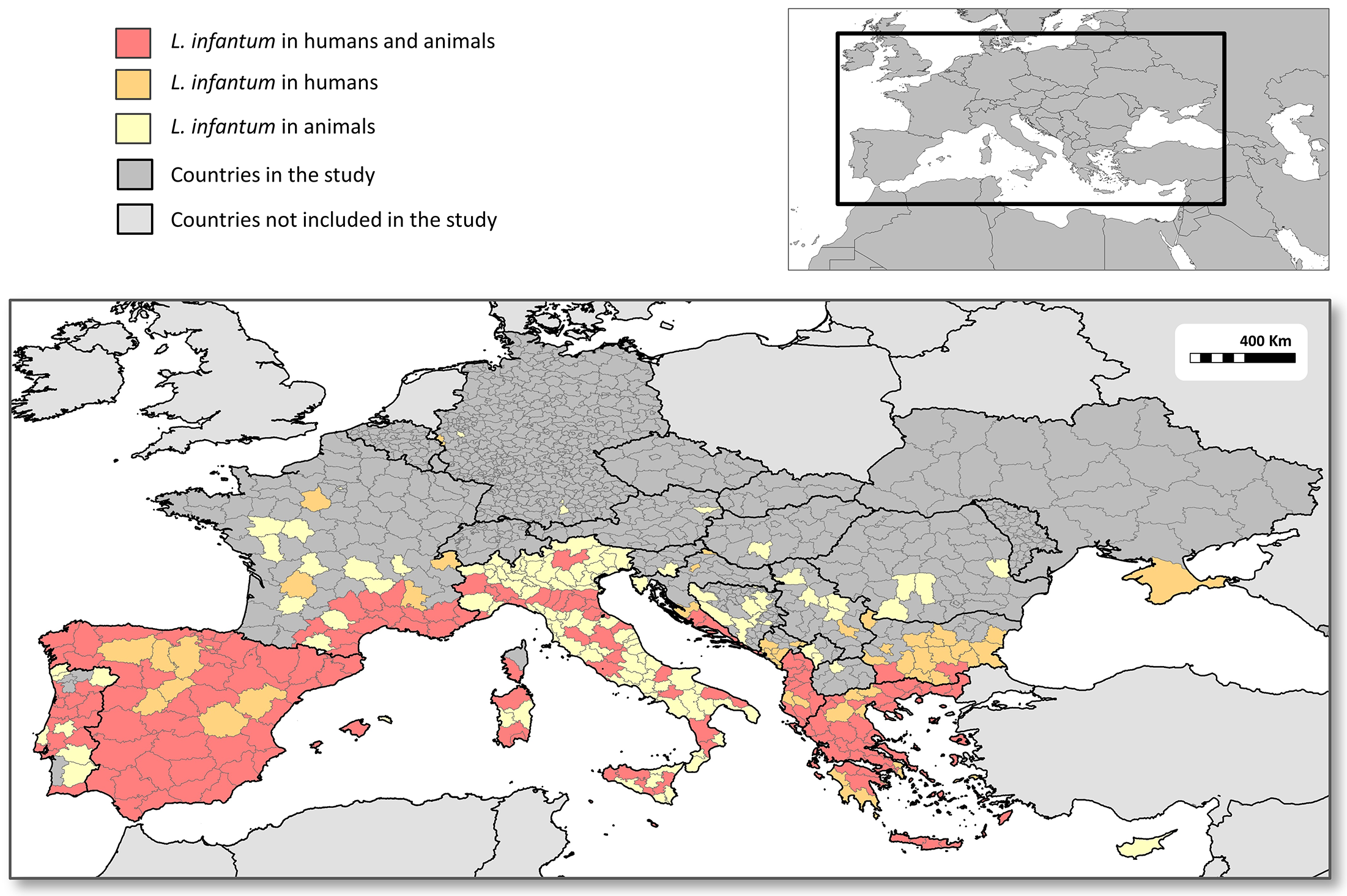

Supplement: S1 Fig — (TIF) [file pntd.0011497.s003.tif]
