# Supplementary material for: The estimated distribution of autochthonous leishmaniasis by Leishmania infantum in Europe in 2005–2020
Source: PLoS Negl Trop Dis. 2023 Jul 19;17(7):e0011497. doi: 10.1371/journal.pntd.0011497 (PMC10389729; doi:10.1371/journal.pntd.0011497)
Supplement: S1 Table — (DOCX) [file pntd.0011497.s004.docx]

**S1 Table. Number and cumulative incidence per 100,000 population of cutaneous and visceral leishmaniasis in European countries between 2005 and 2020 as reported in the WHO-GHOD.**

|  |  | **Cutaneous** | | **Visceral** | |  |  | **Cutaneous** | | **Visceral** | |
| --- | --- | --- | --- | --- | --- | --- | --- | --- | --- | --- | --- |
| **Country** | **Year** | **No. Cases** | **Incidence** | **No. Cases** | **Incidence** | **Country** | **Year** | **No. Cases** | **Incidence** | **No. Cases** | **Incidence** |
| **Albania** | **2005** | 3 | 0.0993 | 136 | 4.5039 | **Bosnia and**  **Herzegovina** | **2005** | 1 | 0.0266 | 1 | 0.0266 |
|  | **2006** | 3 | 0.0999 | 117 | 3.8957 |  | **2006** | NA | NA | 0 | 0.0000 |
|  | **2007** | 7 | 0.2348 | 108 | 3.6220 |  | **2007** | NA | NA | 2 | 0.0532 |
|  | **2008** | 2 | 0.0676 | 75 | 2.5353 |  | **2008** | NA | NA | 0 | 0.0000 |
|  | **2009** | 1 | 0.0341 | 62 | 2.1115 |  | **2009** | NA | NA | 1 | 0.0268 |
|  | **2010** | 1 | 0.0343 | 60 | 2.0557 |  | **2010** | NA | NA | 1 | 0.0270 |
|  | **2011** | 1 | 0.0344 | 52 | 1.7886 |  | **2011** | 0 | 0.0000 | 0 | 0.0000 |
|  | **2012** | 0 | 0.0000 | 53 | 1.8257 |  | **2012** | 0 | 0.0000 | 0 | 0.0000 |
|  | **2013** | 1 | 0.0345 | NA | NA |  | **2013** | 0 | 0.0000 | 0 | 0.0000 |
|  | **2014** | 0 | 0.0000 | NA | NA |  | **2014** | 0 | 0.0000 | 2 | 0.0574 |
|  | **2015** | 1 | 0.0347 | NA | NA |  | **2015** | 0 | 0.0000 | 0 | 0.0000 |
|  | **2016** | 6 | 0.2087 | 15 | 0.5216 |  | **2016** | 0 | 0.0000 | 0 | 0.0000 |
|  | **2017** | 0 | 0.0000 | 22 | 0.7648 |  | **2017** | 0 | 0.0000 | 0 | 0.0000 |
|  | **2018** | 2 | 0.0697 | 45 | 1.5678 |  | **2018** | NA | NA | NA | NA |
|  | **2019** | 2 | 0.0699 | 7 | 0.2445 |  | **2019** | NA | NA | NA | NA |
|  | **2020** | NA | NA | NA | NA |  | **2020** | NA | NA | NA | NA |
| **Bulgaria** | **2005** | NA | NA | 12 | 0.1561 | **Croatia** | **2005** | 2 | 0.0464 | 7 | 0.1624 |
|  | **2006** | NA | NA | 6 | 0.0786 |  | **2006** | 1 | 0.0232 | 6 | 0.1391 |
|  | **2007** | NA | NA | 6 | 0.0792 |  | **2007** | 5 | 0.1159 | 4 | 0.0927 |
|  | **2008** | NA | NA | 2 | 0.0266 |  | **2008** | 3 | 0.0696 | 4 | 0.0928 |
|  | **2009** | NA | NA | 6 | 0.0804 |  | **2009** | NA | NA | 1 | 0.0232 |
|  | **2010** | NA | NA | 4 | 0.0539 |  | **2010** | NA | NA | NA | NA |
|  | **2011** | NA | NA | 3 | 0.0407 |  | **2011** | 1 | 0.0233 | 0 | 0.0000 |
|  | **2012** | NA | NA | 2 | 0.0273 |  | **2012** | 2 | 0.0468 | 0 | 0.0000 |
|  | **2013** | 2 | 0.0275 | 14 | 0.1922 |  | **2013** | 5 | 0.1173 | 2 | 0.0469 |
|  | **2014** | 0 | 0.0000 | 14 | 0.1932 |  | **2014** | NA | NA | NA | NA |
|  | **2015** | 2 | 0.0278 | 5 | 0.0694 |  | **2015** | 4 | 0.0947 | 0 | 0.0000 |
|  | **2016** | 3 | 0.0419 | 3 | 0.0419 |  | **2016** | 2 | 0.0477 | 0 | 0.0000 |
|  | **2017** | NA | NA | 0 | 0.0000 |  | **2017** | NA | NA | NA | NA |
|  | **2018** | 3 | 0.0426 | 1 | 0.0142 |  | **2018** | 1 | 0.0244 | 0 | 0.0000 |
|  | **2019** | 0 | 0.0000 | 4 | 0.0571 |  | **2019** | 1 | 0.0245 | 0 | 0.0000 |
|  | **2020** | 1 | 0.0144 | 0 | 0.0000 |  | **2020** | NA | NA | NA | NA |
| **Cyprus** | **2005** | NA | NA | NA | NA | **France** | **2005** | 0 | 0.0000 | 19 | 0.0303 |
|  | **2006** | 4 | 0.5376 | 2 | 0.2688 |  | **2006** | 3 | 0.0047 | 16 | 0.0253 |
|  | **2007** | 0 | 0.0000 | 0 | 0.0000 |  | **2007** | 3 | 0.0047 | 22 | 0.0346 |
|  | **2008** | 0 | 0.0000 | 0 | 0.0000 |  | **2008** | 4 | 0.0062 | 17 | 0.0266 |
|  | **2009** | NA | NA | NA | NA |  | **2009** | 2 | 0.0031 | 14 | 0.0218 |
|  | **2010** | NA | NA | 1 | 0.1221 |  | **2010** | 8 | 0.0124 | 5 | 0.0077 |
|  | **2011** | NA | NA | 1 | 0.1191 |  | **2011** | 6 | 0.0092 | 25 | 0.0385 |
|  | **2012** | NA | NA | 0 | 0.0000 |  | **2012** | 5 | 0.0077 | 10 | 0.0153 |
|  | **2013** | 0 | 0.0000 | 0 | 0.0000 |  | **2013** | 93 | 0.1418 | 17 | 0.0259 |
|  | **2014** | NA | NA | NA | NA |  | **2014** | 84 | 0.1270 | 16 | 0.0242 |
|  | **2015** | 1 | 0.1181 | 0 | 0.0000 |  | **2015** | 110 | 0.1655 | 18 | 0.0271 |
|  | **2016** | 0 | 0.0000 | 1 | 0.1179 |  | **2016** | 77 | 0.1155 | 24 | 0.0360 |
|  | **2017** | 3 | 0.3510 | 1 | 0.1170 |  | **2017** | 132 | 0.1976 | 24 | 0.0359 |
|  | **2018** | 0 | 0.0000 | 2 | 0.2314 |  | **2018** | 181 | 0.2700 | 22 | 0.0328 |
|  | **2019** | 0 | 0.0000 | 0 | 0.0000 |  | **2019** | 182 | 0.2709 | 36 | 0.0536 |
|  | **2020** | 1 | 0.1126 | 0 | 0.0000 |  | **2020** | 204 | 0.3030 | 37 | 0.0550 |
| **Greece** | **2005** | 2 | 0.0182 | 48 | 0.4376 | **Hungary** | **2005** | NA | NA | NA | NA |
|  | **2006** | 0 | 0.0000 | 35 | 0.3180 |  | **2006** | NA | NA | NA | NA |
|  | **2007** | 6 | 0.0544 | 48 | 0.4349 |  | **2007** | NA | NA | NA | NA |
|  | **2008** | 4 | 0.0362 | 30 | 0.2712 |  | **2008** | NA | NA | NA | NA |
|  | **2009** | 7 | 0.0631 | 28 | 0.2524 |  | **2009** | NA | NA | NA | NA |
|  | **2010** | 2 | 0.0180 | 28 | 0.2518 |  | **2010** | NA | NA | NA | NA |
|  | **2011** | 2 | 0.0180 | 41 | 0.3686 |  | **2011** | NA | NA | NA | NA |
|  | **2012** | 3 | 0.0271 | 47 | 0.4239 |  | **2012** | NA | NA | NA | NA |
|  | **2013** | 1 | 0.0091 | 76 | 0.6907 |  | **2013** | 0 | 0.0000 | 0 | 0.0000 |
|  | **2014** | 1 | 0.0092 | 85 | 0.7779 |  | **2014** | NA | NA | NA | NA |
|  | **2015** | 4 | 0.0368 | 70 | 0.6447 |  | **2015** | NA | NA | NA | NA |
|  | **2016** | 14 | 0.1298 | 59 | 0.5471 |  | **2016** | NA | NA | NA | NA |
|  | **2017** | 2 | 0.0186 | 88 | 0.8172 |  | **2017** | NA | NA | NA | NA |
|  | **2018** | 1 | 0.0093 | 53 | 0.4934 |  | **2018** | NA | NA | NA | NA |
|  | **2019** | 1 | 0.0093 | 24 | 0.2238 |  | **2019** | NA | NA | NA | NA |
|  | **2020** | 0 | 0.0000 | 39 | 0.3639 |  | **2020** | NA | NA | NA | NA |
| **Italy** | **2005** | 73 | 0.1261 | 152 | 0.2626 | **Malta** | **2005** | 4 | 0.9934 | 4 | 0.9934 |
|  | **2006** | 36 | 0.0620 | 113 | 0.1946 |  | **2006** | 3 | 0.7407 | NA | NA |
|  | **2007** | 22 | 0.0378 | 104 | 0.1786 |  | **2007** | 13 | 3.2050 | NA | NA |
|  | **2008** | 23 | 0.0392 | 72 | 0.1228 |  | **2008** | 16 | 3.9232 | NA | NA |
|  | **2009** | 22 | 0.0373 | 72 | 0.1220 |  | **2009** | NA | NA | NA | NA |
|  | **2010** | 22 | 0.0372 | 71 | 0.1200 |  | **2010** | 11 | 2.6568 | NA | NA |
|  | **2011** | 24 | 0.0404 | 62 | 0.1044 |  | **2011** | 1 | 0.2410 | 2 | 0.4819 |
|  | **2012** | 26 | 0.0438 | 81 | 0.1364 |  | **2012** | 0 | 0.0000 | 3 | 0.7185 |
|  | **2013** | 96 | 0.1608 | 62 | 0.1039 |  | **2013** | 0 | 0.0000 | 0 | 0.0000 |
|  | **2014** | 80 | 0.1316 | 63 | 0.1036 |  | **2014** | NA | NA | NA | NA |
|  | **2015** | 76 | 0.1250 | 55 | 0.0905 |  | **2015** | NA | NA | NA | NA |
|  | **2016** | 47 | 0.0775 | 49 | 0.0808 |  | **2016** | 0 | 0.0000 | 0 | 0.0000 |
|  | **2017** | NA | NA | NA | NA |  | **2017** | 1 | 0.2173 | 5 | 1.0863 |
|  | **2018** | 71 | 0.1174 | 74 | 0.1223 |  | **2018** | 4 | 0.8409 | 2 | 0.4204 |
|  | **2019** | NA | NA | NA | NA |  | **2019** | 0 | 0.0000 | 0 | 0.0000 |
|  | **2020** | 6 | 0.0101 | 29 | 0.0486 |  | **2020** | 1 | 0.1943 | 2 | 0.3887 |
| **Montenegro** | **2005** | NA | NA | 2 | 0.3260 | **North**  **Macedonia** | **2005** | NA | NA | 7 | 0.3439 |
|  | **2006** | NA | NA | 4 | 0.6524 |  | **2006** | NA | NA | 9 | 0.4415 |
|  | **2007** | NA | NA | 1 | 0.1627 |  | **2007** | NA | NA | 7 | 0.3428 |
|  | **2008** | NA | NA | 3 | 0.4874 |  | **2008** | NA | NA | 7 | 0.3423 |
|  | **2009** | NA | NA | 3 | 0.4861 |  | **2009** | NA | NA | 4 | 0.1953 |
|  | **2010** | NA | NA | 1 | 0.1616 |  | **2010** | NA | NA | 12 | 0.5846 |
|  | **2011** | NA | NA | NA | NA |  | **2011** | NA | NA | 2 | 0.0972 |
|  | **2012** | NA | NA | NA | NA |  | **2012** | NA | NA | 13 | 0.6311 |
|  | **2013** | NA | NA | 4 | 0.6442 |  | **2013** | NA | NA | 20 | 0.9698 |
|  | **2014** | NA | NA | 3 | 0.4827 |  | **2014** | NA | NA | 11 | 0.5325 |
|  | **2015** | 0 | 0.0000 | 5 | 0.8037 |  | **2015** | 0 | 0.0000 | 4 | 0.1933 |
|  | **2016** | 0 | 0.0000 | 6 | 0.9643 |  | **2016** | 0 | 0.0000 | 5 | 0.2414 |
|  | **2017** | 0 | 0.0000 | 4 | 0.6427 |  | **2017** | 0 | 0.0000 | 9 | 0.4340 |
|  | **2018** | NA | NA | NA | NA |  | **2018** | 0 | 0.0000 | 12 | 0.5782 |
|  | **2019** | NA | NA | NA | NA |  | **2019** | NA | NA | NA | NA |
|  | **2020** | NA | NA | NA | NA |  | **2020** | NA | NA | NA | NA |
| **Spain** | **2005** | 16 | 0.0370 | 199 | 0.4596 | **Ukraine** | **2005** | 4 | 0.0085 | 1 | 0.0021 |
|  | **2006** | 6 | 0.0136 | 246 | 0.5590 |  | **2006** | 2 | 0.0043 | 3 | 0.0064 |
|  | **2007** | 6 | 0.0134 | 255 | 0.5694 |  | **2007** | 1 | 0.0022 | 3 | 0.0065 |
|  | **2008** | 16 | 0.0350 | 193 | 0.4226 |  | **2008** | 3 | 0.0065 | 2 | 0.0043 |
|  | **2009** | 10 | 0.0216 | 179 | 0.3871 |  | **2009** | NA | NA | NA | NA |
|  | **2010** | 10 | 0.0215 | 153 | 0.3291 |  | **2010** | 0 | 0.0000 | 1 | 0.0022 |
|  | **2011** | 10 | 0.0214 | 235 | 0.5036 |  | **2011** | 2 | 0.0044 | 1 | 0.0022 |
|  | **2012** | 18 | 0.0384 | 213 | 0.4550 |  | **2012** | 2 | 0.0044 | 2 | 0.0044 |
|  | **2013** | NA | NA | 276 | 0.5907 |  | **2013** | 2 | 0.0044 | 0 | 0.0000 |
|  | **2014** | 100 | 0.2150 | 106 | 0.2279 |  | **2014** | 1 | 0.0022 | 0 | 0.0000 |
|  | **2015** | 40 | 0.0861 | NA | NA |  | **2015** | 2 | 0.0044 | 0 | 0.0000 |
|  | **2016** | 187 | 0.4027 | 177 | 0.3811 |  | **2016** | 4 | 0.0089 | 1 | 0.0022 |
|  | **2017** | 185 | 0.3976 | 199 | 0.4277 |  | **2017** | 1 | 0.0022 | 0 | 0.0000 |
|  | **2018** | NA | NA | NA | NA |  | **2018** | NA | NA | NA | NA |
|  | **2019** | NA | NA | NA | NA |  | **2019** | 2 | 0.0045 | 0 | 0.0000 |
|  | **2020** | NA | NA | NA | NA |  | **2020** | 0 | 0.0000 | 0 | 0.0000 |
| **Europe** | **2005** | 105 | 0.0367 | 601 | 0.2102 | **Europe** | **2013** | 201 | 0.0693 | 481 | 0.1657 |
|  | **2006** | 58 | 0.0202 | 567 | 0.1977 |  | **2014** | 275 | 0.0945 | 300 | 0.1031 |
|  | **2007** | 63 | 0.0219 | 583 | 0.2026 |  | **2015** | 249 | 0.0856 | 158 | 0.0543 |
|  | **2008** | 71 | 0.0246 | 419 | 0.1452 |  | **2016** | 341 | 0.1174 | 351 | 0.1209 |
|  | **2009** | 42 | 0.0145 | 381 | 0.1316 |  | **2017** | 325 | 0.1120 | 355 | 0.1224 |
|  | **2010** | 54 | 0.0186 | 354 | 0.1222 |  | **2018** | 263 | 0.0907 | 217 | 0.0749 |
|  | **2011** | 49 | 0.0169 | 439 | 0.1513 |  | **2019** | 189 | 0.0654 | 71 | 0.0246 |
|  | **2012** | 57 | 0.0196 | 430 | 0.1482 |  | **2020** | 214 | 0.0740 | 107 | 0.0370 |

NA: Data not available.
