# Supplementary material for: The estimated distribution of autochthonous leishmaniasis by Leishmania infantum in Europe in 2005–2020
Source: PLoS Negl Trop Dis. 2023 Jul 19;17(7):e0011497. doi: 10.1371/journal.pntd.0011497 (PMC10389729; doi:10.1371/journal.pntd.0011497)
Supplement: S2 Table — (DOCX) [file pntd.0011497.s005.docx]

**S2 Table. Geographical references of reported autochthonous symptomatic and asymptomatic infections by *Leishmania infantum* in animals and/or humans in European countries, between 2009 and 2020.**

| **Country** | **Division** | **Location** | | | | **Infections** | | **References** |
| --- | --- | --- | --- | --- | --- | --- | --- | --- |
|  |  | **Code** | **Name** | **Latitude** | **Longitude** | **Animals** | **Humans** |  |
| Albania | NUTS-3 | AL011 | Dibër | 41.607 | 20.247 | Yes | Yes | [1,2] |
|  | NUTS-3 | AL012 | Durrës | 41.451 | 19.639 | Yes | Yes | [1,2] |
|  | NUTS-3 | AL013 | Kukës | 42.195 | 20.243 | Yes | Yes | [1,3,4] |
|  | NUTS-3 | AL014 | Lezhë | 41.790 | 19.838 | Yes | Yes | [1,2] |
|  | NUTS-3 | AL015 | Shkodër | 42.174 | 19.720 | Yes | Yes | [1,4] |
|  | NUTS-3 | AL021 | Elbasan | 41.054 | 20.191 | Yes | Yes | [1,2] |
|  | NUTS-3 | AL022 | Tiranë | 41.266 | 19.796 | Yes | Yes | [1,2,5–7] |
|  | NUTS-3 | AL031 | Berat | 40.626 | 20.115 | Yes | Yes | [1,4,8] |
|  | NUTS-3 | AL032 | Fier | 40.759 | 19.623 | No | Yes | [1] |
|  | NUTS-3 | AL033 | Gjirokastër | 40.216 | 20.170 | No | Yes | [1] |
|  | NUTS-3 | AL034 | Korcë | 40.629 | 20.670 | Yes | Yes | [1–3,8] |
|  | NUTS-3 | AL035 | Vlorë | 40.149 | 19.818 | Yes | Yes | [1,2,4,8] |
| Austria | NUTS-3 | AT127 | Wiener Umland/Südteil | 48.052 | 16.445 | Yes | No | [9] |
| Bosnia and Herzegovina | GAUL-2 | BAG26241 | Gornjedrinski | 43.667 | 18.818 | Yes | No | [10] |
|  | GAUL-2 | BAG26242 | Neretvljanski | 43.409 | 17.841 | Yes | No | [10,11] |
|  | GAUL-2 | BAG26243 | Posavski | 45.051 | 18.491 | Yes | No | [10] |
|  | GAUL-2 | BAG26244 | Sarajevo | 43.814 | 18.340 | Yes | No | [10] |
|  | GAUL-2 | BAG26246 | Tuzlansko-podrinjski | 44.569 | 18.597 | Yes | No | [10] |
|  | GAUL-2 | BAG26247 | Unsko-sanski | 44.791 | 16.244 | Yes | No | [10] |
|  | GAUL-2 | BAG26248 | Zapadnobosanski | 44.008 | 16.841 | Yes | No | [10] |
|  | GAUL-2 | BAG26250 | Zenicko-dobojski | 44.290 | 18.181 | Yes | No | [10] |
| Bulgaria | NUTS-3 | BG311 | Vidin | 43.798 | 22.701 | No | Yes | [12], MoH Bulgaria^1^ |
|  | NUTS-3 | BG315 | Lovech | 43.026 | 24.554 | No | Yes | [12] |
|  | NUTS-3 | BG321 | Veliko Tarnovo | 43.190 | 25.628 | No | Yes | [12], MoH Bulgaria^1^ |
|  | NUTS-3 | BG322 | Gabrovo | 42.937 | 25.227 | No | Yes | MoH Bulgaria^1^ |
|  | NUTS-3 | BG331 | Varna | 43.178 | 27.584 | No | Yes | [12] |
|  | NUTS-3 | BG341 | Burgas | 42.491 | 27.308 | No | Yes | MoH Bulgaria^1^ |
|  | NUTS-3 | BG342 | Sliven | 42.680 | 26.238 | No | Yes | MoH Bulgaria^1^ |
|  | NUTS-3 | BG343 | Yambol | 42.291 | 26.627 | No | Yes | [12] |
|  | NUTS-3 | BG344 | Stara Zagora | 42.430 | 25.557 | No | Yes | [12] |
|  | NUTS-3 | BG411 | Sofia (stolitsa) | 42.678 | 23.368 | No | Yes | [12], MoH Bulgaria^1^ |
|  | NUTS-3 | BG413 | Blagoevgrad | 41.723 | 23.439 | Yes | Yes | [12], MoH Bulgaria^1^ |
|  | NUTS-3 | BG415 | Kyustendil | 42.270 | 22.862 | No | Yes | [12] |
|  | NUTS-3 | BG421 | Plovdiv | 42.265 | 24.819 | No | Yes | [12], MoH Bulgaria^1^ |
|  | NUTS-3 | BG422 | Haskovo | 41.852 | 25.924 | Yes | Yes | [12] |
|  | NUTS-3 | BG423 | Pazardzhik | 42.137 | 24.153 | No | Yes | [12] |
|  | NUTS-3 | BG424 | Smolyan | 41.629 | 24.668 | No | Yes | MoH Bulgaria^1^ |
|  | NUTS-3 | BG425 | Kardzhali | 41.533 | 25.437 | No | Yes | [12], MoH Bulgaria^1^ |
| Croatia | NUTS-3 | HR033 | Zadarska županija | 44.187 | 15.602 | No | Yes | [13] |
|  | NUTS-3 | HR034 | Šibensko-kninska županija | 43.893 | 16.056 | Yes | Yes | [13,14] |
|  | NUTS-3 | HR035 | Splitsko-dalmatinska županija | 43.481 | 16.717 | Yes | Yes | [5,13–16] |
|  | NUTS-3 | HR036 | Istarska županija | 45.201 | 13.897 | Yes | No | [17] |
|  | NUTS-3 | HR037 | Dubrovacko-neretvanska županija | 42.880 | 17.554 | Yes | Yes | [5,13,16,18] |
|  | NUTS-3 | HR050 | Grad Zagreb | 45.828 | 16.022 | No | Yes | [19] |
|  | NUTS-3 | HR061 | Međimurska županija | 46.404 | 16.531 | No | Yes | [16] |
| Cyprus | NUTS-3 | CY000 | Kýpros | 35.051 | 33.225 | Yes | No | [5,20–23] |
| France | NUTS-3 | FR101 | Paris | 48.856 | 2.338 | Yes | No | [24] |
|  | NUTS-3 | FRB02 | Eure-et-Loir | 48.376 | 1.366 | No | Yes | [25] |
|  | NUTS-3 | FRB04 | Indre-et-Loire | 47.258 | 0.682 | Yes | No | [26] |
|  | NUTS-3 | FRG02 | Maine-et-Loire | 47.389 | -0.561 | Yes | No | [26] |
|  | NUTS-3 | FRI11 | Dordogne | 45.104 | 0.740 | No | Yes | [27] |
|  | NUTS-3 | FRI14 | Lot-et-Garonne | 44.363 | 0.459 | Yes | No | [26] |
|  | NUTS-3 | FRI23 | Haute-Vienne | 45.886 | 1.228 | Yes | No | [26] |
|  | NUTS-3 | FRI33 | Deux-Sèvres | 46.553 | -0.318 | Yes | No | [26] |
|  | NUTS-3 | FRJ11 | Aude | 43.104 | 2.422 | Yes | Yes | [26,28] |
|  | NUTS-3 | FRJ12 | Gard | 43.995 | 4.175 | Yes | Yes | [26,28] |
|  | NUTS-3 | FRJ13 | Hérault | 43.576 | 3.366 | Yes | Yes | [26,28,29] |
|  | NUTS-3 | FRJ14 | Lozère | 44.523 | 3.494 | Yes | Yes | [26,28] |
|  | NUTS-3 | FRJ15 | Pyrénées-Orientales | 42.598 | 2.529 | Yes | Yes | [26,28] |
|  | NUTS-3 | FRJ21 | Ariège | 42.920 | 1.515 | Yes | No | [26] |
|  | NUTS-3 | FRJ22 | Aveyron | 44.277 | 2.684 | Yes | Yes | [26,28] |
|  | NUTS-3 | FRJ23 | Haute-Garonne | 43.355 | 1.165 | Yes | Yes | [30,31] |
|  | NUTS-3 | FRJ27 | Tarn | 43.785 | 2.168 | Yes | No | [26] |
|  | NUTS-3 | FRK13 | Haute-Loire | 45.126 | 3.807 | Yes | No | [26] |
|  | NUTS-3 | FRK14 | Puy-de-Dôme | 45.726 | 3.136 | Yes | No | [26] |
|  | NUTS-3 | FRK22 | Ardèche | 44.756 | 4.430 | Yes | Yes | [26,28] |
|  | NUTS-3 | FRK23 | Drôme | 44.682 | 5.168 | No | Yes | [28] |
|  | NUTS-3 | FRK26 | Rhône | 45.875 | 4.647 | Yes | No | [32] |
|  | NUTS-3 | FRK28 | Haute-Savoie | 46.053 | 6.434 | No | Yes | [33] |
|  | NUTS-3 | FRL01 | Alpes-de-Haute-Provence | 44.094 | 6.247 | Yes | Yes | [26,28] |
|  | NUTS-3 | FRL03 | Alpes-Maritimes | 43.929 | 7.120 | Yes | Yes | [26,28,34–36] |
|  | NUTS-3 | FRL04 | Bouches-du-Rhône | 43.545 | 5.104 | Yes | Yes | [26,28,37–39] |
|  | NUTS-3 | FRL05 | Var | 43.437 | 6.252 | Yes | Yes | [26,28,37,40,41] |
|  | NUTS-3 | FRL06 | Vaucluse | 44.012 | 5.164 | Yes | Yes | [26,28] |
|  | NUTS-3 | FRM01 | Corse-du-Sud | 41.864 | 8.993 | Yes | Yes | [26,28,37] |
| Germany | NUTS-3 | DE21E | Landsberg am Lech | 48.037 | 10.951 | Yes | No | [42] |
|  | NUTS-3 | DE271 | Augsburg, Kreisfreie Stadt | 48.350 | 10.889 | Yes | No | [42] |
|  | NUTS-3 | DEA23 | Köln, Kreisfreie Stadt | 50.960 | 6.965 | Yes | No | [42] |
|  | NUTS-3 | DEA2D | Städteregion Aachen | 50.729 | 6.218 | No | Yes | [42] |
| Greece | NUTS-3 | EL301 | Voreios Tomeas Athinon | 38.046 | 23.814 | Yes | Yes | [43–50], MoH Greece^2^ |
|  | NUTS-3 | EL302 | Dytikos Tomeas Athinon | 38.015 | 23.663 | Yes | Yes | [46,50], MoH Greece^2^ |
|  | NUTS-3 | EL303 | Kentrikos Tomeas Athinon | 37.982 | 23.757 | Yes | Yes | [5,46,50–58], MoH Greece^2^ |
|  | NUTS-3 | EL304 | Notios Tomeas Athinon | 37.914 | 23.739 | Yes | Yes | [46,50], MoH Greece^2^ |
|  | NUTS-3 | EL305 | Anatoliki Attiki | 38.024 | 23.886 | No | Yes | [46], MoH Greece^2^ |
|  | NUTS-3 | EL306 | Dytiki Attiki | 38.095 | 23.392 | Yes | Yes | [46,50,59], MoH Greece^2^ |
|  | NUTS-3 | EL307 | Peiraias, Nisoi | 37.129 | 23.374 | No | Yes | [46], MoH Greece^2^ |
|  | NUTS-3 | EL411 | Lesvos, Limnos | 39.338 | 25.998 | Yes | Yes | [5,46,50,60], MoH Greece^2^ |
|  | NUTS-3 | EL412 | Ikaria, Samos | 37.672 | 26.526 | No | Yes | [46], MoH Greece^2^ |
|  | NUTS-3 | EL413 | Chios | 38.412 | 25.983 | Yes | Yes | [46,50], MoH Greece^2^ |
|  | NUTS-3 | EL421 | Kalymnos, Karpathos, Kasos, Kos, Rodos | 36.326 | 27.497 | Yes | Yes | [46,50], MoH Greece^2^ |
|  | NUTS-3 | EL422 | Andros, Thira, Kea, Milos, Mykonos, Naxos, Paros, Syros, Tinos | 37.134 | 25.097 | Yes | Yes | [44,46,50], MoH Greece^2^ |
|  | NUTS-3 | EL431 | Irakleio | 35.140 | 25.125 | Yes | Yes | [5,46,50,61], MoH Greece^2^ |
|  | NUTS-3 | EL432 | Lasithi | 35.129 | 25.790 | Yes | Yes | [5,46,50], MoH Greece^2^ |
|  | NUTS-3 | EL433 | Rethymni | 35.259 | 24.618 | Yes | Yes | [5,44,46,50,52,61–63], MoH Greece^2^ |
|  | NUTS-3 | EL434 | Chania | 35.360 | 23.932 | Yes | Yes | [46,50,61], MoH Greece^2^ |
|  | NUTS-3 | EL511 | Evros | 41.194 | 26.152 | Yes | Yes | [43,44,46,50] |
|  | NUTS-3 | EL512 | Xanthi | 41.159 | 24.904 | Yes | Yes | [46,50,64,65], MoH Greece^2^ |
|  | NUTS-3 | EL513 | Rodopi | 41.107 | 25.503 | Yes | Yes | [46,50], MoH Greece^2^ |
|  | NUTS-3 | EL514 | Drama | 41.298 | 24.200 | Yes | Yes | [5,46,50], MoH Greece^2^ |
|  | NUTS-3 | EL515 | Thasos, Kavala | 40.918 | 24.447 | Yes | Yes | [5,46,50], MoH Greece^2^ |
|  | NUTS-3 | EL521 | Imathia | 40.548 | 22.239 | Yes | Yes | [5,46,50], MoH Greece^2^ |
|  | NUTS-3 | EL522 | Thessaloniki | 40.693 | 23.128 | Yes | Yes | [5,44,46,50,60,66–71], MoH Greece^2^ |
|  | NUTS-3 | EL523 | Kilkis | 41.030 | 22.752 | No | Yes | [46], MoH Greece^2^ |
|  | NUTS-3 | EL524 | Pella | 40.883 | 22.131 | No | Yes | [46] |
|  | NUTS-3 | EL525 | Pieria | 40.252 | 22.437 | No | Yes | [46], MoH Greece^2^ |
|  | NUTS-3 | EL526 | Serres | 41.089 | 23.523 | Yes | Yes | [43,44,46,50,60], MoH Greece^2^ |
|  | NUTS-3 | EL527 | Chalkidiki | 40.317 | 23.567 | Yes | Yes | [46,50,60,71], MoH Greece^2^ |
|  | NUTS-3 | EL531 | Grevena, Kozani | 40.206 | 21.597 | No | Yes | [46], MoH Greece^2^ |
|  | NUTS-3 | EL532 | Kastoria | 40.463 | 21.154 | Yes | Yes | [46,60] |
|  | NUTS-3 | EL533 | Florina | 40.756 | 21.430 | Yes | Yes | [43,44,46,50], MoH Greece^2^ |
|  | NUTS-3 | EL541 | Arta, Preveza | 39.245 | 20.968 | Yes | Yes | [5,46,50], MoH Greece^2^ |
|  | NUTS-3 | EL542 | Thesprotia | 39.523 | 20.405 | Yes | Yes | [46,50], MoH Greece^2^ |
|  | NUTS-3 | EL543 | Ioannina | 39.810 | 20.807 | Yes | Yes | [5,43,44,46,50,72], MoH Greece^2^ |
|  | NUTS-3 | EL611 | Karditsa, Trikala | 39.502 | 21.717 | Yes | Yes | [5,44,46,50,73–77], MoH Greece^2^ |
|  | NUTS-3 | EL612 | Larisa | 39.703 | 22.399 | Yes | Yes | [46,54,74,78], MoH Greece^2^ |
|  | NUTS-3 | EL613 | Magnisia, Sporades | 39.266 | 23.010 | Yes | Yes | [46,50,74,75,79], MoH Greece^2^ |
|  | NUTS-3 | EL621 | Zakynthos | 37.793 | 20.765 | Yes | Yes | [46,61], MoH Greece^2^ |
|  | NUTS-3 | EL622 | Kerkyra | 39.648 | 19.821 | Yes | Yes | [5,46,50,61], MoH Greece^2^ |
|  | NUTS-3 | EL623 | Ithaki, Kefallinia | 38.239 | 20.586 | Yes | Yes | [46,50], MoH Greece^2^ |
|  | NUTS-3 | EL624 | Lefkada | 38.690 | 20.705 | Yes | Yes | [46,50], MoH Greece^2^ |
|  | NUTS-3 | EL631 | Aitoloakarnania | 38.683 | 21.393 | Yes | Yes | [46,50], MoH Greece^2^ |
|  | NUTS-3 | EL632 | Achaia | 38.057 | 21.898 | Yes | Yes | [5,46,50], MoH Greece^2^ |
|  | NUTS-3 | EL633 | Ileia | 37.737 | 21.563 | No | Yes | [46], MoH Greece^2^ |
|  | NUTS-3 | EL641 | Voiotia | 38.371 | 23.080 | Yes | Yes | [46,50], MoH Greece^2^ |
|  | NUTS-3 | EL642 | Evvoia | 38.548 | 23.805 | Yes | Yes | [5,43,44,46,50], MoH Greece^2^ |
|  | NUTS-3 | EL643 | Evrytania | 38.984 | 21.671 | Yes | Yes | [43,44,46] |
|  | NUTS-3 | EL644 | Fthiotida | 38.860 | 22.507 | Yes | Yes | [5,46,50,80], MoH Greece^2^ |
|  | NUTS-3 | EL645 | Fokida | 38.541 | 22.250 | Yes | Yes | [46,50], MoH Greece^2^ |
|  | NUTS-3 | EL651 | Argolida, Arkadia | 37.521 | 22.510 | Yes | Yes | [5,44,46,50], MoH Greece^2^ |
|  | NUTS-3 | EL652 | Korinthia | 37.919 | 22.711 | Yes | Yes | [46,50], MoH Greece^2^ |
|  | NUTS-3 | EL653 | Lakonia, Messinia | 37.003 | 22.313 | No | Yes | [46], MoH Greece^2^ |
| Hungary | NUTS-3 | HU233 | Tolna | 46.512 | 18.538 | Yes | No | [81] |
| Italy | NUTS-3 | ITC11 | Torino | 45.145 | 7.449 | Yes | Yes | [82,83] |
|  | NUTS-3 | ITC12 | Vercelli | 45.515 | 8.224 | Yes | No | [83] |
|  | NUTS-3 | ITC13 | Biella | 45.584 | 8.088 | Yes | No | [83] |
|  | NUTS-3 | ITC14 | Verbano-Cusio-Ossola | 46.085 | 8.348 | Yes | No | [83] |
|  | NUTS-3 | ITC15 | Novara | 45.565 | 8.559 | Yes | No | [83] |
|  | NUTS-3 | ITC16 | Cuneo | 44.470 | 7.588 | Yes | No | [83] |
|  | NUTS-3 | ITC17 | Asti | 44.871 | 8.197 | Yes | Yes | [82–84] |
|  | NUTS-3 | ITC18 | Alessandria | 44.829 | 8.661 | Yes | Yes | [82,83] |
|  | NUTS-3 | ITC20 | Valle d’Aosta/Vallée d’Aoste | 45.734 | 7.400 | Yes | No | [83,85] |
|  | NUTS-3 | ITC31 | Imperia | 43.952 | 7.809 | Yes | Yes | [36,83,86,87] |
|  | NUTS-3 | ITC32 | Savona | 44.285 | 8.299 | Yes | No | [83] |
|  | NUTS-3 | ITC33 | Genova | 44.467 | 9.112 | Yes | Yes | [83,88] |
|  | NUTS-3 | ITC34 | La Spezia | 44.224 | 9.722 | Yes | No | [83] |
|  | NUTS-3 | ITC41 | Varese | 45.801 | 8.782 | Yes | No | [83,85] |
|  | NUTS-3 | ITC42 | Como | 45.929 | 9.158 | Yes | No | [83] |
|  | NUTS-3 | ITC43 | Lecco | 45.888 | 9.396 | Yes | No | [83] |
|  | NUTS-3 | ITC44 | Sondrio | 46.293 | 9.907 | Yes | No | [83] |
|  | NUTS-3 | ITC46 | Bergamo | 45.792 | 9.788 | Yes | No | [83] |
|  | NUTS-3 | ITC47 | Brescia | 45.698 | 10.316 | Yes | No | [83,85] |
|  | NUTS-3 | ITC48 | Pavia | 45.100 | 9.043 | Yes | No | [83] |
|  | NUTS-3 | ITC49 | Lodi | 45.226 | 9.586 | Yes | No | [83,89] |
|  | NUTS-3 | ITC4A | Cremona | 45.222 | 9.989 | Yes | No | [83] |
|  | NUTS-3 | ITC4B | Mantova | 45.121 | 10.760 | Yes | No | [83,85] |
|  | NUTS-3 | ITC4C | Milano | 45.462 | 9.130 | Yes | No | [83,90–95] |
|  | NUTS-3 | ITC4D | Monza e della Brianza | 45.636 | 9.262 | Yes | No | [83] |
|  | NUTS-3 | ITF11 | L’Aquila | 42.108 | 13.619 | Yes | No | [83] |
|  | NUTS-3 | ITF12 | Teramo | 42.649 | 13.747 | Yes | No | [83,96–98] |
|  | NUTS-3 | ITF13 | Pescara | 42.357 | 14.011 | Yes | No | [83,85] |
|  | NUTS-3 | ITF14 | Chieti | 42.094 | 14.398 | Yes | No | [83,85] |
|  | NUTS-3 | ITF21 | Isernia | 41.643 | 14.237 | Yes | No | [83] |
|  | NUTS-3 | ITF22 | Campobasso | 41.709 | 14.773 | Yes | Yes | [83,99,100] |
|  | NUTS-3 | ITF31 | Caserta | 41.211 | 14.153 | Yes | No | [83,101] |
|  | NUTS-3 | ITF32 | Benevento | 41.258 | 14.752 | Yes | No | [83] |
|  | NUTS-3 | ITF33 | Napoli | 40.873 | 14.389 | Yes | No | [83,85,102–108] |
|  | NUTS-3 | ITF34 | Avellino | 40.996 | 15.066 | Yes | Yes | [83,109] |
|  | NUTS-3 | ITF35 | Salerno | 40.462 | 15.210 | Yes | No | [83,110] |
|  | NUTS-3 | ITF43 | Taranto | 40.586 | 17.262 | Yes | No | [83,85] |
|  | NUTS-3 | ITF44 | Brindisi | 40.618 | 17.715 | Yes | Yes | [83,111,112] |
|  | NUTS-3 | ITF45 | Lecce | 40.200 | 18.138 | Yes | No | [83,85] |
|  | NUTS-3 | ITF46 | Foggia | 41.538 | 15.545 | Yes | No | [83] |
|  | NUTS-3 | ITF47 | Bari | 40.927 | 16.754 | Yes | Yes | [83,85,113–117] |
|  | NUTS-3 | ITF48 | Barletta-Andria-Trani | 41.182 | 16.179 | Yes | No | [83,85] |
|  | NUTS-3 | ITF51 | Potenza | 40.543 | 15.872 | Yes | No | [83,118] |
|  | NUTS-3 | ITF52 | Matera | 40.466 | 16.474 | Yes | No | [83,119] |
|  | NUTS-3 | ITF61 | Cosenza | 39.560 | 16.318 | Yes | Yes | [83,85,100,120] |
|  | NUTS-3 | ITF62 | Crotone | 39.191 | 16.921 | Yes | No | [83] |
|  | NUTS-3 | ITF63 | Catanzaro | 38.892 | 16.487 | Yes | Yes | [83,121,122] |
|  | NUTS-3 | ITF64 | Vibo Valentia | 38.617 | 16.197 | Yes | No | [83] |
|  | NUTS-3 | ITF65 | Reggio di Calabria | 38.280 | 16.043 | Yes | No | [83] |
|  | NUTS-3 | ITG11 | Trapani | 37.814 | 12.793 | Yes | Yes | [83,123] |
|  | NUTS-3 | ITG12 | Palermo | 37.887 | 13.593 | Yes | Yes | [83,124–129] |
|  | NUTS-3 | ITG13 | Messina | 38.015 | 15.053 | Yes | No | [83,85,124,128–136] |
|  | NUTS-3 | ITG14 | Agrigento | 37.463 | 13.488 | Yes | No | [83,137] |
|  | NUTS-3 | ITG15 | Caltanissetta | 37.188 | 14.270 | Yes | Yes | [83,138] |
|  | NUTS-3 | ITG16 | Enna | 37.615 | 14.454 | Yes | No | [83,124] |
|  | NUTS-3 | ITG17 | Catania | 37.593 | 15.032 | Yes | Yes | [83,85,130,139,140] |
|  | NUTS-3 | ITG18 | Ragusa | 36.919 | 14.670 | Yes | No | [83,124] |
|  | NUTS-3 | ITG19 | Siracusa | 37.059 | 15.031 | Yes | No | [83,130,131] |
|  | NUTS-3 | ITG2D | Sassari | 40.610 | 8.708 | Yes | Yes | [83,141,142] |
|  | NUTS-3 | ITG2E | Nuoro | 40.397 | 9.474 | Yes | No | [83] |
|  | NUTS-3 | ITG2F | Cagliari | 39.416 | 9.187 | Yes | Yes | [83,85,143–145] |
|  | NUTS-3 | ITG2G | Oristano | 40.001 | 8.730 | Yes | No | [83] |
|  | NUTS-3 | ITG2H | Sud Sardegna | 39.506 | 8.698 | Yes | No | [83] |
|  | NUTS-3 | ITH10 | Bolzano-Bozen | 46.705 | 11.424 | Yes | No | [83,85] |
|  | NUTS-3 | ITH20 | Trento | 46.131 | 10.859 | Yes | Yes | [83,100] |
|  | NUTS-3 | ITH31 | Verona | 45.419 | 11.048 | Yes | No | [83,85] |
|  | NUTS-3 | ITH32 | Vicenza | 45.656 | 11.506 | Yes | No | [83,85] |
|  | NUTS-3 | ITH33 | Belluno | 46.313 | 12.172 | Yes | No | [83,85] |
|  | NUTS-3 | ITH34 | Treviso | 45.795 | 12.209 | Yes | No | [83,85,146] |
|  | NUTS-3 | ITH35 | Venezia | 45.696 | 12.831 | Yes | No | [83,85,146] |
|  | NUTS-3 | ITH36 | Padova | 45.350 | 11.798 | Yes | No | [83,146–148] |
|  | NUTS-3 | ITH37 | Rovigo | 45.044 | 11.818 | Yes | No | [83,85] |
|  | NUTS-3 | ITH41 | Pordenone | 46.093 | 12.693 | Yes | No | [83] |
|  | NUTS-3 | ITH42 | Udine | 46.239 | 13.157 | Yes | No | [83,85] |
|  | NUTS-3 | ITH43 | Gorizia | 45.862 | 13.497 | Yes | No | [83] |
|  | NUTS-3 | ITH44 | Trieste | 45.651 | 13.819 | Yes | No | [83,85] |
|  | NUTS-3 | ITH51 | Piacenza | 44.861 | 9.610 | Yes | No | [83] |
|  | NUTS-3 | ITH52 | Parma | 44.676 | 10.026 | Yes | Yes | [83,149,150] |
|  | NUTS-3 | ITH53 | Reggio nell’Emilia | 44.597 | 10.526 | Yes | Yes | [83,150] |
|  | NUTS-3 | ITH54 | Modena | 44.534 | 10.900 | Yes | Yes | [83,85,150–154] |
|  | NUTS-3 | ITH55 | Bologna | 44.424 | 11.359 | Yes | Yes | [83,85,150,155–167] |
|  | NUTS-3 | ITH56 | Ferrara | 44.781 | 11.855 | Yes | Yes | [83,85,165,168] |
|  | NUTS-3 | ITH57 | Ravenna | 44.370 | 12.010 | Yes | Yes | [83,150,165] |
|  | NUTS-3 | ITH58 | Forlì-Cesena | 44.043 | 12.038 | Yes | Yes | [83,85,150,164,169] |
|  | NUTS-3 | ITH59 | Rimini | 43.940 | 12.436 | Yes | Yes | [83,150,158] |
|  | NUTS-3 | ITI11 | Massa-Carrara | 44.251 | 10.012 | Yes | No | [83] |
|  | NUTS-3 | ITI12 | Lucca | 44.007 | 10.431 | Yes | No | [83,170] |
|  | NUTS-3 | ITI13 | Pistoia | 43.972 | 10.850 | Yes | No | [83] |
|  | NUTS-3 | ITI14 | Firenze | 43.817 | 11.291 | Yes | No | [83] |
|  | NUTS-3 | ITI15 | Prato | 43.955 | 11.091 | Yes | No | [83] |
|  | NUTS-3 | ITI16 | Livorno | 43.147 | 10.462 | Yes | No | [83,171] |
|  | NUTS-3 | ITI17 | Pisa | 43.509 | 10.667 | Yes | No | [83,172–178] |
|  | NUTS-3 | ITI18 | Arezzo | 43.540 | 11.856 | Yes | No | [83,179] |
|  | NUTS-3 | ITI19 | Siena | 43.190 | 11.461 | Yes | Yes | [83,85,166,180,181] |
|  | NUTS-3 | ITI1A | Grosseto | 42.780 | 11.234 | Yes | No | [83,85,170,182–184] |
|  | NUTS-3 | ITI21 | Perugia | 43.067 | 12.531 | Yes | No | [83,179,185–187] |
|  | NUTS-3 | ITI22 | Terni | 42.671 | 12.359 | Yes | No | [83] |
|  | NUTS-3 | ITI31 | Pesaro e Urbino | 43.697 | 12.673 | Yes | No | [83] |
|  | NUTS-3 | ITI32 | Ancona | 43.511 | 13.168 | Yes | Yes | [83,100,188,189] |
|  | NUTS-3 | ITI33 | Macerata | 43.193 | 13.253 | Yes | No | [83,179] |
|  | NUTS-3 | ITI34 | Ascoli Piceno | 42.880 | 13.543 | Yes | No | [83] |
|  | NUTS-3 | ITI35 | Fermo | 43.087 | 13.586 | Yes | No | [83,85] |
|  | NUTS-3 | ITI41 | Viterbo | 42.432 | 11.996 | Yes | No | [83] |
|  | NUTS-3 | ITI42 | Rieti | 42.381 | 12.956 | Yes | Yes | [83,190] |
|  | NUTS-3 | ITI43 | Roma | 41.927 | 12.558 | Yes | Yes | [83,85,98,191–193] |
|  | NUTS-3 | ITI44 | Latina | 41.428 | 13.135 | Yes | No | [83] |
|  | NUTS-3 | ITI45 | Frosinone | 41.612 | 13.525 | Yes | No | [83] |
| Kosovo | GAUL-1 | XKG125385 | Pecki | 42.597 | 20.410 | Yes | No | [194] |
|  | GAUL-1 | XKG125389 | Prizremski | 42.246 | 20.720 | Yes | No | [194] |
| Malta | NUTS-3 | MT001 | Malta | 35.888 | 14.428 | Yes | Yes | [195–197] |
|  | NUTS-3 | MT002 | Gozo and Comino/Għawdex u Kemmuna | 36.048 | 14.241 | No | Yes | [196] |
| Montenegro | GAUL-1 | MEG125351 | Bar | 42.160 | 19.150 | No | Yes | [198] |
|  | GAUL-1 | MEG125352 | Berane | 42.858 | 19.895 | No | Yes | [198] |
|  | GAUL-1 | MEG125355 | Cetinje | 42.480 | 18.870 | No | Yes | [198] |
|  | GAUL-1 | MEG125356 | Danilovgrad | 42.595 | 19.099 | No | Yes | [198] |
|  | GAUL-1 | MEG125357 | Herceg Novi | 42.505 | 18.532 | Yes | Yes | [198,199] |
|  | GAUL-1 | MEG125359 | Kotor | 42.535 | 18.649 | No | Yes | [198] |
|  | GAUL-1 | MEG125361 | Niksic | 42.835 | 18.804 | No | Yes | [198] |
|  | GAUL-1 | MEG125365 | Podgorica | 42.472 | 19.334 | No | Yes | [198] |
|  | GAUL-1 | MEG125366 | Rozaj | 42.860 | 20.187 | No | Yes | [198] |
|  | GAUL-1 | MEG125369 | Ulcinj | 41.976 | 19.283 | No | Yes | [198] |
| North Macedonia | NUTS-3 | MK008 | Skopski | 41.959 | 21.455 | Yes | No | [200] |
| Portugal | NUTS-3 | PT111 | Alto Minho | 41.886 | -8.499 | Yes | No | [201–203] |
|  | NUTS-3 | PT112 | Cávado | 41.622 | -8.457 | Yes | No | [201,202] |
|  | NUTS-3 | PT11A | Área Metropolitana do Porto | 41.077 | -8.475 | Yes | Yes | [201,202,204–206] |
|  | NUTS-3 | PT11D | Douro | 41.155 | -7.410 | Yes | Yes | [201,202,205,207–210] |
|  | NUTS-3 | PT11E | Terras de Trás-os-Montes | 41.577 | -6.823 | Yes | No | [201,202] |
|  | NUTS-3 | PT150 | Algarve | 37.242 | -8.133 | Yes | Yes | [201,202,205,211–218] |
|  | NUTS-3 | PT16B | Oeste | 39.280 | -9.128 | Yes | No | [219] |
|  | NUTS-3 | PT16D | Região de Aveiro | 40.620 | -8.514 | Yes | Yes | [201,202,220] |
|  | NUTS-3 | PT16E | Região de Coimbra | 40.215 | -8.319 | Yes | Yes | [201,202,205,220] |
|  | NUTS-3 | PT16F | Região de Leiria | 39.818 | -8.664 | Yes | Yes | [201,205,221] |
|  | NUTS-3 | PT16G | Viseu Dão Lafões | 40.704 | -7.924 | Yes | Yes | [201,202,220] |
|  | NUTS-3 | PT16H | Beira Baixa | 39.898 | -7.424 | Yes | Yes | [201,202,205,220,222] |
|  | NUTS-3 | PT16I | Médio Tejo | 39.603 | -8.271 | Yes | No | [222–225] |
|  | NUTS-3 | PT16J | Beiras e Serra da Estrela | 40.520 | -7.277 | Yes | Yes | [201,202,220,226] |
|  | NUTS-3 | PT170 | Área Metropolitana de Lisboa | 38.731 | -9.045 | Yes | Yes | [201,202,205,211,214,227–250] |
|  | NUTS-3 | PT184 | Baixo Alentejo | 37.872 | -7.804 | Yes | No | [201,202,251] |
|  | NUTS-3 | PT185 | Lezíria do Tejo | 39.120 | -8.610 | Yes | Yes | [201,202,205,220] |
|  | NUTS-3 | PT186 | Alto Alentejo | 39.191 | -7.615 | Yes | Yes | [201,205,252] |
|  | NUTS-3 | PT187 | Alentejo Central | 38.605 | -7.841 | Yes | No | [201,202,253,254] |
| Romania | NUTS-3 | RO224 | Galaţi | 45.799 | 27.767 | Yes | No | [255] |
|  | NUTS-3 | RO311 | Argeş | 45.036 | 24.889 | Yes | No | [256] |
|  | NUTS-3 | RO411 | Dolj | 44.138 | 23.618 | Yes | No | [257] |
|  | NUTS-3 | RO415 | Vâlcea | 45.099 | 24.113 | Yes | No | [258] |
| San Marino | Country | SM | San Marino | 43.937 | 12.463 | Yes | No | [259] |
| Serbia | GAUL-1 | RSG125372 | Branicevski | 44.464 | 21.530 | Yes | No | [260] |
|  | GAUL-1 | RSG125373 | Grad Beograd | 44.657 | 20.425 | Yes | No | [260] |
|  | GAUL-1 | RSG125375 | Juzno-backi | 45.400 | 19.748 | Yes | No | [261] |
|  | GAUL-1 | RSG125383 | Nisavski | 43.405 | 21.876 | No | Yes | [262] |
|  | GAUL-1 | RSG125387 | Podunavski | 44.467 | 20.971 | Yes | No | [260] |
|  | GAUL-1 | RSG125388 | Pomoravski | 43.985 | 21.371 | Yes | No | [260] |
| Slovenia | NUTS-3 | SI037 | Jugovzhodna Slovenija | 45.714 | 15.024 | Yes | No | [263] |
| Spain | NUTS-3 | ES111 | A Coruña | 43.130 | -8.452 | Yes | Yes | [264,265], CMBD^3^ |
|  | NUTS-3 | ES112 | Lugo | 43.015 | -7.447 | Yes | Yes | [264–266], CMBD^3^ |
|  | NUTS-3 | ES113 | Ourense | 42.194 | -7.590 | Yes | Yes | [265,267], CMBD^3^ |
|  | NUTS-3 | ES114 | Pontevedra | 42.442 | -8.455 | Yes | Yes | [265], CMBD^3^ |
|  | NUTS-3 | ES120 | Asturias | 43.297 | -5.986 | Yes | Yes | [264,265,267–270], CMBD^3^ |
|  | NUTS-3 | ES130 | Cantabria | 43.201 | -4.029 | Yes | Yes | [264,265,267,268], CMBD^3^ |
|  | NUTS-3 | ES211 | Araba/Álava | 42.839 | -2.714 | Yes | Yes | [265], CMBD^3^ |
|  | NUTS-3 | ES212 | Gipuzkoa | 43.145 | -2.203 | Yes | Yes | [264], CMBD^3^ |
|  | NUTS-3 | ES213 | Bizkaia | 43.243 | -2.854 | Yes | Yes | [264], CMBD^3^ |
|  | NUTS-3 | ES220 | Navarra | 42.681 | -1.646 | Yes | Yes | [264,265,268], CMBD^3^ |
|  | NUTS-3 | ES230 | La Rioja | 42.279 | -2.504 | Yes | Yes | [265,268], CMBD^3^ |
|  | NUTS-3 | ES241 | Huesca | 42.206 | -0.076 | Yes | Yes | [264,271], CMBD^3^ |
|  | NUTS-3 | ES242 | Teruel | 40.659 | -0.826 | No | Yes | CMBD^3^ |
|  | NUTS-3 | ES243 | Zaragoza | 41.620 | -1.062 | Yes | Yes | [265,272,273], CMBD^3^ |
|  | NUTS-3 | ES300 | Madrid | 40.497 | -3.711 | Yes | Yes | [5,265,268,274–320], CMBD^3^ |
|  | NUTS-3 | ES411 | Ávila | 40.572 | -4.940 | No | Yes | CMBD^3^ |
|  | NUTS-3 | ES412 | Burgos | 42.371 | -3.584 | No | Yes | CMBD^3^ |
|  | NUTS-3 | ES413 | León | 42.626 | -5.839 | No | Yes | CMBD^3^ |
|  | NUTS-3 | ES414 | Palencia | 42.365 | -4.536 | No | Yes | CMBD^3^ |
|  | NUTS-3 | ES415 | Salamanca | 40.809 | -6.062 | Yes | Yes | [265], CMBD^3^ |
|  | NUTS-3 | ES416 | Segovia | 41.166 | -4.057 | No | Yes | CMBD^3^ |
|  | NUTS-3 | ES417 | Soria | 41.627 | -2.595 | Yes | Yes | [321], CMBD^3^ |
|  | NUTS-3 | ES418 | Valladolid | 41.630 | -4.848 | Yes | Yes | [322,323], CMBD^3^ |
|  | NUTS-3 | ES419 | Zamora | 41.735 | -5.984 | Yes | Yes | [265], CMBD^3^ |
|  | NUTS-3 | ES421 | Albacete | 38.826 | -1.981 | Yes | Yes | [265], CMBD^3^ |
|  | NUTS-3 | ES422 | Ciudad Real | 38.928 | -3.823 | Yes | Yes | [265,324], CMBD^3^ |
|  | NUTS-3 | ES423 | Cuenca | 39.898 | -2.195 | No | Yes | CMBD^3^ |
|  | NUTS-3 | ES424 | Guadalajara | 40.815 | -2.626 | Yes | Yes | [265,302,307], CMBD^3^ |
|  | NUTS-3 | ES425 | Toledo | 39.795 | -4.152 | Yes | Yes | [265,307], CMBD^3^ |
|  | NUTS-3 | ES431 | Badajoz | 38.709 | -6.137 | Yes | Yes | [265,325], CMBD^3^ |
|  | NUTS-3 | ES432 | Cáceres | 39.716 | -6.164 | Yes | Yes | [265], CMBD^3^ |
|  | NUTS-3 | ES511 | Barcelona | 41.732 | 1.977 | Yes | Yes | [265,326–344], CMBD^3^ |
|  | NUTS-3 | ES512 | Girona | 42.132 | 2.668 | Yes | Yes | [264,265,345,346], CMBD^3^ |
|  | NUTS-3 | ES513 | Lleida | 42.048 | 1.046 | Yes | Yes | [264,265,347–349], CMBD^3^ |
|  | NUTS-3 | ES514 | Tarragona | 41.089 | 0.817 | Yes | Yes | [265,347,350], CMBD^3^ |
|  | NUTS-3 | ES521 | Alicante/Alacant | 38.486 | -0.570 | Yes | Yes | [265,351–355], CMBD^3^ |
|  | NUTS-3 | ES522 | Castellón/Castelló | 40.238 | -0.150 | Yes | Yes | [265,356], CMBD^3^ |
|  | NUTS-3 | ES523 | Valencia/València | 39.369 | -0.794 | Yes | Yes | [265,354,357–363], CMBD^3^ |
|  | NUTS-3 | ES531 | Eivissa y Formentera | 38.918 | 1.430 | Yes | Yes | [5,265,364], CMBD^3^ |
|  | NUTS-3 | ES532 | Mallorca | 39.615 | 2.962 | Yes | Yes | [5,265,365–371] |
|  | NUTS-3 | ES533 | Menorca | 39.963 | 4.072 | Yes | No | [265,365] |
|  | NUTS-3 | ES611 | Almería | 37.197 | -2.344 | Yes | Yes | [265], CMBD^3^ |
|  | NUTS-3 | ES612 | Cádiz | 36.551 | -5.759 | Yes | Yes | [265,372], CMBD^3^ |
|  | NUTS-3 | ES613 | Córdoba | 37.994 | -4.810 | Yes | Yes | [265], CMBD^3^ |
|  | NUTS-3 | ES614 | Granada | 37.315 | -3.260 | Yes | Yes | [265,373–385], CMBD^3^ |
|  | NUTS-3 | ES615 | Huelva | 37.568 | -6.828 | Yes | Yes | [265], CMBD^3^ |
|  | NUTS-3 | ES616 | Jaén | 38.020 | -3.451 | Yes | Yes | [265], CMBD^3^ |
|  | NUTS-3 | ES617 | Málaga | 36.814 | -4.728 | Yes | Yes | [265], CMBD^3^ |
|  | NUTS-3 | ES618 | Sevilla | 37.433 | -5.683 | Yes | Yes | [265], CMBD^3^ |
|  | NUTS-3 | ES620 | Murcia | 38.001 | -1.479 | Yes | Yes | [265,268,386–397], CMBD^3^ |
|  | NUTS-3 | ES630 | Ceuta | 35.897 | -5.355 | No | Yes | CMBD^3^ |
|  | NUTS-3 | ES640 | Melilla | 35.294 | -2.950 | No | Yes | CMBD^3^ |
| Ukraine | GAUL-1 | UAG13158 | Krym | 45.305 | 34.350 | No | Yes | [398] |

^1^Ministry of Health of Bulgaria. Data provided by the National Centre for Infectious and Parasitic Diseases. Data provided by Dr. Ognyan Mikov.

^2^Ministry of Health of Greece. Data provided by the Hellenic National Public Health Organization in the context of a technical report commissioned and coordinated by ECDC.

^3^National Information System for Hospital Data (Conjunto Mínimo Básico de Datos). Data from 2005 to 2020 requested to the Unit of Health Care Information and Statistics. Institute of Health Information. Spanish Ministry of Health. by e-mail (icmbd@sanidad.gob.es) in February 2022.

**List of references used with known NUTS-3, GAUL-1 or GAUL-2 location data:**

1. Petrela R, Kuneshka L, Foto E, Zavalani F, Gradoni L. Pediatric Visceral Leishmaniasis in Albania: A Retrospective Analysis of 1,210 Consecutive Hospitalized Patients (1995–2009). PLoS Negl Trop Dis. 2010;4: e814.

2. Schüle C, Rehbein S, Shukullari E, Rapti D, Reese S, Silaghi C. Police dogs from Albania as indicators of exposure risk to *Toxoplasma gondii*, *Neospora caninum* and vector-borne pathogens of zoonotic and veterinary concern. Vet Parasitol Reg Stud Rep. 2015;1–2: 35–46.

3. Ayhan N, Velo E, de Lamballerie X, Kota M, Kadriaj P, Ozbel Y, et al. Detection of *Leishmania infantum* and a Novel Phlebovirus (Balkan Virus) from Sand Flies in Albania. Vector Borne Zoonotic Dis. 2016;16: 802–806.

4. Velo E, Bongiorno G, Kadriaj P, Myrseli T, Crilly J, Lika A, et al. The current status of phlebotomine sand flies in Albania and incrimination of *Phlebotomus neglectus* (Diptera, Psychodidae) as the main vector of *Leishmania infantum*. PLoS One. 2017;12: e0179118.

5. Gouzelou E, Haralambous C, Antoniou M, Christodoulou V, Martinković F, Živičnjak T, et al. Genetic diversity and structure in *Leishmania infantum* populations from southeastern Europe revealed by microsatellite analysis. Parasit Vectors. 2013;6: 342.

6. Hamel D, Shukullari E, Rapti D, Silaghi C, Pfister K, Rehbein S. Parasites and vector-borne pathogens in client-owned dogs in Albania. Blood pathogens and seroprevalences of parasitic and other infectious agents. Parasitol Res. 2016;115: 489–499.

7. Silaghi C, Knaus M, Rapti D, Kusi I, Shukullari E, Hamel D, et al. Survey of *Toxoplasma gondii* and *Neospora caninum*, haemotropic mycoplasmas and other arthropod-borne pathogens in cats from Albania. Parasit Vectors. 2014;7: 62.

8. Bizhga B, Laci D, Dhamo G, Keci R, Belegu K, Bakiasi I, et al. Survey for Canine Leishmaniosis. J Anim Vet Adv. 2013;12: 442–446.

9. Obwaller AG, Karakus M, Poeppl W, Töz S, Özbel Y, Aspöck H, et al. Could *Phlebotomus mascittii* play a role as a natural vector for *Leishmania infantum*? New data. Parasit Vectors. 2016;9: 458.

10. Colella V, Hodžić A, Iatta R, Baneth G, Alić A, Otranto D. Zoonotic Leishmaniasis, Bosnia and Herzegovina. Emerg Infect Dis. 2019;25: 385–386.

11. Alić A, Prašović S, Čamo D, Ćoralić A, Preldžić D, Duscher GG, et al. Fatal visceral leishmaniosis in a dog caused by *Leishmania infantum* in Bosnia and Herzegovina: A case report. Vet Parasitol Reg Stud Rep. 2019;15: 100260.

12. Harizanov RN, Kaftandjiev IT, Jordanova DP, Marinova IB, Tsvetkova ND. Clinical features, diagnostic tools, and treatment regimens for visceral leishmaniasis in Bulgaria. Pathog Glob Health. 2013;107: 260–266.

13. Mulić R, Ustović AĆ, Ropac D, Tripković I, Stojanović D, Klišmanić Z. Occurence of Visceral and Cutaneous Leishmaniasis in Croatia. Mil Med. 2009;174: 206–211.

14. Živičnjak T, Martinković F, Khoury C, Bongiorno G, Bosnić S, Lukačević D, et al. Serological and entomological studies of canine leishmaniosis in Croatia. Vet Arh. 2011;81: 99–110.

15. Beljan R, Šundov D, Lukšić B, Šoljić V, Burazer MP. Diagnosis of visceral leishmaniasis by fine needle aspiration cytology of an isolated cervical lymph node: Case report. Coll Antropol. 2010;34: 237–239.

16. Šiško-Kraljević K, Jerončić A, Mohar B, Punda-Polić V. Asymptomatic *Leishmania infantum* infections in humans living in endemic and non-endemic areas of Croatia, 2007 to 2009. Euro Surveill. 2013;18: 20533.

17. Ivović V, Kalan K, Zupan S, Bužan E. Illegal Waste Sites As A Potential Micro Foci Of Mediterranean Leishmaniasis: First Records Of Phlebotomine Sand Flies (Diptera: Psychodidae) From Slovenia. Acta Vet. 2015;65: 348–357.

18. Mrljak V, Kuleš J, Mihaljević Ž, Torti M, Gotić J, Crnogaj M, et al. Prevalence and Geographic Distribution of Vector-Borne Pathogens in Apparently Healthy Dogs in Croatia. Vector Borne Zoonotic Dis. 2017;17: 398–408.

19. Sučić M, Ljubić N, Čulig Z. Cytomorphology of late‐onset Leishmania endophthalmitis: A case report. Cytopathology. 2020;31: 158–160.

20. Attipa C, Papasouliotis K, Solano-Gallego L, Baneth G, Nachum-Biala Y, Sarvani E, et al. Prevalence study and risk factor analysis of selected bacterial, protozoal and viral, including vector-borne, pathogens in cats from Cyprus. Parasit Vectors. 2017;10: 130.

21. Beyhan YE, Celebi B, Ergene O, Mungan M. [Seroprevalance of leishmaniasis in dogs from Hatay and Burdur provinces of Turkey and Northern Cyprus]. Turkiye Parazitol Derg. 2016;40: 9–12.

22. Mazeris A, Soteriadou K, Dedet JP, Haralambous C, Tsatsaris A, Moschandreas J, et al. Leishmaniases and the Cyprus Paradox. Am J Trop Med Hyg. 2010;82: 441–448.

23. Psaroulaki A, Antoniou M, Toumazos P, Mazeris A, Ioannou I, Chochlakis D, et al. Rats as indicators of the presence and dispersal of six zoonotic microbial agents in Cyprus, an island ecosystem: a seroepidemiological study. Trans R Soc Trop Med Hyg. 2010;104: 733–739.

24. Raquin E. Etude retrospective de cas de leishmaniose canine a l’enva de 2000 a 2009. PhD Thesis, Ecole Nationale Veterinaire d’Alfort. 2010.

25. Rolland M, Dinulescu M, Saillard C, Battistella M, Le Gall F, Lhomme F, et al. Nodules ulcérés du visage révélant un lymphome T cutané épidermotrope CD8+ cytotoxique agressif. Ann Dermatol Venereol. 2020;147: 764–768.

26. Chamaillé L, Tran A, Meunier A, Bourdoiseau G, Ready P, Dedet J-P. Environmental risk mapping of canine leishmaniasis in France. Parasit Vectors. 2010;3: 31.

27. Duvignaud A, Receveur M-C, Ezzedine K, Pistone T, Malvy D. Visceral leishmaniasis due to *Leishmania infantum* in a kidney transplant recipient living in France. Travel Med Infect Dis. 2015;13: 115–116.

28. Lachaud L, Dedet JP, Marty P, Faraut F, Buffet P, Gangneux JP, et al. Surveillance of leishmaniases in France, 1999 to 2012. Euro Surveill. 2013;18: 20534.

29. Libert C, Ravel C, Pratlong F, Lami P, Dereure J, Keck N. *Leishmania infantum* infection in two captive Barbary lions (*Panthera leo leo*). J Zoo Wildl Med. 2012;43: 685–688.

30. Collignon C, Zahra A, Guenego L, Gautier R, Madelenat A. Polyarthrite associée à une leishmaniose chez un jeune chien. Prat Med Chir Anim. 2009;44: 27–34.

31. Hurlot Q, Fillaux J, Laurent C, Berry A, Hofman P, Marchou B, et al. A case report of isolated lymphadenopathy revealing localized leishmanial lymphadenopathy in an asthenic 25-year-old man. Medicine. 2016;95: e3932.

32. Bouzouraa T, Cadore JL, Chene J, Goy-Thollot I, Ponce F, Chalvet-Monfray K, et al. Implication, clinical and biological impact of vector-borne haemopathogens in anaemic dogs in France: a prospective study. J Small Anim Pract. 2017;58: 510–518.

33. Lenvers P, Marty P, Peyron F. Ulcération chronique du visage: penser à une leishmaniose cutanée métropolitaine due à *Leishmania infantum*. Ann Dermatol Venereol. 2013;140: 704–707.

34. Martis N, Pomares C, Jeandel PY, Lazure T, Marty P, Rosenthal E. Hepatopulmonary syndrome associated with visceral leishmaniasis. Immunol Res. 2015;61: 169–171.

35. Sevestre J, Reverso-Meinietti J, Butet V, Inedjian JM, Marty P. Progressive dysphonia in a man living in an endemic area for *Leishmania infantum*. Med Mal Infect. 2019;49: 228–230.

36. Vassallo M, Moranne O, Ambrosetti D, Jeandel P-Y, Pomares C, Cassuto E, et al. Visceral leishmaniasis due to *Leishmania infantum* with renal involvement in HIV-infected patients. BMC Infect Dis. 2014;14: 561.

37. Aoun O, Mary C, Roqueplo C, Marié J-L, Terrier O, Levieuge A, et al. Canine leishmaniasis in south-east of France: Screening of *Leishmania infantum* antibodies (western blotting, ELISA) and parasitaemia levels by PCR quantification. Vet Parasitol. 2009;166: 27–31.

38. Bichaud L, Souris M, Mary C, Ninove L, Thirion L, Piarroux RP, et al. Epidemiologic Relationship between Toscana Virus Infection and *Leishmania infantum* Due to Common Exposure to *Phlebotomus perniciosus* Sandfly Vector. PLoS Negl Trop Dis. 2011;5: e1328.

39. Faucher B, Piarroux R, Mary C, Bichaud L, Charrel R, Izri A, et al. Presence of sandflies infected with *Leishmania infantum* and Massilia virus in the Marseille urban area. Clin Microbiol Infect. 2014;20: O340.

40. Davoust B, Mary C, Marié J-L. Detection of Leishmania in Red Foxes (*Vulpes vulpes*) from Southeastern France Using Real-time Quantitative PCR. J Wildl Dis. 2014;50: 130–132.

41. Verneuil M. Leishmaniose oculaire féline: à propos d’un cas. J Fr Ophtalmol. 2013;36: e67–e72.

42. Naucke TJ, Menn B, Massberg D, Lorentz S. Sandflies and leishmaniasis in Germany. Parasitol Res. 2008;103: S65–S68.

43. Athanasiou LV, Kontos VI, Saridomichelakis MN, Rallis TS, Diakou A. A cross-sectional sero-epidemiological study of canine leishmaniasis in Greek mainland. Acta Trop. 2012;122: 291–295.

44. Athanasiou LV, Boutsini SG, Bisia MG. Sandflies and Sandfly Borne Zoonotic Infections in Greece. In: Nriagu J, editor. Encyclopedia of Environmental Health. Elsevier; 2019. pp. 581–588.

45. Boutsini S, Athanasiou LV, Spanakos G, Ntousi D, Dotsika E, Bisia M, et al. Phlebotomine sandflies and factors associated with their abundance in the leishmaniasis endemic area of Attiki, Greece. Parasitol Res. 2018;117: 107–113.

46. Gkolfinopoulou K, Bitsolas N, Patrinos S, Veneti L, Marka A, Dougas G, et al. Epidemiology of human leishmaniasis in Greece, 1981-2011. Euro Surveill. 2013;18: 20532.

47. Iliopoulou P, Tsatsaris A, Katsios I, Panagiotopoulou A, Romaliades S, Papadopoulos B, et al. Risk Mapping of Visceral Leishmaniasis: A Spatial Regression Model for Attica Region, Greece. Trop Med Infect Dis. 2018;3: 83.

48. Kouam MK, Diakou A, Kanzoura V, Papadopoulos E, Gajadhar AA, Theodoropoulos G. A seroepidemiological study of exposure to *Toxoplasma*, *Leishmania*, *Echinococcus* and *Trichinella* in equids in Greece and analysis of risk factors. Vet Parasitol. 2010;170: 170–175.

49. Krepis P, Krepi A, Argyri I, Aggelis A, Soldatou A, Papaevangelou V, et al. Childhood Visceral Leishmaniasis: Distinctive Features and Diagnosis of a Re-emerging Disease. An 11-year Experience from a Tertiary Referral Center in Athens, Greece. Pediatr Infect Dis J. 2018;37: 419–423.

50. Ntais P, Sifaki-Pistola D, Christodoulou V, Messaritakis I, Pratlong F, Poupalos G, et al. Leishmaniases in Greece. Am J Trop Med Hyg. 2013;89: 906–915.

51. Alexandropoulou O, Tsolia M, Kossiva L, Giannaki M, Karavanaki K. Visceral Leishmaniasis. Pediatr Emerg Care. 2012;28: 533–537.

52. Diakou A, Di Cesare A, Accettura PM, Barros L, Iorio R, Paoletti B, et al. Intestinal parasites and vector-borne pathogens in stray and free-roaming cats living in continental and insular Greece. PLoS Negl Trop Dis. 2017;11: e0005335.

53. Georgiadou S, Kontoyiannis D, Sipsas N. Subclinical tumor lysis-like syndrome during treatment of visceral leishmaniasis with low-dose intermittent liposomal amphotericin B. Open Med. 2012;7: 305–309.

54. Georgiadou SP, Stefos A, Spanakos G, Skrimpas S, Makaritsis K, Sipsas NV, et al. Current clinical, laboratory, and treatment outcome characteristics of visceral leishmaniasis: results from a seven-year retrospective study in Greece. Int J Infect Dis. 2015;34: 46–50.

55. Kaziani K, Vadala C, Stasinopoulou P, Loverdos D, Samarkos M, Skoutelis A. Visceral Leishmaniasis Mimicking Lymphoproliferative Disease. South Med J. 2010;103: 1276–1277.

56. Komitopoulou A, Tzenou T, Baltadakis J, Apostolidis J, Karakasis D, Harhalakis N. Is leishmaniasis an “unusual suspect” of infection in allogeneic transplantation? Transpl Infect Dis. 2014;16: 1012–1018.

57. Kritikos K, Haritatos E, Tsigkos S, Gounari P, Skrapari I, Gounaris T, et al. An Atypical Presentation of Visceral Leishmaniasis Infection in a Patient with Rheumatoid Arthritis Treated With Infliximab. J Clin Rheumatol. 2010;16: 38–39.

58. Papadogiannakis E, Spanakos G, Kontos V, Menounos PG, Tegos N, Vakalis N. Molecular Detection of *Leishmania infantum* in Wild Rodents (*Rattus norvegicus*) in Greece. Zoonoses Public Health. 2010;57: e23–e25.

59. Charalampaki N, Tsiveriotis K, Zambou K, Giannopoulou P, Kyratsa A, Yfantis E, et al. Visceral leishmaniasis: Report of 7 cases and review of the literature. Acta Microbiol Hell. 2012;57: 23–30.

60. Tsakmakidis Ι, Pavlou C, Tamvakis Α, Papadopoulos T, Christodoulou V, Angelopoulou K, et al. *Leishmania* infection in lagomorphs and minks in Greece. Vet Parasitol Reg Stud Rep. 2019;16: 100279.

61. Kostopoulou D, Gizzarelli M, Ligda P, Foglia Manzillo V, Saratsi K, Montagnaro S, et al. Mapping the canine vector-borne disease risk in a Mediterranean area. Parasit Vectors. 2020;13: 282.

62. Christodoulou V, Antoniou M, Ntais P, Messaritakis I, Ivovic V, Dedet J-P, et al. Re-Emergence of Visceral and Cutaneous Leishmaniasis in the Greek Island of Crete. Vector Borne Zoonotic Dis. 2012;12: 214–222.

63. Evangelou G, Krasagakis K, Giannikaki E, Kruger-Krasagakis S, Tosca A. Successful treatment of cutaneous leishmaniasis with intralesional aminolevulinic acid photodynamic therapy. Photodermatol Photoimmunol Photomed. 2011;27: 254–256.

64. Papadopoulos E, Angelou A, Diakou A, Halos L, Beugnet F. Five-month serological monitoring to assess the effectiveness of permethrin/fipronil (Frontline Tri-Act®) spot-on in reducing the transmission of *Leishmania infantum* in dogs. Vet Parasitol Reg Stud Rep. 2017;7: 48–53.

65. Papadopoulos E, Angelou A, Madder M, Lebon W, Beugnet F. Experimental assessment of permethrin-fipronil combination in preventing *Leishmania infantum* transmission to dogs under natural exposures. Veterinary parasitology. 2020; 277S: 100026.

66. Diakou A, Papadopoulos E, Lazarides K. Specific anti- *Leishmania* spp. antibodies in stray cats in Greece. J Feline Med Surg. 2009;11: 728–730.

67. Fotakis EA, Giantsis IA, Avgerinou A, Kourtidis S, Agathaggelidou E, Kapoula C, et al. Identification of *Leishmania* species in naturally infected sand flies from refugee camps, Greece. Emerg Infect Dis. 2019;25: 361–364.

68. Oikonomidis IL, Tsouloufi TK, Mylonakis ME, Psalla D, Soubasis N, Rallis T, et al. Circulating and bone marrow myeloid cells containing *Leishmania* amastigotes in a case of advanced canine leishmaniosis. J Vet Diagn Invest. 2019;31: 726–731.

69. Theodorou K, Mylonakis ME, Farmaki R, Patsikas MN, Loukopoulos P, Poutahidis T, et al. Cutaneous transmissible venereal tumor with internal metastases in two dogs. J Hell Vet Medical Soc. 2012;63: 30–36.

70. Chaskopoulou A, Miaoulis M, Kashefi J. Ground ultra low volume (ULV) space spray applications for the control of wild sand fly populations (Psychodidae: Phlebotominae) in Europe. Acta Trop. 2018;182: 54–59.

71. Tsokana CN, Sokos C, Giannakopoulos A, Mamuris Z, Birtsas P, Papaspyropoulos K, et al. First evidence of *Leishmania* infection in European brown hare (*Lepus europaeus*) in Greece: GIS analysis and phylogenetic position within the *Leishmania* spp. Parasitol Res. 2016;115: 313–321.

72. Liberopoulos E, Kei A, Apostolou F, Elisaf M. Autoimmune manifestations in patients with visceral leishmaniasis. J Microbiol Immunol Infect. 2013;46: 302–305.

73. Athanasiou LV, Chatzis MK, Gouletsou PG, Saridomichelakis MN. Sensitivity of preputial and vaginal exfoliative cytological examination for diagnosis of canine leishmaniosis (*Leishmania infantum*). J Hell Vet Medical Soc. 2014;65: 31–34.

74. Giannakopoulos A, Tsokana CN, Pervanidou D, Papadopoulos E, Papaspyropoulos K, Spyrou V, et al. Environmental parameters as risk factors for human and canine *Leishmania* infection in Thessaly, Central Greece. Parasitology. 2016;143: 1179–1186.

75. Giannakopoulos A, Tsokana CN, Papadopoulos E, Spyrou V, Chatzopoulos DC, Valiakos G, et al. Molecular investigation and geographical distribution of *Leishmania* spp infection in stray and owned cats (*Felis catus*) in Thessaly, central Greece. J Hell Vet Medical Soc. 2017;68: 27–34.

76. Kasabalis D, Chatzis MK, Apostolidis K, Petanides T, Athanasiou LV, Xenoulis PG, et al. A randomized, blinded, controlled clinical trial comparing the efficacy of aminosidine (paromomycin)-allopurinol combination with the efficacy of meglumine antimoniate-allopurinol combination for the treatment of canine leishmaniosis due to *Leishmania* *infantum*. Exp Parasitol. 2020;214: 107903.

77. Saridomichelakis MN, Apostolidis KN, Chatzis MK, Petanides T, Kokkinaki K, Athanasiou LV, et al. Are stray dogs confined in animal shelters at increased risk of seropositivity to *Leishmania infantum*? A case control study. Rev Med Vet. 2018;169: 12–23.

78. Makaritsis KP, Gatselis NK, Ioannou M, Petinaki E, Dalekos GN. Polyclonal hypergammaglobulinemia and high smooth-muscle autoantibody titers with specificity against filamentous actin: consider visceral leishmaniasis, not just autoimmune hepatitis. Int J Infect Dis. 2009;13: e157–e160.

79. Kasabalis D, Chatzis MK, Apostolidis K, Xenoulis PG, Buono A, Petanides T, et al. Evaluation of nephrotoxicity and ototoxicity of aminosidine (paromomycin)-allopurinol combination in dogs with leishmaniosis due to *Leishmania infantum*: A randomized, blinded, controlled study. Exp Parasitol. 2019;206.

80. Karayiannis S, Ntais P, Messaritakis I, Tsirigotakis N, Dokianakis E, Antoniou M. Detection of *Leishmania infantum* in red foxes (*Vulpes vulpes*) in Central Greece. Parasitology. 2015;142: 1574–1578.

81. Tánczos B, Balogh N, Király L, Biksi I, Szeredi L, Gyurkovsky M, et al. First Record of Autochthonous Canine Leishmaniasis in Hungary. Vector Borne Zoonotic Dis. 2012;12: 588–594.

82. Ferroglio E, Battisti E, Zanet S, Bolla C, Concialdi E, Trisciuoglio A, et al. Epidemiological evaluation of *Leishmania infantum* zoonotic transmission risk in the recently established endemic area of Northwestern Italy. Zoonoses Public Health. 2018;65: 675–682.

83. Mendoza-Roldan J, Benelli G, Panarese R, Iatta R, Furlanello T, Beugnet F, et al*. Leishmania infantum* and *Dirofilaria immitis* infections in Italy, 2009–2019: changing distribution patterns. Parasit Vectors. 2020;13: 193.

84. Biglino A, Bolla C, Concialdi E, Trisciuoglio A, Romano A, Ferroglio E. Asymptomatic *Leishmania infantum* infection in an area of northwestern Italy (Piedmont region) where such infections are traditionally nonendemic. J Clin Microbiol. 2010;48: 131–136.

85. Iatta R, Furlanello T, Colella V, Tarallo VD, Latrofa MS, Brianti E, et al. A nationwide survey of *Leishmania infantum* infection in cats and associated risk factors in Italy. PLoS Negl Trop Dis. 2019;13: e0007594.

86. Pomares-Estran C, Cenderello G, Ittel A, Karsenti JM, Cardot-Leccia N, Vassalo M, et al. Isolated lymphadenopathy in *Leishmania infantum* infection: three case reports. Ann Trop Med Parasitol. 2009;103: 555–559.

87. Rongioletti F, Cannata GE, Parodi A. Leishmaniasis due to *L. infantum* presenting as macrocheilitis and responding to liposomal amphotericin B. Eur J Dermatol. 2009;19: 281–282.

88. Cenderello G, Pontali E, Ruggeri C, Dusi A, De Maria A. Unusual Presentation of Visceral Leishmaniasis in an HIV-Infected Patient. AIDS Res Hum Retrovir. 2014;30: 846–847.

89. Spada E, Proverbio D, Groppetti D, Perego R, Grieco V, Ferro E. First report of the use of meglumine antimoniate for treatment of canine leishmaniasis in a pregnant dog. J Am Anim Hosp Assoc. 2011;47: 67–71.

90. Della Pepa A. Vector-borne diseases in colony stray cats of Milan city. PhD Thesis, Università degli Studi di Milano. 2013.

91. Perego R, Proverbio D, Bagnagatti De Giorgi G, Spada E. Prevalence of Dermatological Presentations of Canine Leishmaniasis in a Nonendemic Area: A Retrospective Study of 100 Dogs. Vet Med Int. 2014;2014: 1–5.

92. Proverbio D, Spada E, Perego R, Baggiani L, Bagnagatti De Giorgi G, Migliazzo A, et al. Comparison of a rapid immunochromatographic assay with an immunofluorescent antibody test for detection of *Leishmania infantum* antibodies in dogs. Vet Clin Pathol. 2016;45: 623–626.

93. Proverbio D, Spada E, Bagnagatti de Giorgi G, Perego R, Valena E. Relationship between *Leishmania* IFAT Titer and Clinicopathological Manifestations (Clinical Score) in Dogs. Biomed Res Int. 2014;2014: 412808.

94. Spada E, Canzi I, Baggiani L, Perego R, Vitale F, Migliazzo A, et al. Prevalence of *Leishmania infantum* and co-infections in stray cats in northern Italy. Comp Immunol Microbiol Infect Dis. 2016;45: 53–58.

95. Spada E, Perego R, Vitale F, Bruno F, Castelli G, Tarantola G, et al. Feline *Leishmania* spp. Infection in a Non-Endemic Area of Northern Italy. Animals. 2020;10: 817.

96. Boari A, Pierantozzi M, Aste G, Pantaleo S, Di Silverio F, Fanini G, et al. The Association Between N-Methylglucamine Antimoniate and Pancreatitis in Dogs with Leishmaniasis. In: Pugliese A, Gaiti A, Boiti C, editors. Veterinary Science. Berlin, Heidelberg: Springer; 2012. pp. 65–69.

97. Podaliri Vulpiani M, Iannetti L, Di Mattia T, Dalla Villa P. *Leishmania infantum* in a Central Italy dog shelter: Retrospective study of serologic reactivity during a 4-year period in a confined dog population subjected to preventive and therapeutic treatment. Vet Parasitol. 2009;160: 190–197.

98. Traversa D, Di Cesare A, Simonato G, Cassini R, Merola C, Diakou A, et al. Zoonotic intestinal parasites and vector-borne pathogens in Italian shelter and kennel dogs. Comp Immunol Microbiol Infect Dis. 2017;51: 69–75.

99. Gizzarelli M, Foglia Manzillo V, Ciuca L, Morgoglione ME, El Houda Ben Fayala N, Cringoli G, et al. Simultaneous Detection of Parasitic Vector Borne Diseases: A Robust Cross-Sectional Survey in Hunting, Stray and Sheep Dogs in a Mediterranean Area. Front Vet Sci. 2019;6: 288.

100. Tilli M, Botta A, Bartoloni A, Corti G, Zammarchi L. Hospitalization for Chagas disease, dengue, filariasis, leishmaniasis, schistosomiasis, strongyloidiasis, and *Taenia* *solium* taeniasis/cysticercosis, Italy, 2011–2016. Infection. 2020;48: 695–713.

101. Gramiccia M, Di Muccio T, Fiorentino E, Scalone A, Bongiorno G, Cappiello S, et al. Longitudinal study on the detection of canine *Leishmania* infections by conjunctival swab analysis and correlation with entomological parameters. Vet Parasitol. 2010;171: 223–228.

102. Bongiorno G, Paparcone R, Manzillo VF, Oliva G, Cuisinier A-M, Gradoni L. Vaccination with LiESP/QA-21 (CaniLeish®) reduces the intensity of infection in *Phlebotomus perniciosus* fed on *Leishmania infantum* infected dogs—A preliminary xenodiagnosis study. Vet Parasitol. 2013;197: 691–695.

103. Foglia Manzillo V, Paparcone R, Cappiello S, De Santo R, Bianciardi P, Oliva G. Resolution of tongue lesions caused by *Leishmania infantum* in a dog treated with the association miltefosine-allopurinol. Parasit Vectors. 2009;2 Suppl 1: S6.

104. Foglia Manzillo V, Di Muccio T, Cappiello S, Scalone A, Paparcone R, Fiorentino E, et al. Prospective Study on the Incidence and Progression of Clinical Signs in Naïve Dogs Naturally Infected by *Leishmania infantum*. PLoS Negl Trop Dis. 2013;7: e2225.

105. Kostalova T, Lestinova T, Sumova P, Vlkova M, Rohousova I, Berriatua E, et al. Canine Antibodies against Salivary Recombinant Proteins of *Phlebotomus perniciosus*: A Longitudinal Study in an Endemic Focus of Canine Leishmaniasis. PLoS Negl Trop Dis. 2015;9: e0003855.

106. Manna L, Reale S, Vitale F, Gravino AE. Evidence for a relationship between *Leishmania* load and clinical manifestations. Res Vet Sci. 2009;87: 76–78.

107. Manna L, Paciello O, Morte RD, Gravino AE. Detection of *Leishmania* parasites in the testis of a dog affected by orchitis: case report. Parasit Vectors. 2012;5: 216.

108. Mekuzas Y, Gradoni L, Oliva G, Manzillo VF, Baneth G. Ehrlichia canis and *Leishmania infantum* co-infection: a 3-year longitudinal study in naturally exposed dogs. Clin Microbiol Infect. 2009;15: 30–31.

109. Maio P, Leone S, Volpe S, Dell’Aquila G, Giglio S, Magliocca M, et al. Visceral Leishmaniasis in a patient with common variable immunodeficiency and Evans syndrome: Clinical remarks. New Microbiol. 2009;32: 223–227.

110. Piantedosi D, Veneziano V, Di Muccio T, Manzillo VF, Fiorentino E, Scalone A, et al. Epidemiological survey on *Leishmania* infection in red foxes (*Vulpes vulpes*) and hunting dogs sharing the same rural area in Southern Italy. Acta Parasitol. 2016;61: 769–775.

111. Iatta R, Zatelli A, Laricchiuta P, Legrottaglie M, Modry D, Dantas-Torres F, et al. *Leishmania infantum* in Tigers and Sand Flies from a Leishmaniasis- Endemic Area, Southern Italy. Emerg Infect Dis. 2020;26: 1311–1314.

112. Lorusso V, Dantas-Torres F, Caprio F, Manzionna M, Santoro N, Baneth G, et al. Paediatric Visceral Leishmaniasis in Italy: a ‘One Health’ approach is needed. Parasit Vectors. 2013;6: 123.

113. Latrofa MS, Dantas-Torres F, de Caprariis D, Cantacessi C, Capelli G, Lia RP, et al. Vertical transmission of *Anaplasma platys* and *Leishmania infantum* in dogs during the first half of gestation. Parasit Vectors. 2016;9: 269.

114. Latrofa MS, Iatta R, Dantas-Torres F, Annoscia G, Gabrielli S, Pombi M, et al. Detection of *Leishmania infantum* DNA in phlebotomine sand flies from an area where canine leishmaniosis is endemic in southern Italy. Vet Parasitol. 2018;253: 39–42.

115. Monno R, Giannelli G, Rizzo C, De Vito D, Fumarola L. Recombinant K39 immunochromatographic test for diagnosis of human leishmaniasis. Future Microbiol. 2009;4: 159–170.

116. Otranto D, Testini G, Dantas-Torres F, Latrofa MS, Diniz PPV d P, de Caprariis D, et al. Diagnosis of Canine Vector-Borne Diseases in Young Dogs: a Longitudinal Study. J Clin Microbiol. 2010;48: 3316–3324.

117. Otranto D, Dantas-Torres F, de Caprariis D, Di Paola G, Tarallo VD, Latrofa MS, et al. Prevention of Canine Leishmaniosis in a Hyper-Endemic Area Using a Combination of 10% Imidacloprid/4.5% Flumethrin. PLoS One. 2013;8: e56374.

118. Dantas-Torres F, Lorusso V, Testini G, de Paiva-Cavalcanti M, Figueredo LA, Stanneck D, et al. Detection of *Leishmania infantum* in *Rhipicephalus sanguineus* ticks from Brazil and Italy. Parasitol Res. 2010;106: 857–860.

119. Dantas-Torres F, Tarallo VD, Latrofa MS, Falchi A, Lia RP, Otranto D. Ecology of phlebotomine sand flies and *Leishmania infantum* infection in a rural area of southern Italy. Acta Trop. 2014;137: 67–73.

120. Persichetti M-F, Solano-Gallego L, Serrano L, Altet L, Reale S, Masucci M, et al. Detection of vector-borne pathogens in cats and their ectoparasites in southern Italy. Parasit Vectors. 2016;9: 247.

121. Allegra E, Franco T, Trapasso S, Caroleo B, Foca A, Amorosi A, et al. Rare association of laryngeal precancerosis and laryngeal leishmaniasis. Gazz Med Ital Arch Sci Med. 2012;171: 823–827.

122. Piro E, Kropp M, Cantaffa R, Lamberti AG, Carillio G, Molica S. Visceral leishmaniasis infection in a refractory multiple myeloma patient treated with bortezomib. Ann Hematol. 2012;91: 1827–1828.

123. Amodio E, Calamusa G, Sidoti E, Migliazzo A, Vitale F, Costantino C. Seroprevalence of and risk factors for *Leishmania* seropositivity in a sample population of Western Sicily (Italy). Epidemiol Biostat Public Health. 2014;11: e9024.

124. Abbate JM, Arfuso F, Napoli E, Gaglio G, Giannetto S, Latrofa MS, et al. *Leishmania infantum* in wild animals in endemic areas of southern Italy. Comp Immunol Microbiol Infect Dis. 2019;67: 101374.

125. Bongiorno MR, Pistone G, Aricò M. Unusual clinical variants of cutaneous leishmaniasis in Sicily. Int J Dermatol. 2009;48: 286–289.

126. Colomba C, Saporito L, Vitale F, Reale S, Vitale G, Casuccio A, et al. Cryptic *Leishmania infantum* infection in Italian HIV infected patients. BMC Infect Dis. 2009;9: 199.

127. Colomba C, Saporito L, Di Carlo P, Tolomeo M, Cervo A, Firenze A, et al. Direct-acting antivirals and visceral leishmaniasis: a case report. BMC Infect Dis. 2019;19: 328.

128. Pietro SD, Bosco VRF, Crinò C, Francaviglia F, Giudice E. Prevalence, type, and prognosis of ocular lesions in shelter and owned client dogs naturally infected by *Leishmania infantum*. Vet World. 2016;9: 633–637.

129. Priolo V, Martínez-Orellana P, Pennisi MG, Masucci M, Prandi D, Ippolito D, et al. *Leishmania infantum* -specific IFN-γ production in stimulated blood from cats living in areas where canine leishmaniosis is endemic. Parasit Vectors. 2019;12: 133.

130. Abbate JM, Maia C, Pereira A, Arfuso F, Gaglio G, Rizzo M, et al. Identification of trypanosomatids and blood feeding preferences of phlebotomine sand fly species common in Sicily, Southern Italy. PLoS One. 2020;15: e0229536.

131. Brianti E, Gaglio G, Napoli E, Falsone L, Prudente C, Solari Basano F, et al. Efficacy of a slow-release imidacloprid (10%)/flumethrin (4.5%) collar for the prevention of canine leishmaniosis. Parasit Vectors. 2014;7: 327.

132. Brianti E, Falsone L, Napoli E, Gaglio G, Giannetto S, Pennisi MG, et al. Prevention of feline leishmaniosis with an imidacloprid 10%/flumethrin 4..5% polymer matrix collar. Parasit Vectors. 2017;10: 334.

133. Brianti E, Celi N, Napoli E, Abbate JM, Arfuso F, Gaglio G, et al. Treatment and long-term follow-up of a cat with leishmaniosis. Parasit Vectors. 2019;12: 121.

134. Giudice E, Passantino A. Detection of *Leishmania* amastigotes in peripheral blood from four dogs — Short communication. Acta Vet Hung. 2011;59: 205–213.

135. Marino G, Gaglio G, Zanghì A. Clinicopathological study of canine transmissible venereal tumour in leishmaniotic dogs. J Small Anim Pract. 2012;53: 323–327.

136. Otranto D, Napoli E, Latrofa MS, Annoscia G, Tarallo VD, Greco G, et al. Feline and canine leishmaniosis and other vector-borne diseases in the Aeolian Islands: Pathogen and vector circulation in a confined environment. Vet Parasitol. 2017;236: 144–151.

137. Foglia Manzillo V, Gizzarelli M, Vitale F, Montagnaro S, Torina A, Sotera S, et al. Serological and entomological survey of canine leishmaniasis in Lampedusa island, Italy. BMC Vet Res. 2018;14: 286.

138. Baglieri F, Scuderi G. A case of mucosal leishmaniasis of the tongue in a kidney transplant recipient. Int J Dermatol. 2012;51: 597–600.

139. Lisi O, D’Urso V, Vaccalluzzo V, Bongiorno G, Khoury C, Severini F, et al. Persistence of phlebotomine *Leishmania* vectors in urban sites of Catania (Sicily, Italy). Parasit Vectors. 2014;7: 560.

140. Strazzulla A, Cocuzza S, Pinzone MR, Francesco M, Serra A, Cosentino S, et al. Isolated laryngeal leishmaniasis in a 55-year-old man with dysphonia and rheumatoid arthritis: Case report and literature review. Acta Med Mediterr. 2013;29: 807–810.

141. Dedola C, Zobba R, Varcasia A, Visco S, Alberti A, Pipia AP, et al. Serological and molecular detection of *Leishmania infantum* in cats of Northern Sardinia, Italy. Vet Parasitol Regi Stud Rep. 2018;13: 120–123.

142. Madeddu G, Fiori ML, Ena P, Riu F, Lovigu C, Nunnari G, et al. Mucocutaneous leishmaniasis as presentation of HIV infection in Sardinia, insular Italy. Parasitol Int. 2014;63: 35–36.

143. Ennas F, Calderone S, Capri A, Pennisi MG. Case report of Leishmaniosis in a cat from Sardinia (Italy). Veterinaria. 2012;26: 55–59.

144. Losa F, Firinu D, Deidda M, Costanzo G, del Giacco SR. Clinical pitfalls of leishmaniasis and Whipple’s disease hidden behind systemic lupus erythematosus: A case series. Acta Microbiol Immunol Hung. 2019;66: 377–385.

145. Pau M, Atzori L, Aste N. Two cases of primary endonasal leishmaniasis in Sardinia (Italy). Dermatol Online J. 2009;15: 5.

146. Vascellari M, Ravagnan S, Carminato A, Cazzin S, Carli E, Da Rold G, et al. Exposure to vector-borne pathogens in candidate blood donor and free-roaming dogs of northeast Italy. Parasit Vectors. 2016;9: 369.

147. Cassini R, Signorini M, di Regalbono AF, Natale A, Montarsi F, Zanaica M, et al. Preliminary study of the effects of preventive measures on the prevalence of Canine Leishmaniosis in a recently established focus in northern Italy. Vet Ital. 2013;49: 157–161.

148. Solano-Gallego L, Villanueva-Saz S, Carbonell M, Trotta M, Furlanello T, Natale A. Serological diagnosis of canine leishmaniosis: comparison of three commercial ELISA tests (Leiscan®, ID Screen® and Leishmania 96®), a rapid test (Speed Leish K®) and an in-house IFAT. Parasit Vectors. 2014;7: 111.

149. Calderaro A, Montecchini S, Rossi S, Gorrini C, Dell’Anna M, Piccolo G, et al. A 22-Year Survey of Leishmaniasis Cases in a Tertiary-Care Hospital in an Endemic Setting. Int J Environ Res Public Health. 2014;11: 2834–2845.

150. Varani S, Ortalli M, Attard L, Vanino E, Gaibani P, Vocale C, et al. Serological and molecular tools to diagnose visceral leishmaniasis: 2-years’ experience of a single center in Northern Italy. PLoS One. 2017;12: e0183699.

151. Cesinaro AM, Nosseir S, Mataca E, Mengoli MC, Cavatorta C, Gennari W. An outbreak of cutaneous leishmaniasis in Modena province (Northern Italy): report of 35 cases. Pathologica. 2017;109: 363–367.

152. Franceschini E, Puzzolante C, Menozzi M, Rossi L, Bedini A, Orlando G, et al. Clinical and Microbiological Characteristics of Visceral Leishmaniasis Outbreak in a Northern Italian Nonendemic Area: A Retrospective Observational Study. Biomed Res Int. 2016;2016: 1–7.

153. Veraldi S, Nazzaro G. Successful treatment of cutaneous leishmaniasis of the ear with oral fluconazole. Clin Exp Dermatol. 2016;41: 315–316.

154. Veraldi S, Benzecry Mancin V, Faraci AG, Nazzaro G. Unsuccessful treatment of cutaneous leishmaniasis by *Leishmania infantum* with topical paromomycin. Int J Dermatol. 2020;59: e251–e252.

155. Baldelli R, Piva S, Salvatore D, Parigi M, Melloni O, Tamba M, et al. Canine leishmaniasis surveillance in a northern Italy kennel. Vet Parasitol. 2011;179: 57–61.

156. Calzolari M, Carra E, Rugna G, Bonilauri P, Bergamini F, Bellini R, et al. Isolation and Molecular Typing of *Leishmania infantum* from *Phlebotomus perfiliewi* in a Re-Emerging Focus of Leishmaniasis, Northeastern Italy. Microorganisms. 2019;7: 644.

157. Comai G, Mistral De Pascali A, Busutti M, Morini S, Ortalli M, Conte D, et al. Screening strategies for the diagnosis of asymptomatic *Leishmania* infection in dialysis patients as a model for kidney transplant candidates. J Nephrol. 2021;34: 191–195.

158. Salvatore D. Esperienza all’interno di un progetto per la realizzazione di un sistema di sorveglianza della leishmaniosi canina in Emilia-Romagna. PhD Thesis, Università di Bologna. 2010.

159. Gaspari V, Ortalli M, Foschini MP, Baldovini C, Lanzoni A, Cagarelli R, et al. New evidence of cutaneous leishmaniasis in north-eastern Italy. J Eur Acad Dermatol Venereol. 2017;31: 1534–1540.

160. Ortalli M. Epidemiological, Diagnostic and Drug Discovery Studies of an Emerging Infection: Human Leishmaniasis. PhD Thesis, Università di Bologna. 2017.

161. Melchionda F, Varani S, Carfagnini F, Belotti T, Di Muccio T, Tigani R, et al. Spleen nodules: a potential hallmark of Visceral Leishmaniasis in young children. BMC Infects Dis. 2014;14: 620.

162. Ortalli M, De Pascali AM, Longo S, Pascarelli N, Porcellini A, Ruggeri D, et al. Asymptomatic *Leishmania infantum* infection in blood donors living in an endemic area, northeastern Italy. J Infect. 2020;80: 116–120.

163. Rugna G, Carra E, Corpus F, Calzolari M, Salvatore D, Bellini R, et al. Distinct *Leishmania infantum* Strains Circulate in Humans and Dogs in the Emilia–Romagna Region, Northeastern Italy. Vector Borne Zoonotic Dis. 2017;17: 409–415.

164. Salvatore D, Aureli S, Baldelli R, Di Francesco A, Tampieri MP, Galuppi R. Molecular evidence of *Leishmania infantum* in *Ixodes ricinus* ticks from dogs and cats, in Italy. Vet Ital. 2014;50: 307–312.

165. Urbani L, Tirolo A, Salvatore D, Tumbarello M, Segatore S, Battilani M, et al. Serological, molecular and clinicopathological findings associated with *Leishmania* *infantum* infection in cats in Northern Italy. J Feline Med Surg. 2020;22: 935–943.

166. Varani S, Cagarelli R, Melchionda F, Attard L, Salvadori C, Finarelli AC, et al. Ongoing outbreak of visceral leishmaniasis in Bologna Province, Italy, November 2012 to May 2013. Euro Surveill. 2013;18: 20530.

167. Zanoni L, Varani S, Attard L, Morigi JJ, Vanino E, Ortalli M, et al. 18F-FDG PET/CT in visceral leishmaniasis: uptake patterns in the context of a multiannual outbreak in Northern Italy. Ann Nucl Med. 2019;33: 716–723.

168. De Leonardis F, Govoni M, Lo Monaco A, Trotta F. Visceral leishmaniasis and anti-TNF-alpha therapy: case report and review of the literature. Clin Exp Rheumatol. 2009;27: 503–6.

169. Mastroianni A, Gaibani P, Rossini G, Vocale C, Re MC, Ravaglia G, et al. Two cases of relapsed HIV-associated visceral leishmaniasis successfully treated with combination therapy. AIDS ResTher. 2018;15: 27.

170. Tondi L. La Leishmaniosi felina: rassegna di casi clinici e indagine parassitologica in soggetti viventi in zone endemiche. PhD Thesis, Università di Pisa. 2010.

171. Zanet S, Sposimo P, Trisciuoglio A, Giannini F, Strumia F, Ferroglio E. Epidemiology of *Leishmania infantum*, *Toxoplasma gondii*, and *Neospora caninum* in *Rattus rattus* in absence of domestic reservoir and definitive hosts. Vet Parasitol. 2014;199: 247–249.

172. Ariti G, Nardoni S, Papini R, Mugnaini L, Giannetti G, Bizzeti M, et al. Treatment of canine leishmaniasis: Long term molecular and serological observations. Med Weter. 2013;69: 109–111.

173. Bertuccelli Fanucchi M. Leishmaniosi della volpe in Toscana: indagine sierologica e molecolare su soggetti abbattuti nella provincia di Pisa. PhD Thesis, Università di Pisa. 2009.

174. Ebani VV, Poli A, Rocchigiani G, Bertelloni F, Nardoni S, Papini RA, et al. Serological survey on some pathogens in wild brown hares (*Lepus europaeus*) in Central Italy. Asian Pac J Trop Med. 2016;9: 465–469.

175. Rocchigiani G, Ebani VV, Nardoni S, Bertelloni F, Bascherini A, Leoni A, et al. Molecular survey on the occurrence of arthropod-borne pathogens in wild brown hares (*Lepus europaeus*) from Central Italy. Infect Genet Evol. 2018;59: 142–147.

176. Sbrana S, Marchetti V, Mancianti F, Guidi G, Bennett D. Retrospective study of 14 cases of canine arthritis secondary to *Leishmania* infection. J Small Anim Pract. 2014;55: 309–313.

177. Vannucci V, Gavazza A, Medina Valentin AA, Gori V, Lubas G. Hematological, biochemical, serological, and molecular monitoring of blood donor dogs vaccinated with CaniLeish® for the prevention of Leishmaniosis. Comp Clin Pathol. 2018;27: 1173–1179.

178. Verin R, Poli A, Ariti G, Nardoni S, Fanucchi MB, Mancianti F. Detection of *Leishmania infantum* DNA in tissues of free-ranging red foxes (*Vulpes vulpes*) in Central Italy. Eur J Wildl Res. 2010;56: 689–692.

179. Morganti G, Veronesi F, Stefanetti V, Di Muccio T, Fiorentino E, Diaferia M, et al. Emerging feline vector-borne pathogens in Italy. Parasit Vectors. 2019;12: 193.

180. Tordini G, Giaccherini R, Sammarro G, Braito A, Zanelli G. Human leishmaniasis in Tuscany: a changing pattern of visceral disease? Ann Trop Med Parasitol. 2010;104: 171–174.

181. Tordini G, Puttini C, Rossetti B, Sammarro G, Fanetti A, Cianchino S, et al. *Leishmania infantum* e donatori di sangue: Quale screening? Infez Med. 2011;19: 152–156.

182. Ebani VV, Nardoni S, Fognani G, Mugnaini L, Bertelloni F, Rocchigiani G, et al. Molecular detection of vector-borne bacteria and protozoa in healthy hunting dogs from Central Italy. Asian Pac J Trop Biomed. 2015;5: 108–112.

183. Nardoni S, Altomonte I, Salari F, Martini M, Mancianti F. Serological and Molecular Findings of *Leishmania* Infection in Healthy Donkeys (*Equus asinus*) from a Canine Leishmaniosis Endemic Focus in Tuscany, Italy: A Preliminary Report. Pathogens. 2019;8: 99.

184. Solano-Gallego L, Rossi L, Scroccaro AM, Montarsi F, Caldin M, Furlanello T, et al. Detection of Leishmania infantum DNA mainly in *Rhipicephalus sanguineus* male ticks removed from dogs living in endemic areas of canine leishmaniosis. Parasit Vectors. 2012;5: 98.

185. Antognoni MT, Birettoni F, Miglio A, Lalli P, Porciello F, Mangili Pecci V. Monoclonal gammopathy associated with multiple myeloma and visceral leishmaniasis in the dog: A comparison of two cases. Vet Res Comm. 2010;34: 97–101.

186. Maresca C, Scoccia E, Barizzone F, Catalano A, Mancini S, Pagliacci T, et al. A survey on canine leishmaniasis and phlebotomine sand flies in central Italy. Res Vet Sci. 2009;87: 36–38.

187. Tascini G, Lanciotti L, Sebastiani L, Paglino A, Esposito S. Complex Investigation of a Pediatric Haematological Case: Haemophagocytic Syndrome Associated with Visceral Leishmaniasis and Epstein–Barr (EBV) Co-Infection. Int J Environ Res Public Health. 2018;15: 2672.

188. Ceccarelli M, Galluzzi L, Sisti D, Bianchi B, Magnani M. Application of qPCR in conjunctival swab samples for the evaluation of canine leishmaniasis in borderline cases or disease relapse and correlation with clinical parameters. Parasit Vectors. 2014;7: 460.

189. Ceccarelli M, Galluzzi L, Migliazzo A, Magnani M. Detection and Characterization of *Leishmania* (*Leishmania*) and *Leishmania* (*Viannia*) by SYBR Green-Based Real-Time PCR and High Resolution Melt Analysis Targeting Kinetoplast Minicircle DNA. PLoS One. 2014;9: e88845.

190. Barlozzari G. Presenza e diffusione della Leishmaniosi e dei suoi vettori in provincia di Rieti. PhD Thesis, Università di Bologna. 2015.

191. D’Angio M, Ceglie T, Giovannetti G, Neri A, Santilio I, Nunes V, et al. Visceral leishmaniasis presenting with paroxysmal cold haemoglobinuria. Blood Transfus. 2014;12: 141–143.

192. Famularo G, Mancini S. Visceral Leishmaniasis. Mayo Clin Proc. 2016;91: 1322–1323.

193. Scalzone M, Ruggiero A, Mastrangelo S, Trombatore G, Ridola V, Maurizi P, et al. Hemophagocytic lymphohistiocytosis and visceral leishmaniasis in children: case report and systematic review of literature. J Infect Dev Ctries. 2016;10: 103–108.

194. Xhekaj B, Alishani M, Rexhepi A, Jakupi X, Sherifi K. Serological survey of canine leishmaniasis in southwestern region of Kosovo. Vet Ital. 2020;56: 47–50.

195. Micallef C, Azzopardi CM. Atypical cutaneous leishmaniasis in the immunosuppressed. BMJ Case Rep. 2014; 2014:bcr201404914.

196. Pace D, Williams TN, Grochowska A, Betts A, Attard-Montalto S, Boffa MJ, et al. Manifestations of paediatric *Leishmania infantum* infections in Malta. Travel Med Infect Dis. 2011;9: 37–46.

197. Puidokienè E. Canine leishmaniasis. Diagnostics, treatment and risk factors. PhD Thesis, Lithuanian University of Health Sciences. 2015.

198. Medenica S, Jovanović S, Dožić I, Miličić B, Lakićević N, Rakočević B. Epidemiological surveillance of leishmaniasis in Montenegro, 1992–2013. Srp Arha Celok Lek. 2015;143: 707–711.

199. World Organization for Animal Health [OIE]. OIE-WAHIS Portal: Animal health data. [cited 15 Jun 2021]. Available from: https://www.oie.int/en/animal-health-in-the-world/wahis-portal-animal-health-data/

200. Stefanovska J, Naletoski I, Nikolovski G, Kochevski Z, Živičnjak T, Martinković F. Prevalence of visceral leishmaniasis among urban dogs in Skopje, R. Macedonia. Second International Southeastern and Eastern Parasitological Society Conference. Zagreb, Croatia; 2011.

201. Cortes S, Vaz Y, Neves R, Maia C, Cardoso L, Campino L. Risk factors for canine leishmaniasis in an endemic Mediterranean region. Vet Parasitol. 2012;189: 189–196.

202. Carmona R. Leishmaniose canina: Comparação de resultados ELISA em diferentes grupos terapêuticos. PhD Thesis, Universidade de Évora. 2017.

203. Bráguez M. Leishmaniose em lobo ibérico – Estudo serológico. PhD Thesis, Instituto Politécnico de Viseu. 2014.

204. Marcos R, Santos M, Malhaõ F, Pereira R, Fernandes AC, Montenegro L, et al. Pancytopenia in a cat with visceral leishmaniasis. Vet Clin Pathol. 2009;38: 201–205.

205. Martins S, Vilares A, Ferreira I, Reis T, Gargaté MJ. Leishmaniase: confirmação laboratorial de casos clínicos suspeitos de infeção entre 2008 e 2013. Boletim Epidemiológico Observações. 2014;3(Supl 3): 23–25.

206. Santos Silva AF, Figueiredo Dias JPBC, Nuak JMNGS, Rocha Aguiar F, Araújo Pinto JA, Sarmento ACEM. Visceral leishmaniasis in a patient with systemic lupus erythematosus. IDCases. 2015;2: 102–105.

207. Araujo S. Estudo de babesiose e leishmaniose nos cães dos concelhos de Lamego, Tarouca e Peso da Régua, Portugal. PhD Thesis, Universidade de Lisboa. 2017.

208. Gama A, Elias J, Ribeiro AJ, Alegria N, Schallig HDFH, Silva F, et al. Cutaneous leishmaniosis in a horse from northern Portugal. Vet Parasitol. 2014;200: 189–192.

209. Pimenta P, Alves-Pimenta S, Barros J, Barbosa P, Rodrigues A, Pereira MJ, et al. Feline leishmaniosis in Portugal: 3 cases (year 2014). Vet Parasitol Reg Stud Rep. 2015;1–2: 65–69.

210. Viegas C, Requicha J, Albuquerque C, Sargo T, MacHado J, Dias I, et al. Tongue nodules in canine leishmaniosis a case report. Parasit Vectors. 2012;5: 120.

211. Cardoso L, Mendão C, Madeira De Carvalho L. Prevalence of *Dirofilaria immitis*, *Ehrlichia canis*, *Borrelia burgdorferi* sensu lato, *Anaplasma* spp. and *Leishmania infantum* in apparently healthy and CVBD-suspect dogs in Portugal - A national serological study. Parasit Vectors. 2012;5: 62.

212. Cruz C. Determinação da prevalência da infeção por *Leishmania infantum* em gatos errantes da região de Faro. PhD Thesis, Universidade Nova de Lisboa. 2017.

213. Mendonça J. Risco de introdução de novas espécies de *Leishmania* na região do Algarve. PhD Thesis, Universidade Nova de Lisboa. 2011.

214. Maia C, Ramos C, Coimbra M, Bastos F, Martins A, Pinto P, et al. Bacterial and protozoal agents of feline vector-borne diseases in domestic and stray cats from southern Portugal. Parasit Vectors. 2014;7: 115.

215. Maia C, Ramos C, Coimbra M, Cardoso L, Campino L. Prevalence of *Dirofilaria immitis* antigen and antibodies to Leishmania infantum in cats from southern Portugal. Parasitol Int. 2015;64: 154–156.

216. Maia C, Coimbra M, Ramos C, Cristóvão JM, Cardoso L, Campino L. Serological investigation of *Leishmania infantum*, *Dirofilaria immitis* and *Angiostrongylus vasorum* in dogs from southern Portugal. Parasit Vectors. 2015;8: 152.

217. Pereira A, Cristóvão JM, Vilhena H, Martins Â, Cachola P, Henriques J, et al. Antibody response to *Phlebotomus perniciosus* saliva in cats naturally exposed to phlebotomine sand flies is positively associated with *Leishmania* infection. Parasit Vectors. 2019;12: 128.

218. Ramos C. A importância da infecção por *Leishmania* spp. e *Dirofilaria immitis* em gatos na região de Olhão. PhD Thesis, Universidade de Lisboa. 2012.

219. Fernandes M. Leishmaniose canina. PhD Thesis, Universidade Lusófona de Humanidades e Tecnologias. 2018.

220. Dionísio MT, Dias A, Rodrigues F, Félix M, Estêvão MH. Paediatric visceral leishmaniasis: Experience of a paediatric referral center: 1990-2009. Acta Med Port. 2011;24: 399–404.

221. Guerra B. Frequência de hemoparasitas em cães e gatos referente à casuística da região de Leiria, no período comprendido entre janeiro de 2015 e novembro de 2018. PhD Thesis, Universidade de Lisboa. 2019.

222. Pires H. Estudo sero-epidemiológico da leishmaniose canina na sub-região do Pinhal Interior Sul. PhD Thesis, Universidade de Évora. 2011.

223. Silva A. Leishmaniose canina na zona do pinhal. PhD Thesis, Universidade de Lisboa. 2015.

224. Branco S, Alves-Pires C, Maia C, Cortes S, Cristovão JMS, Gonçalves L, et al. Entomological and ecological studies in a new potential zoonotic leishmaniasis focus in Torres Novas municipality, Central Region, Portugal. Acta Trop. 2013;125: 339–348.

225. Branco S. Estudo dos flebótomos (Diptera, Phlebotominae), vectores de Leishmania sp. no concelho de Torres Novas, Portugal. PhD Thesis, Universidade Nova de Lisboa. 2011.

226. Dias A. Leishmaniose canina: estudo de casos na Cova da Beira. PhD Thesis, Universidade de Trás-os-Montes e Alto Douro. 2017.

227. Reisinho A. Actualização da Prevalência de Leishmaniose Canina nos Concelhos de Setúbal e Palmela. PhD Thesis, Universidade Nova de Lisboa. 2010.

228. Antunes J, da Costa JB, Guimarães M, Vaz-Pinto I, Soares-Almeida L, Filipe P. Post-Kala-azar dermal leishmaniasis due to *Leishmania donovani* in Europe–case report. Int J Dermatol. 2013;52: 1584–1586.

229. Camara L, Queirós J, Ribeiro R, Teófilo E. Meglumine antimoniate combination treatment for relapsing Kala-azar after treatment and secondary prophylaxis failure with liposomal amphotericin B in two HIV-coinfected patients. BMJ Case Rep. 2019;12: e231929.

230. Caria J. Leishmaniose visceral e infeção por VIH em retrospetiva de 2000 a 2017 num hospital central de Lisboa. PhD Thesis, Universidade de Lisboa. 2018.

231. Fernandes M. Prevalência da infeccção por *Leishmania infantum* na população canina da região de Cascais. PhD Thesis, Universidade Lusófona de Humanidades e Tecnologias. 2013.

232. Fonseca M. Estudo da seroprevalência de Anticorpos Anti-*Leishmania* spp. numa população que coabita com canideos com leishmaniose. PhD Thesis, Universidade de Lisboa. 2010.

233. Gomes P. Deteção da infeção por *Leishmania* spp., em gatos da Área Metropolitana de Lisboa, através de técnicas de diagnóstico serológico (IFI e ELISA) e de uma técnica molecular (qPCR) aplicada a células conjuntivais e a sangue. PhD Thesis, Universidade de Lisboa. 2015.

234. Helhazar M. Avaliação do papel dos roedores das espécies *Mus musculus* e *Rattus norvegicus* como hospedeiros reservatórios de *Leishmania infantum* nos concelhos de Sesimbra e Sintra. PhD Thesis, Universidade de Lisboa. 2012.

235. Helhazar M, Leitão J, Duarte A, Tavares L, da Fonseca IP. Natural infection of synathropic rodent species *Mus musculus* and *Rattus norvegicus* by *Leishmania infantum* in Sesimbra and Sintra–Portugal. Parasit Vectors. 2013;6: 88.

236. Silva I. Estudo de hemoparasitas transmitidos por vectores, em cães de canil, Setúbal, Portugal. PhD Thesis, Universidade de Lisboa. 2011.

237. Tavares J. Caracterização epidemiológica de canídeos com diagnóstico de Leishmaniose. Hospital Veterinário do Restelo, Lisboa (2014 – 2016). PhD Thesis, Universidade de Évora. 2017.

238. Cristóvão J. Monitorização do risco de exposição à leishmaniose zoonótica. PhD Thesis, Universidade Nova de Lisboa. 2015.

239. Maia C, Alwassouf S, Cristóvão JM, Ayhan N, Pereira A, Charrel RN, et al. Serological association between *Leishmania infantum* and sand fly fever Sicilian (but not Toscana) virus in sheltered dogs from southern Portugal. Parasit Vectors. 2017;10: 92.

240. Maia C, Cristóvão J, Pereira A, Kostalova T, Lestinova T, Sumova P, et al. Monitoring *Leishmania* infection and exposure to *Phlebotomus perniciosus* using minimal and non-invasive canine samples. Parasit Vectors. 2020;13: 119.

241. Martins T. Detecção de *Ehrlichia* spp./*Anaplasma* spp., *Rickettsia* spp., *Mycoplasma haemofelis* e *Leishmania infantum* em felinos errantes e sua relação com a presença de retrovírus e com a sintomatologia manifestada. PhD Thesis, Universidade de Lisboa. 2012.

242. Moita M. Estudo retrospectivo das doenças transmitidas por vectores – Babesiose, Ehrlichiose, Rickettsiose e Leishmaniose – em cães no concelho de Cascais. PhD Thesis, Universidade Lusófona de Humanidades e Tecnologias. 2012.

243. Monteiro A. Estudo epidemiológico da Leishmaniose canina na zona da Arrábida. PhD Thesis, Universidade Lusófona de Humanidades e Tecnologias. 2010.

244. Pereira A, Valente J, Parreira R, Cristovão JM, Azinheira S, Campino L, et al. An Unusual Case of Feline Leishmaniosis With Involvement of the Mammary Glands. Top Companion Anim Med. 2019;37: 100356.

245. Pinto P. Prevalência da infeção por *Leishmania* sp. em gatos residentes no Concelho de Cascais. PhD Thesis, Universidade Lusófona de Humanidades e Tecnologias. 2013.

246. Santos J. Estudo observacional transversal de parasitas em cães errantes no concelho de Vila Franca de Xira, Portugal. PhD Thesis, Universidade de Lisboa. 2014.

247. Armés S. Rastreio de *Leishmania infantum* em cães assintomáticos na região de Mafra. PhD Thesis, Universidade de Lisboa. 2010.

248. Zamite C. Clínica de animais de companhia. PhD Thesis, Universidade de Évora. 2015.

249. Bastos F. Infecções causadas por protozoários e bactérias transmitidas por vectores em gatos residentes na região da Arrábida, Setúbal. PhD Thesis, Universidade Lusófona de Humanidades e Tecnologias. 2013.

250. Martinho Almeida BF. Deteção molecular de protozoários e bactérias transmitidos por artrópodes vetores em cães do sul de Portugal. PhD Thesis, Universidade Lusófona de Humanidades e Tecnologias. 2013.

251. Canhoto R. Estudo dos fatores de risco associados à infeção por *Anaplasma* spp., *Ehrlichia* spp., *Babesia* spp., *Leishmania* spp. e *Dirofilaria* spp. em cães do Alentejo Norte, Portugal. PhD Thesis, Universidade de Lisboa. 2020.

252. Lopes L, Vasconcelos P, Borges-Costa J, Soares-Almeida L, Campino L, Filipe P. An atypical case of cutaneous leishmaniasis caused by Leishmania infantum in Portugal. Dermatol Online J. 2013;19:20407.

253. Paulino N. Frequência de doenças caninas transmitidas por vetores num abrigo no concelho de Moura, Alentejo. PhD Thesis, Universidade Lusófona de Humanidades e Tecnologias. 2018.

254. Schallig HDFH, Cardoso L, Semião-Santos SJ. Seroepidemiology of canine leishmaniosis in Évora (southern Portugal): 20-year trends. Parasit Vectors. 2013;6: 100.

255. Cimpan AA, Diakou A, Papadopoulos I, Miron LD. Serological study of exposure to *Leishmania* in dogs living in shelters, in South-East Romania. Rev Rom Med Vet. 2019;29: 54–58.

256. Cazan CD, Ionică AM, Matei IA, D’Amico G, Muñoz C, Berriatua E, et al. Detection of *Leishmania infantum* DNA and antibodies against *Anaplasma* spp., *Borrelia* *burgdorferi* s.l. and *Ehrlichia canis* in a dog kennel in South-Central Romania. Acta Vet Scand. 2020;62: 42.

257. Mitková B, Hrazdilová K, D’Amico G, Duscher GG, Suchentrunk F, Forejtek P, et al. Eurasian golden jackal as host of canine vector-borne protists. Parasit Vectors. 2017;10: 183.

258. Dumitrache MO, Nachum-Biala Y, Gilad M, Mircean V, Cazan CD, Mihalca AD, et al. The quest for canine leishmaniasis in Romania: The presence of an autochthonous focus with subclinical infections in an area where disease occurred. Parasit Vectors. 2016;9: 297.

259. Salvatore D, Di Francesco A, Parigi M, Poglayen G, Battistini M, Baldelli R. Canine leishmaniasis surveillance program in a San Marino Republic Kennel. Vet Ital. 2013;49: 341–346.

260. Ćirović D, Chochlakis D, Tomanović S, Sukara R, Penezić A, Tselentis Y, et al. Presence of *Leishmania* and *Brucella* species in the golden jackal *Canis aureus* in Serbia. Biomed Res Int. 2014;2014: 728516.

261. Savić S, Vidić B, Grgić Z, Potkonjak A, Spasojevic L. Emerging vector-borne diseases - Incidence through vectors. Front Public Health. 2014;2:267.

262. Dakic ZD, Pelemis MR, Stevanovic GD, Poluga JL, Lavadinovic LS, Milosevic IS, et al. Epidemiology and diagnostics of visceral leishmaniasis in Serbia. Clin Microbiol Infect. 2009;15: 1173–1176.

263. Kotnik T. Dog leishmaniasis in Slovenia: A probable creation of the first enzootic focus-a case report. Vet Arh. 2020;90: 317–322.

264. Díaz-Regañón D, Roura X, Suárez ML, León M, Sainz Á. Serological evaluation of selected vector-borne pathogens in owned dogs from northern Spain based on a multicenter study using a commercial test. Parasit Vectors. 2020;13: 301.

265. Gálvez R, Montoya A, Cruz I, Fernández C, Martín O, Checa R, et al. Latest trends in *Leishmania infantum* infection in dogs in Spain, Part I: Mapped seroprevalence and sand fly distributions. Parasit Vectors. 2020;13: 204.

266. López-Peña M, Alemã N, Mũñoz F, Fondevila D, Suárez ML, Goicoa A, et al. Visceral leishmaniasis with cardiac involvement in a dog: A case report. Acta Vet Scand. 2009;51:20.

267. Miró G, Checa R, Montoya A, Hernández L, Dado D, Gálvez R. Current situation of *Leishmania infantum* infection in shelter dogs in northern Spain. Parasit Vectors. 2012;5: 60.

268. Herrador Z, Gherasim A, Jimenez BC, Granados M, San Martín JV, Aparicio P. Epidemiological changes in leishmaniasis in Spain according to hospitalization-based records, 1997-2011: raising awareness towards leishmaniasis in non-HIV patients. PLoS Negl Trop Dis. 2015;9: e0003594.

269. Oleaga A, Vicente J, Ferroglio E, de Macedo MR, Casais R, del Cerro A, et al. Concomitance and interactions of pathogens in the Iberian wolf (*Canis lupus*). Res Vet Sci. 2015;101: 22–27.

270. Oleaga A, Zanet S, Espí A, de Macedo MR, Gortázar C, Ferroglio E. *Leishmania* in wolves in northern Spain: A spreading zoonosis evidenced by wildlife sanitary surveillance. Vet Parasitol. 2018;255: 26–31.

271. López-Giménez MT, Sánchez Salas P, Lahuerta AA, López-Borrachina R, Oncins Torres R, Griabal García M. Leishmaniasis, an emergent disease. Five clinical cases presentation. Med Cutan Ibero Lat Am. 2011;39: 13–18.

272. Gómez-Ochoa P, Castillo JA, Gascón M, Zarate JJ, Alvarez F, Couto CG. Use of domperidone in the treatment of canine visceral leishmaniasis: A clinical trial. Vet J. 2009;179: 259–263.

273. Villanueva-Saz S, Basurco A, Martín V, Fernández A, Loste A, Verde MT. Comparison of a qualitative immunochromatographic test with two quantitative serological assays for the detection of antibodies to *Leishmania infantum* in dogs. Acta Vet Scand. 2019;61: 38.

274. Abellán-Martínez J, Guerra-Vales JM, Fernández-Cotarelo MJ, González-Alegre MT. Evolution of the incidence and aetiology of fever of unknown origin (FUO), and survival in HIV-infected patients after HAART (Highly Active Antiretroviral Therapy). Eur J Int Med. 2009;20: 474–477.

275. Aguado M, Espinosa P, Romero-Maté A, Tardío JC, Córdoba S, Borbujo J. Outbreak of cutaneous leishmaniasis in Fuenlabrada, Madrid. Actas Dermosifiliogr. 2013;104: 334–342.

276. Arce A, Estirado A, Ordobas M, Sevilla S, García N, Moratilla L, et al. Re-emergence of Leishmaniasis in Spain: Community outbreak in Madrid, Spain, 2009 to 2012. Euro Surveill. 2013;18: 20546.

277. Ayllon T, Iniz PPVP, Breitschwerdt EB, Villaescusa A, Rodriguez-Franco F, Sainz A. Vector-Borne Diseases in Client-Owned and Stray Cats from Madrid, Spain. Vector Borne Zoonotic Dis. 2012;12: 143–150.

278. Bautista G, Ramos A, Gil S. Visceral leishmaniasis in hematopoietic stem cell transplantation. Transpl Int. 2012;25: e83–e85.

279. Blázquez-Gamero D, Domínguez-Pinilla N, Chicharro C, Negreira S, Galán P, Pérez-Gorricho B, et al. Hemophagocytic Lymphohistiocytosis in Children with Visceral Leishmaniasis. Pediatr Infect Dis J. 2015;34: 667–669.

280. Carrasco-Antón N, López-Medrano F, Fernández-Ruiz M, Carrillo E, Moreno J, García-Reyne A, et al. Environmental factors as key determinants for visceral leishmaniasis in solid organ transplant recipients, Madrid, Spain. Emerg Infect Dis. 2017;23: 1155–1159.

281. Chicharro C, Llanes-Acevedo P, García E, Nieto J, Moreno J, Cruz I. Molecular typing of *Leishmania infantum* isolates from a leishmaniasis outbreak in Madrid, Spain, 2009 to 2012. Euro Surveill. 2013;18: 20545.

282. Chiverto Llamazares Y, Cabezas López E, Castro Sánchez M, Iglesias Goy E. Visceral leishmaniasis as a diagnosis of puerperal fever of unknown origin. Prog Obstet Ginecol. 2014;57: 247–250.

283. Córdoba S, Gandolfo Cano M, Aguado M, Huerta-Brogera M, Romero A, Martínez-Morán C, et al. Delayed allergic skin reactions due to intralesional meglumine antimoniate therapy for cutaneous leishmaniasis. Allergy: Eur J Allergy Clin Immunol. 2012;67: 1609–1611.

284. Daza González MA, Miró G, Fermín Rodríguez M, Rupérez Noguer C, Fragío Arnold C. Short term impacts of meglumine antimoniate treatment on kidney function in dogs with clinical leishmaniosis. Res Vet Sci. 2019;126: 131–138.

285. de la Cruz ML, Pérez A, Domínguez M, Moreno I, García N, Martínez I, et al. Assessment of the sensitivity and specificity of serological (IFAT) and molecular (direct-PCR) techniques for diagnosis of leishmaniasis in lagomorphs using a Bayesian approach. Vet Med Sci. 2016;2: 211–220.

286. Del Rosal Rabes T, Baquero-Artigao F, Gómez Fernández C, García Miguel MJ, De Lucas Laguna R. Treatment of cutaneous leishmaniasis with liposomal amphotericin B. An Pediatr. 2010;73: 101–102.

287. del Rosal T, Artigao FB, Miguel MJG, de Lucas R, del Castillo F. Successful treatment of childhood cutaneous leishmaniasis with liposomal amphotericin B: report of two cases. J Trop Pediatr. 2010;56: 122–124.

288. Gálvez R, Miró G, Descalzo MA, Nieto J, Dado D, Martín O, et al. Emerging trends in the seroprevalence of canine leishmaniosis in the Madrid region (central Spain). Vet Parasitol. 2010;169: 327–334.

289. García N, Moreno I, Alvarez J, de la Cruz ML, Navarro A, Pérez-Sancho M, et al. Evidence of *Leishmania infantum* infection in rabbits (*Oryctolagus cuniculus*) in a natural area in Madrid, Spain. Biomed Res Int. 2014;2014: 318254.

290. García-Río I, Daudén E, Ballestero-Díez M, Fraga J, García-Díez A. Leishmaniasis and Rheumatoid Nodulosis in a Patient with HIV Infection. Actas Dermosifiliogr. 2010;101: 164–167.

291. Gimeno Aránguez M, Aramendi T, Rivera Sanchez T, Casado Fariñas I, Tortoledo Bruni A. Isolated cervical “Piringer-Kuchinka-like” lymphadenitis due to leishmaniasis in an immunocompetent patient. Rev Esp Patol. 2012;45: 35–39.

292. Gomez-Barroso D, Herrador Z, San Martín JV, Gherasim A, Aguado M, Romero-Maté A, et al. Spatial distribution and cluster analysis of a leishmaniasis outbreak in the south-western Madrid region, Spain, September 2009 to April 2013. Euro Surveill. 2015;20: 11–20.

293. González E, Jiménez M, Hernández S, Martín-Martín I, Molina R. Phlebotomine sand fly survey in the focus of leishmaniasis in Madrid, Spain (2012-2014): Seasonal dynamics, *Leishmania infantum* infection rates and blood meal preferences. Parasit Vectors. 2017;10: 368.

294. González E, Molina R, Aldea I, Iriso A, Tello A, Jiménez M. *Leishmania* sp. detection and blood-feeding behaviour of *Sergentomyia minuta* collected in the human leishmaniasis focus of southwestern Madrid, Spain (2012–2017). Transboundd Emerg Dis. 2020;67: 1393–1400.

295. González Martínez M, Mariño Martínez C, Baldominos Utrilla G, Fernández Martínez MN. Analysis of the use of liposomal amphotericin B. Rev Iberoam Micol. 2014;31: 109–113.

296. Horrillo L, San Martín JV, Molina L, Madroñal E, Matía B, Castro A, et al. Atypical presentation in adults in the largest community outbreak of leishmaniasis in Europe (Fuenlabrada, Spain). Clin Microbiol Infect. 2015;21: 269–273.

297. Horrillo L, Castro A, Matía B, Molina L, García-Martínez J, Jaqueti J, et al. Clinical aspects of visceral leishmaniasis caused by *L. infantum* in adults. Ten years of experience of the largest outbreak in Europe: what have we learned? Parasit Vectors. 2019;12: 359.

298. Ibarra-Meneses AV, Carrillo E, Nieto J, Sánchez C, Ortega S, Estirado A, et al. Prevalence of asymptomatic *Leishmania* infection and associated risk factors, after an outbreak in the Southwestern Madrid Region, Spain, 2015. Euro Surveill. 2019;24: 1800379.

299. Jiménez M, González E, Iriso A, Marco E, Alegret A, Fúster F, et al. Detection of *Leishmania infantum* and identification of blood meals in *Phlebotomus perniciosus* from a focus of human leishmaniasis in Madrid, Spain. Parasitol Res. 2013;112: 2453–2459.

300. Jiménez M, González E, Martín-Martín I, Hernández S, Molina R. Could wild rabbits (*Oryctolagus cuniculus*) be reservoirs for *Leishmania infantum* in the focus of Madrid, Spain? Vet Parasitol. 2014;202: 296–300.

301. Miró G, Gálvez R, Fraile C, Descalzo MA, Molina R. Infectivity to *Phlebotomus perniciosus* of dogs naturally parasitized with *Leishmania infantum* after different treatments. Parasit Vectors. 2011;4: 52.

302. Miró G, Rupérez C, Checa R, Gálvez R, Hernández L, García M, et al. Current status of *L. infantum* infection in stray cats in the Madrid region (Spain): implications for the recent outbreak of human leishmaniosis? Parasit Vectors. 2014;7: 112.

303. Miró G, Müller A, Montoya A, Checa R, Marino V, Marino E, et al. Epidemiological role of dogs since the human leishmaniosis outbreak in Madrid. Parasit Vectors. 2017;10: 209.

304. Miró G, Troyano A, Montoya A, Fariñas F, Fermín ML, Flores L, et al. First report of *Leishmania infantum* infection in the endangered orangutan (*Pongo pygmaeus pygmaeus*) in Madrid, Spain. Parasit Vectors. 2018;11: 185.

305. Molina R, Jiménez M, García-Martínez J, San Martín JV, Carrillo E, Sánchez C, et al. Role of asymptomatic and symptomatic humans as reservoirs of visceral leishmaniasis in a mediterranean context. PLoS Negl Trop Dis. 2020;14: e0008253.

306. Montoya A, de Quadros LP, Mateo M, Hernández L, Gálvez R, Alcántara G, et al. *Leishmania infantum* infection in Bennett’s wallabies (*Macropus rufogriseus rufogriseus*) in a Spanish wildlife park. J Zoo Wildl Med. 2016;47: 586–593.

307. Montoya A, García M, Gálvez R, Checa R, Marino V, Sarquis J, et al. Implications of zoonotic and vector-borne parasites to free-roaming cats in central Spain. Vet Parasitol. 2018;251: 125–130.

308. Moreno I, Álvarez J, García N, de la Fuente S, Martínez I, Marino E, et al. Detection of anti-*Leishmania infantum* antibodies in sylvatic lagomorphs from an epidemic area of Madrid using the indirect immunofluorescence antibody test. Vet Parasitol. 2014;199: 264–267.

309. Ortega MV, Moreno I, Domínguez M, de la Cruz ML, Martín AB, Rodríguez-Bertos A, et al. Application of a specific quantitative real-time PCR (qPCR) to identify *Leishmania infantum* DNA in spleen, skin and hair samples of wild Leporidae. Vet Parasitol. 2017;243: 92–99.

310. Ortega-García MV, Salguero FJ, Rodríguez-Bertos A, Moreno I, García N, García-Seco T, et al. A pathological study of *Leishmania infantum* natural infection in European rabbits (*Oryctolagus cuniculus*) and Iberian hares (*Lepus granatensis*). Transbound Emerg Dis. 2019;66: 2474–2481.

311. Ortiz M, Mon C, Herrero JC, Oliet A, Rodríguez I, Ortega O, et al. Glomerulonephritis and cryoglobulinemia: First manifestation of visceral leishmaniasis. Clin Nephrol. 2015;83: 370–377.

312. Parody N, Cacheiro-Llaguno C, Osuna C, Renshaw-Calderón A, Alonso C, Carnés J. Circulating immune complexes levels correlate with the progression of canine leishmaniosis in naturally infected dogs. Vet Parasitol. 2019;274: 108921.

313. Pérez-Jacoiste Asín MA, Carrasco-Antón N, Fernández-Ruiz M, San Juan R, Alonso-Moralejo R, González E, et al. Experience with miltefosine for persistent or relapsing visceral leishmaniasis in solid organ transplant recipients: A case series from Spain. Transpl Infect Dis. 2017;19: e12623.

314. Prieto Tato LM, La Orden Izquierdo E, Guillén Martn S, Salcedo Lobato E, García Esteban C, Garca-Bermejo I, et al. Visceral childhood leishmaniasis: Diagnosis and treatment. An Pediatr. 2010;72: 347–351.

315. Ramírez GA, Peñafiel-Verdú C, Altimira J, García-González B, Vilafranca M. Naturally Acquired Visceral Leishmaniosis in a Captive Bennett’s Wallaby (*Macropus rufogriseus rufogriseus*). Vet Pathol. 2013;50: 188–190.

316. Rojas Mora E, Garrido Ríos A, Echeverría García B, Borbujo J. An Unusual Presentation of Cutaneous Leishmaniasis: The Role of Skin Ultrasound. Actas Dermosifiliogr. 2019;110: 171–174.

317. Romero-Maté A, Martínez-Sánchez D, Tardío JC, Moreno-Torres A, García-Donoso C, Aguado-Lobo M, et al. Cutaneous leishmaniasis with histopathologic pattern of non-necrotizing granulomatous dermatitis in patients treated with adalimumab. Dermatol Online J. 2012;18: 7.

318. Ruiz Martin I, Mejia Nieto M, Lopez Fernandez P, Zubillaga Rodriguez I. Mucocutaneous leishmaniasis in an immunocompetent patient: A case report. Rev Esp Cir Oral Maxilofac. 2018;40: 41–43.

319. Freites-Martinez A, Córdoba S, Bermejo A, Borbujo J. Mucocutaneous leishmaniasis caused by *Leishmania infantum* var Lombardi in an immunocompetent patient, Spain. Enferm Infecc Microbiol Clin Monogr. 2015;33: 499–500.

320. Molina R, Jiménez MI, Cruz I, Iriso A, Martín-Martín I, Sevillano O, et al. The hare (*Lepus granatensis*) as potential sylvatic reservoir of *Leishmania infantum* in Spain. Vet Parasitol. 2012;190: 268–271.

321. Lledó L, Giménez-Pardo C, Saz JV, Serrano JL. Wild Red Foxes (*Vulpes vulpes*) as Sentinels of Parasitic Diseases in the Province of Soria, Northern Spain. Vector Borne Zoonotic Dis. 2015;15: 743–749.

322. Couto CG, Lorentzen L, Beall MJ, Shields J, Bertolone N, Couto JI, et al. Serological study of selected vector-borne diseases in shelter dogs in central Spain using point-of-care assays. Vector Borne Zoonotic Dis. 2010;10: 885–888.

323. Del Olmo Martínez L, Aller De La Fuente R, Velayos Jiménez B, Fernández Salazar L, González Hernández JM. Visceral leishmaniasis diagnosed by duodenal biopsy. Rev Esp Enferm Dig. 2009;101: 439–440.

324. Mendoza Chaparro C, Martín Ayuso M, García-Arpa M, Romero Aguilera G. Cutaneous leishmaniasis by mucosal inoculation treated with oral miltefosine. Piel. 2016;31: 657–659.

325. Rodríguez Vidigal FF, Calvo Cano A, Sánchez Sánchez M, Nogales Muñoz N, Vera Tomé A, Muñoz Sanz A. Haemophagocytic syndrome associated with infections: Not so uncommon. Rev Clin Esp. 2020;220: 109–114.

326. Alarcon I, Carrera C, Puig S, Malvehy J. In vivo confocal microscopy features of cutaneous leishmaniasis. Dermatology. 2014;228: 121–124.

327. Baltà-Cruz S, Alsina-Gibert M, Mozos-Rocafort A, Cervera C, Colomo-Saperas L, Del Río A, et al. Pseudolymphomatoid cutaneous leishmaniasis in a patient treated with adalimumab for rheumatoid arthritis. Acta Derm Venereol. 2009;89: 432–433.

328. Català A, Barnadas MA, Muñoz C, Puig L. Cutaneous leishmaniasis in a patient receiving infliximab for psoriatic arthritis: Treatment with cryotherapy and intralesional meglumine antimonate. Actas Dermosifiliogr. 2014;105: 714–716.

329. Galán-Puchades MT, Gómez-Samblás M, Suárez-Morán JM, Osuna A, Sanxis-Furió J, Pascual J, et al. Leishmaniasis in Norway rats in Sewers, Barcelona, Spain. Emerg Infect Dis. 2019;25: 1222–1224.

330. Giavedoni P, Iranzo P, Fuertes I, Estrach T, Alsina Gibert M. Cutaneous leishmaniasis: 20 years’ experience in a Spanish tertiary care hospital. Actas Dermosifiliogr. 2015;106: 310–316.

331. Guillén MC, Alcover MM, Borruel N, Sulleiro E, Salvador F, Berenguer D, et al. *Leishmania infantum* asymptomatic infection in inflammatory bowel disease patients under anti-TNF therapy. Heliyon. 2020;6: e03940.

332. Herrerias-Moreno J, Expósito-Serrano V, Agut-Busquet E, Corbacho M, Sáez E, Luelmo J. Cutaneous leishmaniasis mimicking dactylitis in a patient with rheumatoid arthritis treated with certolizumab. Clin Exp Dermatol. 2018;43: 348–350.

333. José-López R, de la Fuente C, Pumarola M, Añor S. Intramedullary spinal cord mass presumptively associated with leishmaniasis in a dog. J Am Vet Med Assoc. 2014;244: 200–204.

334. Marcoval J, Penín RM. Evolution of cutaneous leishmaniasis in the last 30 years in a tertiary hospital of the European Mediterranean coast. Int J Dermatol. 2017;56: 750–753.

335. Martinez De Narvajas I, Díaz A, Bassegoda O, Carpio A, Fuster C, Valls ME, et al. Acute liver failure due to visceral leishmaniasis in Barcelona: A case report. BMC Infect Dis. 2019;19: 874.

336. Millán J. Molecular investigation of vector-borne parasites in wild micromammals, Barcelona (Spain). Parasitol Res. 2018;117: 3015–3018.

337. Molina I, Fisa R, Riera C, Falco V, Elizalde A, Salvador F, et al. Ultrasensitive real-time PCR for the clinical management of visceral leishmaniasis in HIV-infected patients. Am J Trop Med Hyg. 2013;89: 105–110.

338. Moltó A, Mateo L, Lloveras N, Olivé A, Minguez S. Visceral leishmaniasis and macrophagic activation syndrome in a patient with rheumatoid arthritis under treatment with adalimumab. Joint Bone Spine. 2010;77: 271–273.

339. Naranjo C, Fondevila D, Leiva M, Roura X, Peña T. Detection of *Leishmania* spp. and associated inflammation in ocular-associated smooth and striated muscles in dogs with patent leishmaniosis. Vet Ophthalmol. 2010;13: 139–143.

340. Naranjo C, Fondevila D, Altet L, Francino O, Ríos J, Roura X, et al. Evaluation of the presence of *Leishmania* spp. by real-time PCR in the lacrimal glands of dogs with leishmaniosis. Vet J. 2012;193: 168–173.

341. Reina D, Cerdà D, Güell E, Martínez Montauti J, Pineda A, Corominas H. Visceral leishmaniasis in a rheumatoid arthritis patient receiving methotrexate. Reumatol Clin. 2017;13: 354–356.

342. Tabar MD, Francino O, Altet L, Sánchez A, Ferrer L, Roura X. PCR survey of vector-borne pathogens in dogs living in and around Barcelona, an area endemic for leishmaniosis. Vet Rec. 2009;164: 112–116.

343. Todolí F, Solano-Gallego L, Ojeda A, Quintana J, Lloret A, Roura X, et al. Anti-*Leishmania* IgA in urine samples from dogs with clinical leishmaniasis. Veterinary Parasitology. 2009;159: 17–23.

344. Torres M, Bardagí M, Roura X, Zanna G, Ravera I, Ferrer L. Long term follow-up of dogs diagnosed with leishmaniosis (clinical stage II) and treated with meglumine antimoniate and allopurinol. Vet J. 2011;188: 346–351.

345. Velez R, Ballart C, Domenech E, Abras A, Fernández-Arévalo A, Gómez SA, et al. Seroprevalence of canine *Leishmania infantum* infection in the Mediterranean region and identification of risk factors: The example of North-Eastern and Pyrenean areas of Spain. Prev Vet Med. 2019;162: 67–75.

346. Velez R, Domenech E, Cairó J, Gállego M. The impact of canine leishmaniosis vaccination with Canileish® in *Leishmania infantum* infection seroprevalence studies. Acta Trop. 2020;202: 105259.

347. Alcover MM, Gramiccia M, Di Muccio T, Ballart C, Castillejo S, Picado A, et al. Application of molecular techniques in the study of natural infection of *Leishmania infantum* vectors and utility of sandfly blood meal digestion for epidemiological surveys of leishmaniasis. Parasitol Res. 2012;111: 515–523.

348. Ballart C, Alcover MM, Portús M, Gállego M. Is leishmaniasis widespread in Spain? First data on canine leishmaniasis in the province of Lleida, Catalonia, northeast Spain. Trans R Soc Trop Med Hyg. 2012;106: 134–136.

349. Ballart C, Alcover MM, Picado A, Nieto J, Castillejo S, Portús M, et al. First survey on canine leishmaniasis in a non classical area of the disease in Spain (Lleida, Catalonia) based on a veterinary questionnaire and a cross-sectional study. Prev Vet Med. 2013;109: 116–127.

350. Ruiz Ruiz S, Tasias Pitarch M, Delegido Sánchez-Migallón A, Pedrol Clotet E. Relapses of leishmaniasis in an HIV infected patient: A therapeutic challenge. Enferm Infecc Microbiol Clin. 2012;30: 48–49.

351. Alonso F, Giménez Font P, Manchón M, Ruiz De Ybáñez R, Segovia M, Berriatua E. Geographical variation and factors associated to seroprevalence of canine leishmaniosis in an endemic mediterranean area. Zoonoses Public Health. 2010;57: 318–328.

352. Ena J, Pasquau F, Del Mar LόPez-Perezagua M, Martinez-Peinado C, Arjona F. Screening for subclinical *Leishmania* infection in HIV-infected patients living in eastern Spain. Pathog Glob Health. 2014;108: 356–361.

353. López Corbalán JC, Seguí Ripoll JM, Cuadrado Pastor JM. Treatment of visceral leishmaniasis with miltefosine in three cases with HIV infection. Atención Farmacéutica. 2011;13: 306–308.

354. Ramos JM, León R, Merino E, Montero M, Aljibe A, Blanes M, et al. Is visceral leishmaniasis different in immunocompromised patients without human immunodeficiency virus? A comparative, multicenter retrospective cohort analysis. Am J Trop Med Hyg. 2017;97: 1127–1133.

355. Ramos JM, Clavijo A, Moral L, Gavilan C, Salvador T, de Dios J. Epidemiological and clinical features of visceral leishmaniasis in children in Alicante Province, Spain. Paediatr Int Child Health. 2018;38: 203–208.

356. Gironé G, Mateo C, Gaya V, Usó J, Mínguez C, Roca B, et al. Admissions for imported and non-imported parasitic diseases at a General Hospital in Spain: A retrospective analysis. Travel Med Infect Dis. 2015;13: 322–328.

357. García Callejo FJ, Balaguer García R, Juantegui Azpilicueta M, García Aguayo JM. ENT involvement in *Leishmania* infections. Acta Otorrinolaringol Esp. 2021;72: 3–10.

358. Garrido-Jareño M, Sahuquillo-Torralba A, Chouman-Arcas R, Castro-Hernández I, Molina-Moreno JM, Llavador-Ros M, et al. Cutaneous and mucocutaneous leishmaniasis: Experience of a Mediterranean hospital. Parasit Vectors. 2020;13: 24.

359. Giner J, Basurco A, Alcover MM, Riera C, Fisa R, López RA, et al. First report on natural infection with *Leishmania infantum* in a domestic ferret (*Mustela putorius furo*) in Spain. Vet Parasitol Reg Stud Rep. 2020;19: 100369.

360. Giner J, Villanueva-Saz S, Alcover MM, Riera C, Fisa R, Basurco A, et al. Treatment and follow-up of a domestic ferret (*Mustela putorius furo*) with clinical leishmaniosis caused by *Leishmania infantum*. Vet Parasitol Reg Stud Rep. 2020;21: 100423.

361. Monzó-Gandía R, García-Callejo FJ, Calatayud-Blas AM, Calvo-González J. Laryngeal leishmaniasis. A case report. Acta Otorrinolaringol Esp. 2014;65: 194–196.

362. Roth-Damas P, Sempere-Manuel M, Mialaret-Lahiguera A, Fernández-García C, Gil-Tomás JJ, Colomina-Rodríguez J, et al. Community outbreak of cutaneous leishmaniasis in La Ribera region of Valencia, Spain: Public Health measures. Enferm Infecc Microbiol Clin. 2017;35: 338–343.

363. Sabaté D, Llinás J, Homedes J, Sust M, Ferrer L. A single-centre, open-label, controlled, randomized clinical trial to assess the preventive efficacy of a domperidone-based treatment programme against clinical canine leishmaniasis in a high prevalence area. Prev Vet Med. 2014;115: 56–63.

364. Sherry K, Miró G, Trotta M, Miranda C, Montoya A, Espinosa C, et al. A serological and molecular study of *Leishmania infantum* infection in cats from the island of Ibiza (Spain). Vector Borne Zoonotic Dis. 2011;11: 239–245.

365. Alcover MM, Ballart C, Serra T, Castells X, Scalone A, Castillejo S, et al. Temporal trends in canine leishmaniosis in the Balearic Islands (Spain): A veterinary questionnaire. Prospective canine leishmaniosis survey and entomological studies conducted on the Island of Minorca, 20 years after first data were obtained. Acta Trop. 2013;128: 642–651.

366. Alcover MM, Rocamora V, Guillén MC, Berenguer D, Cuadrado M, Riera C, et al. Case report: Diffuse cutaneous leishmaniasis by *Leishmania infantum* in a patient undergoing immunosuppressive therapy: Risk status in an endemic mediterranean area. Am J Trop Med Hyg. 2018;98: 1313–1316.

367. Burnham AC, Ordeix L, Alcover MM, Martínez-Orellana P, Montserrat-Sangrà S, Willen L, et al. Exploring the relationship between susceptibility to canine leishmaniosis and anti-*Phlebotomus perniciosus* saliva antibodies in Ibizan hounds and dogs of other breeds in Mallorca, Spain. Parasit Vectors. 2020;13: 129.

368. Cabezón O, Millán J, Gomis M, Dubey JP, Ferroglio E, Almería S. Kennel dogs as sentinels of *Leishmania infantum*, *Toxoplasma gondii*, and *Neospora caninum* in Majorca Island, Spain. Parasitol Res. 2010;107: 1505–1508.

369. Hervás JA, Martín-Santiago A, Hervás D, Rojo E, Mena A, Rocamora V, et al. Old world *Leishmania infantum* cutaneous leishmaniasis unresponsive to liposomal amphotericin B treated with topical imiquimod. Pediatr Infect Dis J. 2012;31: 97–100.

370. Millán J, Zanet S, Gomis M, Trisciuoglio A, Negre N, Ferroglio E. An Investigation into Alternative Reservoirs of Canine Leishmaniasis on the Endemic Island of Mallorca (Spain). Transbound Emerg Dis. 2011;58: 352–357.

371. Pujol A, Fisa R, Riera C, Rocamora V, Boteller D. Diagnosis of cutaneous leishmaniosis. Value of a polymerase chain reaction technique for *Leishmania infantum* detection in samples collected in filter paper versus histological and immunohistochemical methods. Piel. 2012;27: 527–531.

372. Benito Navarro JR, Santaella Guardiola OM, Delgado Alvarez JF, Josephine Frandsen A. Laryngeal leishmaniasis as a differential diagnosis of glottic leukoplakia. Acta Otorrinolaringol Esp. 2013;64: 440–441.

373. Aliaga L, Ceballos J, Sampedro A, Cobo F, López-Nevot MÁ, Merino-Espinosa G, et al. Asymptomatic *Leishmania* infection in blood donors from the Southern of Spain. Infection. 2019;47: 739–747.

374. Blasco Morente G, Rodríguez-Granger J, Tercedor Sánchez J, Latorre Fuentes JM. Old world leishmaniasis on the ear lobe: A rare site. Actas Dermosifiliogr. 2014;105: 628–630.

375. Cobo F, Rodríguez-Granger J, Gómez-Camarasa C, Sampedro A, Aliaga–Martínez L, Navarro JM, et al. Localized mucosal leishmaniasis caused by *Leishmania infantum* mimicking cancer in the rhinolaryngeal region. Int J Infect Dis. 2016;50: 54–56.

376. Deutor Garcia L, Fraile Ruiz L, Cariati P. Isolated oral mucosa leishmaniasis: An unusual manifestation. Med Clin. 2019;152: e1–e2.

377. Díaz Sáez V, Morillas-Márquez F, Merino-Espinosa G, Corpas-López V, Morales-Yuste M, Pesson B, et al. *Phlebotomus langeroni* Nitzulescu (Diptera, Psychodidae) a new vector for *Leishmania infantum* in Europe. Parasitol Res. 2018;117: 1105–1113.

378. Díaz-Sáez V, Merino-Espinosa G, Morales-Yuste M, Corpas-López V, Pratlong F, Morillas-Márquez F, et al. High rates of *Leishmania infantum* and *Trypanosoma nabiasi* infection in wild rabbits (*Oryctolagus cuniculus*) in sympatric and syntrophic conditions in an endemic canine leishmaniasis area: Epidemiological consequences. Vet Parasitol. 2014;202: 119–127.

379. Elmahallawy EK, Cuadros-Moronta E, Liébana-Martos MC, Rodríguez-Granger JM, Sampedro-Martínez A, Agil A, et al. Seroprevalence of *Leishmania* infection among asymptomatic renal transplant recipients from southern Spain. Transpl Infect Dis. 2015;17: 795–799.

380. Márquez M, Pedregosa JR, López J, Marco-Salazar P, Fondevila D, Pumarola M. *Leishmania* amastigotes in the central nervous system of a naturally infected dog. J Vet Diagn Invest. 2013;25: 142–146.

381. Martín-Sánchez J, Morales-Yuste M, Acedo-Sánchez C, Barón S, Díaz V, Morillas-Márquez F. Canine leishmaniasis in Southeastern Spain. Emerg Infect Dis. 2009;15: 795–798.

382. Martín-Sánchez J, Rodríguez-Granger J, Morillas-Márquez F, Merino-Espinosa G, Sampedro A, Aliaga L, et al. Leishmaniasis due to *Leishmania infantum*: Integration of human, animal and environmental data through a One Health approach. Transbound Emerg Dis. 2020;67: 2423–2434.

383. Morales-Yuste M, Morillas-Márquez F, Díaz-Sáez V, Barón-López S, Acedo-Sánchez C, Martín-Sánchez J. Epidemiological implications of the use of various methods for the diagnosis of canine leishmaniasis in dogs with different characteristics and in differing prevalence scenarios. Parasitol Res. 2012;111: 155–164.

384. Navea-Pérez HM, Díaz-Sáez V, Corpas-López V, Merino-Espinosa G, Morillas-Márquez F, Martín-Sánchez J*. Leishmania infantum* in wild rodents: reservoirs or just irrelevant incidental hosts? Parasitol Res. 2015;114: 2363–2370.

385. Vargas-Hitos Dr JA, Roa-Chamorro R, Sabio JM, Javier-Martínez R, Martín-Castro A, Jiménez-Alonso J. Leishmaniasis, lupus nephritis and immunosuppressive therapy. QJM. 2016;109: 51–52.

386. Chitimia L, Muñoz-García CI, Sánchez-Velasco D, Lizana V, del Río L, Murcia L, et al. Cryptic Leishmaniosis by *Leishmania infantum*, a feature of canines only? A study of natural infection in wild rabbits, humans and dogs in southeastern Spain. Vet Parasitol. 2011;181: 12–16.

387. Goyena E, Pérez-Cutillas P, Chitimia L, Risueño J, García-Martínez JDD, Bernal LJJ, et al. A cross-sectional study of the impact of regular use of insecticides in dogs on canine leishmaniosis seroprevalence in southeast Spain. Prev Vet Med. 2016;124: 78–84.

388. Hernández-Torres A, García-Vázquez E, Frías-Iniesta J, Herrero-Martínez JA, Gómez-Gómez J. Cutaneous leishmaniasis in a patient receiving infliximab. Scand J Infect Dis. 2013;45: 567–569.

389. Hernández-Torres A, García-Vázquez E, Bravo-Urbieta J, Bernal Morell E, Alcaraz-Vidal B, Sánchez-Serrano A, et al. Visceral leishmaniasis in Murcia: Multicentric study 1997-2013. Infectio. 2015;19: 24–30.

390. Jimeno A, Morales E, Peñalver E, Ladrón De Guevara S. Visceral leishmaniasis diagnosed from a colon biopsy. Enferm Infecc Microbiol Clin. 2012;30: 353–354.

391. Moreno Martínez MJ, Moreno Ramos MJ, Sánchez Pedreño P. Cutaneous leishmaniasis: An opportunistic infection. Reumatol Clin. 2017;13: 181–182.

392. Ortuño M, Latrofa MS, Iborra MA, Pérez-Cutillas P, Bernal LJ, Risueño J, et al. Genetic diversity and phylogenetic relationships between *Leishmania infantum* from dogs, humans and wildlife in south-east Spain. Zoonoses Public Health. 2019;66: 961–973.

393. Pardo-Marin L, Ceron JJ, Tecles F, Baneth G, Martínez-Subiela S. Comparison of acute phase proteins in different clinical classification systems for canine leishmaniosis. Vet Immunol Immunopathol. 2020;219: 109958.

394. Pérez-Cutillas P, Goyena E, Chitimia L, Rúa P, Bernal LJ, Fisa R. Spatial distribution of human asymptomatic *Leishmania infantum* infection in southeast Spain: a study of environmental, demographic and social risk factors. Acta Trop. 2015;146: 127–134.

395. Risueño J, Ortuño M, Pérez-Cutillas P, Goyena E, Maia C, Cortes S, et al. Epidemiological and genetic studies suggest a common *Leishmania infantum* transmission cycle in wildlife, dogs and humans associated to vector abundance in Southeast Spain. Vet Parasitol. 2018;259: 61–67.

396. Sebastián-Marcos P, Santarelli G, Gómez S, Palacio MJFD. Canine leishmaniasis associated with pericardial effusion in a 4-year-old dog. J Vet Cardiol. 2019;23: 32–37.

397. Vargas Acosta TM, Belchí Segura E, Martinez Caselles A, Baños Madrid R, Pons Miñano JA, Parrilla Paricio P. Diarrhea due to visceral leishmaniasis in a liver transplant recipient. Gastroenterol Hepatol. 2013;36: 271–273.

398. Strelkova MV, Ponirovsky EN, Morozov EN, Zhirenkina EN, Razakov SA, Kovalenko DA, et al. A narrative review of visceral leishmaniasis in Armenia, Azerbaijan, Georgia, Kazakhstan, Kyrgyzstan, Tajikistan, Turkmenistan, Uzbekistan, the Crimean Peninsula and Southern Russia. Parasit Vectors. 2015;8: 330.
